# Thyrotropin exacerbates insulin resistance by triggering macrophage inflammation in subclinical hypothyroidism

**DOI:** 10.1038/s12276-025-01478-1

**Published:** 2025-06-16

**Authors:** Haihong Zhang, Zekun Zeng, Yan Liu, Wenfang Zheng, Jianling Wang, Yao Yao, Yanan Wang, Meiju Ji, Peng Hou

**Affiliations:** 1 Department of Endocrinology and International Joint Research Center for Tumor Precision Medicine of Shaanxi Province, The First Affiliated Hospital of Xi’an Jiaotong University, Xi’an, China; 2 Med-X Institute, Center for Immunological and Metabolic Diseases, and Department of Endocrinology, First Affiliated Hospital of Xi’an Jiaotong University, Xi’an, China; 3 Center for Translational Medicine, The First Affiliated Hospital of Xi’an Jiaotong University, Xi’an, China

**Keywords:** Type 2 diabetes, Thyroid diseases

## Abstract

In subclinical hypothyroidism, the levels of serum thyroid-stimulating hormone (TSH) are positively correlated with insulin resistance; however, the precise mechanism is unclear. Except for thyroid follicular epithelial cells, macrophages express the highest levels of *TSHR*. Thus, we speculate that TSH may promote insulin resistance by triggering macrophage inflammation. Here we established a mouse model of TSH receptor (*Tshr*) myeloid-specific knockout (*Tshr*^*MKO*^) and found that *Tshr*^*MKO*^ mice showed improvement on high-fat diet-induced obesity and insulin resistance compared with wild-type mice (*Tshr*^*f/f*^). In addition, *Tshr*^*MKO*^ mice exhibited decreased infiltration and M1 polarization of macrophages in liver, adipose and skeletal muscle. Co-culture experiments proved that *Tshr*-deficient macrophages decreased gluconeogenesis in hepatocytes but increased glucose uptake in adipocytes and skeletal muscle cells by improving the insulin signaling pathway. Mechanistically, increased TSH levels in subclinical hypothyroidism promoted the secretion of cytokines IL-1α, IL-1β and IL-6 by inducing macrophage M1 polarization, which upregulated *EGR1* to transcriptionally activate *LCN2* and *SOCS3* in insulin target cells, thereby exacerbating insulin resistance. These effects could be reversed by IL-1 and IL-6 blockers IL-1RA and IL-6ST. Thus, we provided mechanistic insights into the predisposition to insulin resistance in subclinical hypothyroidism and revealed the role of TSH in metabolic disorders.

## TSH induces macrophage inflammation linked to insulin resistance

Subclinical hypothyroidism is a condition in which the thyroid-stimulating hormone (TSH) levels are high, but thyroid hormone levels are normal, and the levels of serum TSH are positively correlated with insulin resistance. Researchers investigated this by using mice with a specific genetic modification that affects TSH receptors in macrophages. The study found that high TSH levels can cause these macrophages to become more inflammatory, which, in turn, worsens insulin resistance. The researchers used a mouse model to show that, when TSH receptors were removed from macrophages, the mice had better insulin sensitivity and less inflammation. This suggests that TSH plays a role in promoting inflammation and insulin resistance. The findings indicate that managing TSH levels in patients with subclinical hypothyroidism could help to reduce their risk of developing insulin resistance and related conditions such as type 2 diabetes.

This summary was initially drafted using artificial intelligence, then revised and fact-checked by the author.

## Introduction

Subclinical hypothyroidism (SH) is characterized by increased serum thyroid-stimulating hormone (TSH) levels with normal free thyroxine (also known as Tetraiodothyronine, T4) levels^1^. The incidence of SH varies among populations, ranging from 3% to 15%, with a higher incidence associated with increasing age, female sex and a suboptimal iodine status^2,3^. SH is caused by autoimmune thyroid disease in the majority of cases^4^. Patients can be asymptomatic and, therefore, undiagnosed and untreated, leading to important adverse events^5,6^. Hypothyroidism has been well recognized to be accompanied by insulin resistance. Traditionally, this is attributed to the decreased thyroid hormone levels in these patients^7^. However, patients with SH also have an elevated risk of insulin resistance or type 2 diabetes mellitus (T2DM)^8–10^. Individual changes in TSH, even within the normal reference range, are an additional risk factor of T2DM^11^. In SH, thyroid hormone levels remain normal and only TSH levels are increased. This suggests that TSH may play a critical role in insulin resistance independent of its effect on thyroid hormones.

In the hypothalamus–pituitary–thyroid axis, TSH, also known as thyrotropin, is widely known for its traditional function of stimulating thyroid hormone synthesis and secretion from the thyroid gland^12^. In addition to thyroid follicular cells expressing high levels of TSH receptor (*TSHR*), other types of cell also express *TSHR*, including macrophages^13^. This suggests that its function is not limited to controlling thyroid function. Previous studies have indicated that the levels of hypersensitive C-reactive protein are significantly higher in patients with SH than in controls with normal thyroid function^14^, which are decreased after treatment with l-thyroxine^15,16^. Moreover, increased TSH levels can promote macrophage M1 polarization^17^ and activate macrophage inflammation by G13- and G15-dependent pathways^13^. TSH can also aggravate atherosclerosis by promoting macrophage inflammation in plaques^18^. M1-polarized macrophages have been shown to be critically involved in insulin resistance^19,20^. Therefore, we speculate whether increased TSH levels can aggravate insulin resistance in SH by promoting macrophage M1 polarization.

In the present study, we tested the above scientific hypothesis by demonstrating the causal relationship between TSH and insulin resistance using myeloid-specific *Tshr*-deficient mice. Specifically, TSH promoted the synthesis and secretion of IL-1α, IL-1β and IL-6 by inducing macrophage M1 polarization. These cytokines upregulated *EGR1* expression in hepatocytes, adipocytes and skeletal myocytes to transcriptionally activate its downstream targets *LCN2* and *SOCS3*, thus aggravating insulin resistance in SH.

## Materials and methods

Further information about the materials and methods, including statistical analyses, is included in the online Supplementary Information. Information about chemical and biological reagents (Supplementary Table 2), antibodies (Supplementary Table 3), primer and siRNA sequences (Supplementary Tables 4–6) and patients with SH and healthy controls (Supplementary Table 7) is also included in the online Supplementary Information.

## Results

### A mouse model of myeloid *Tshr* deficiency is established

Given that TSH exerts its biological function by interacting with its receptor (*TSHR*), we first analyzed the expression of *TSHR* in different types of tissues or cells using the human protein atlas database. The results showed that *TSHR* expression was the highest in thyroid follicular cells, followed by macrophages (Supplementary Fig. 1a). Next, we validated this finding in C57BL/6N mice using quantitative real-time polymerase chain reaction (qRT-PCR) and western blotting assays, showing that *TSHR* expression in macrophages was much higher than that of adipose tissue, skeletal muscle and liver (Supplementary Fig. 1b,c). To determine the effect of the TSH–TSHR signaling pathway in macrophages on insulin sensitivity, we intercrossed mice bearing a conditional loxP-flanked (‘floxed’) allele of *Tshr* (*Tshr*^*f/f*^, used as a wild-type control) with the Lysozyme 2-Cre (Lyz2-Cre) line^13^ to create myeloid-specific Tshr-knockout (*Tshr*^*MKO*^) C57BL/6N mice (Supplementary Fig. 1d). *Tshr*^*MKO*^ mice were born in a Mendelian ratio, and no defective developmental phenotypes were observed between genotypes.

**Fig. 1:**
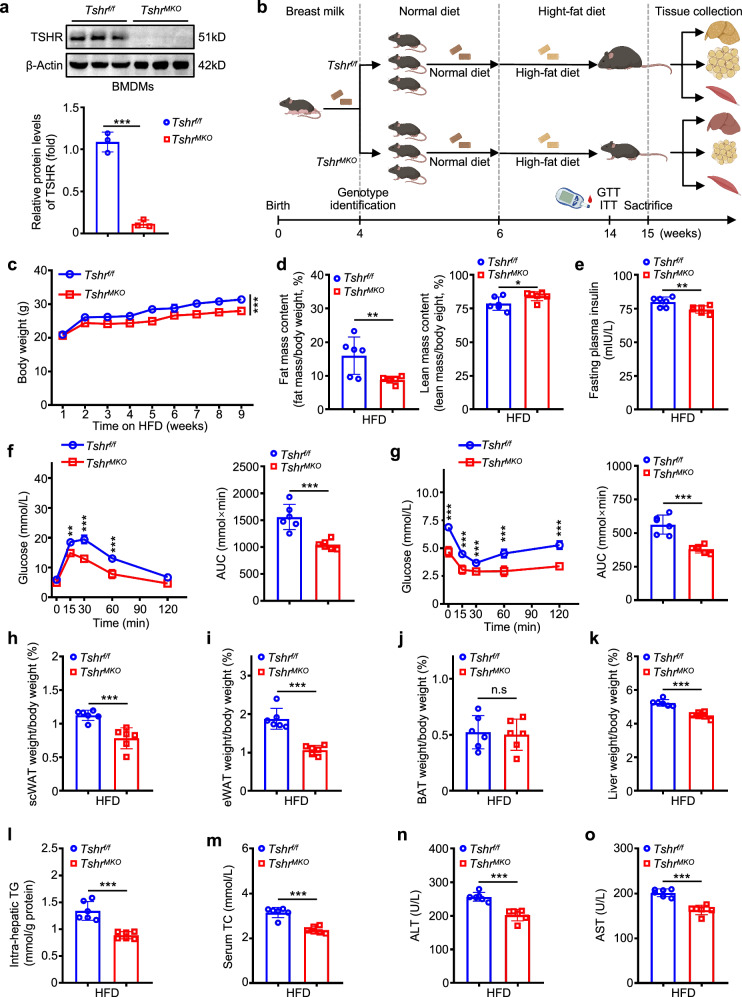
Myeloid *Tshr* deficiency improves HFD-induced insulin resistance and glucose intolerance. **a** The protein levels of *TSHR* in BMDMs from *Tshr*^*MKO*^ mice and age-matched *Tshr*^*f/f*^ littermates were determined by western blotting analysis (*n* = 3). **b** Schedule of the experiment. *Tshr*^*MKO*^ mice and age- and sex-matched *Tshr*^*f/f*^ littermates (male, 6 weeks old) were fed a HFD for 9 weeks. GTT and ITT were tested at 14 weeks of age. At 15 weeks of age, tissues were collected. **c** Growth curves of body weight in HFD-fed *Tshr*^*MKO*^ and *Tshr*^*f/f*^ mice (*n* = 6). **d** EchoMRI was used to measure the percentage of fat (left) and lean (right) body mass in HFD-fed *Tshr*^*MKO*^ and *Tshr*^*f/f*^ mice (*n* = 6). **e** The levels of fasting plasma insulin were measured in the above mice after 8-h fasting (*n* = 6). **f** GTT (left) and area under the curve (AUC, right) in HFD-fed *Tshr*^*MKO*^ and *Tshr*^*f/f*^ mice (*n* = 6). **g** ITT (left) and AUC (right) in HFD-fed *Tshr*^*MKO*^ and *Tshr*^*f/f*^ mice (*n* = 6). Two groups of mice on HFD for 9 weeks were euthanized after 8-h fasting. **h**–**k** The percentages of scWAT (**h**), eWAT (**i**), BAT (**j**) and liver weights to body weight (**k**) in HFD-fed *Tshr*^*MKO*^ and *Tshr*^*f/f*^ mice (*n* = 6). **l**–**o** Intrahepatic TG contents (**l**) as well as serum levels of total cholesterol (TC) (**m**), ALT (**n**) and AST (**o**) were measured using the respective commercial kits (*n* = 6). Data are presented as mean ± standard error of the mean (s.e.m.) (**c**, **f** and **g**) and as mean ± s.d. (**a**, **d**, **e** and **h**–**o**). *P < 0.05, **P < 0.01, ***P < 0.001; ns, not significant (two-way analysis of variance (ANOVA) for **c**, **f** and **g**; unpaired two-tailed Student’s *t*-test for **a**, **d**, **e** and **h**–**o**).

We next performed western blotting analysis and immunofluorescent staining to demonstrate that *TSHR* was efficiently ablated in bone-marrow-derived macrophages (BMDMs) from *Tshr*^*MKO*^ mice (Fig. 1a and Supplementary Fig. 1e). Moreover, we detected *TSHR* protein levels in liver, adipose tissue, skeletal muscle, brain, testis and ovary of *Tshr*^*MKO*^ and *Tshr*^*f/f*^ mice by western blotting analysis. The results showed that its levels in these samples were very low and not significantly different between *Tshr*^*MKO*^ and *Tshr*^*f/f*^ mice (Supplementary Fig. 2a). This essentially rules out the possibility that off-target effects can affect metabolic parameters. In addition, to further exclude the possibility that thyroid hormones interfere with the endpoints of *Tshr*^*MKO*^ mice, we measured the levels of T3, free T4 and TSH in liver, adipose, skeletal muscle tissue and serum of *Tshr*^*MKO*^ and *Tshr*^*f/f*^ mice by enzyme-linked immunosorbent assay (ELISA) and did not find a significant difference in these indicators between *Tshr*^*MKO*^ and *Tshr*^*f/f*^ mice (Supplementary Fig. 2b–d). We also examined thyroid hormone receptor α (THRα) levels in liver, adipose and skeletal muscle tissues of these mice by western blotting analysis and similarly failed to find significant differences between *Tshr*^*MKO*^ and *Tshr*^*f/f*^ mice (Supplementary Fig. 2e). These findings, taken together, indicate that *Tshr* knockout in myeloid cells has no effect on thyroid function in mice.

### Myeloid *Tshr* deficiency protects against HFD-induced insulin resistance and glucose intolerance

*Tshr*^*MKO*^ mice and sex- and age-matched wild-type littermates (*Tshr*^*f/f*^ mice) were fed with high-fat diet (HFD) beginning at 6 weeks of age. Glucose tolerance test (GTT) and insulin tolerance test (ITT) were measured at 14 weeks of age, and tissues of 15-week-old mice were then collected after euthanasia (Fig. 1b). After 9 weeks of HFD feeding, male *Tshr*^*MKO*^ mice exhibited lower body weights (Fig. 1c) and fat mass (Fig. 1d, left), accompanied by significantly increased total lean mass (Fig. 1d, right). However, there was no significant difference in food intake and water drinking between male *Tshr*^*MKO*^ and *Tshr*^*f/f*^ mice (Supplementary Fig. 3a,b). Compared with male *Tshr*^*f/f*^ mice, the levels of fasting insulin were decreased in HFD-fed male *Tshr*^*MKO*^ mice (Fig. 1e). Consistently, HFD-fed male *Tshr*^*MKO*^ mice showed greatly improved glucose tolerance (Fig. 1f) and insulin sensitivity (Fig. 1g) compared with male *Tshr*^*f/f*^ mice. After euthanasia, we collected and weighed fat tissues from male *Tshr*^*MKO*^ and *Tshr*^*f/f*^ mice. The results showed that, compared with male *Tshr*^*f/f*^ mice, fat weights were significantly reduced in male *Tshr*^*MKO*^ mice, including subcutaneous white adipocyte tissue (scWAT) (Fig. 1h and Supplementary Fig. 4a) and epididymal white adipocyte tissue (eWAT) (Fig. 1i and Supplementary Fig. 4b). However, brown adipose tissue (BAT) did not significantly change between two groups (Fig. 1j and Supplementary Fig. 4c).

We next examined the effect of myeloid *Tshr* deficiency on lipid metabolism in the liver, and found that male *Tshr*^*MKO*^ mice had lower liver weights (Fig. 1k and Supplementary Fig. 4d) and intrahepatic triglyceride (TG) contents (Fig. 1l) than male *Tshr*^*f/f*^ mice. Likewise, male *Tshr*^*MKO*^ mice also had lower levels of serum total cholesterol (Fig. 1m), alanine transaminase (ALT) (Fig. 1n) and aspartate transaminase (AST) (Fig. 1o) than male *Tshr*^*f/f*^ mice. Expectedly, in comparison with female *Tshr*^*f/f*^ mice, HFD-fed female *Tshr*^*MKO*^ mice exhibited phenotypic changes similar to those of male mice, including lower body weights (Supplementary Fig. 5a), consistent food intake (Supplementary Fig. 5b) and water drinking (Supplementary Fig. 5c), improved glucose tolerance (Supplementary Fig. 5d,e) and insulin sensitivity (Supplementary Fig. 5f,g). The above results suggest that the TSH–TSHR signaling pathway in macrophages plays a pivotal role in insulin resistance.

### Myeloid *Tshr* deficiency improves insulin signaling pathway in HFD-fed mice

**Fig. 2:**
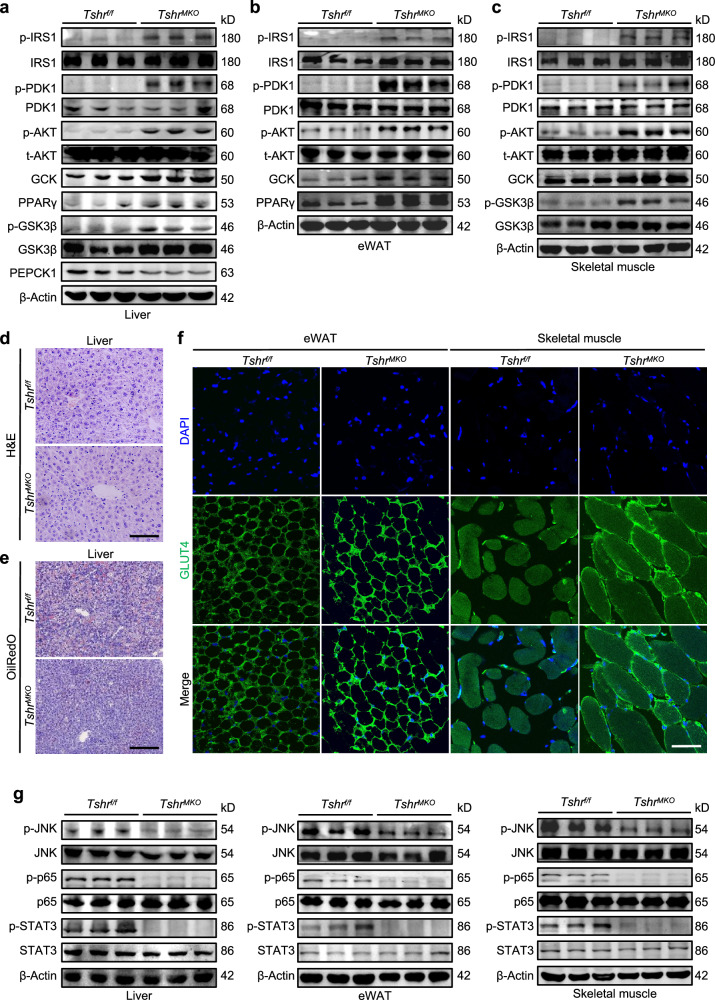
Myeloid *Tshr* deficiency enhances insulin sensitivity by improving the insulin signaling pathway in HFD-fed mice. *Tshr*^*MKO*^ mice and age-matched *Tshr*^*f/f*^ littermates (male, 6 weeks old) were fed with HFD for 9 weeks. They were then euthanized upon insulin administration (1.5 U/kg) for 5 min after 8-h fasting. **a**–**c** Next, western blotting analysis was used to determine the levels of p-IRS1, IRS1, p-PDK1, PDK1, p-AKT, total AKT (t-AKT), GCK, PPARγ, p-GSK3β, GSK3β and PEPCK1 in liver tissues (**a**), the levels of p-IRS1, IRS1, p-PDK1, PDK1, p-AKT, t-AKT, GCK and PPARγ in eWAT (**b**) and the levels of p-IRS1, IRS1, p-PDK1, PDK1, p-AKT, t-AKT, GCK, p-GSK3β and GSK3β in skeletal muscle tissues (**c**). β-Actin was used as a loading control. **d**,**e** Representative H&E (**d**) and Oil Red O staining (**e**) of liver sections. Scale bars, 100 μm. **f** Representative immunofluorescence staining of GLUT4 (green) in eWAT and skeletal muscle sections. Nuclei were stained with DAPI (blue). Scale bars, 50 μm. **g** Western blotting analysis was used to determine the levels of p-JNK, p-p65 and p-STAT3 in liver, eWAT and skeletal muscle. β-Actin was used as a loading control.

We extracted protein lysates from liver, eWAT and skeletal muscle tissues of HFD-fed male *Tshr*^*MKO*^ and *Tshr*^*f/f*^ mice and determined the effect of myeloid *Tshr* deficiency on the activity of the insulin signaling pathway. As shown in Fig. 2a–c, we found that the levels of insulin signaling-related molecules such as phosphorylated insulin receptor substrate 1 (p-IRS1), phosphorylated phosphoinositide-dependent protein kinase 1 (p-PDK1), phosphorylated AKT (S473) (p-AKT) and glycolysis-associated glucokinase (GCK) were significantly increased in liver, eWAT and skeletal muscle tissues of *Tshr*^*MKO*^ mice compared with *Tshr*^*f/f*^ mice. In addition, the levels of peroxisome proliferator-activated receptor γ (PPARγ) protein, which promotes conversion of glucose to fat stimulated by insulin, were obviously increased in liver and eWAT tissues of HFD-fed *Tshr*^*MKO*^ mice (Fig. 2a, b). Consistently, the levels of phosphorylated glycogen synthase kinase 3β (p-GSK3β), associated with glycogen synthesis, were upregulated in liver and skeletal muscle tissues of HFD-fed *Tshr*^*MKO*^ mice (Fig. 2a, c). The levels of phosphoenolpyruvate carboxykinase 1 (PEPCK1) as the rate-limiting enzyme of gluconeogenesis, which can be inhibited by insulin, were downregulated in liver tissues of HFD-fed *Tshr*^*MKO*^ mice (Fig. 2a).

On liver sections of HFD-fed male *Tshr*^*MKO*^ mice, the positive areas of lipid droplets (Fig. 2d and Supplementary Fig. 6a) and Oil Red O staining (Fig. 2e and Supplementary Fig. 6b) were significantly reduced in comparison with HFD-fed male *Tshr*^*f/f*^ mice, indicating that *Tshr*^*MKO*^ mice were obviously protected from the HFD-induced hepatic steatosis. In addition, the membrane localization of *GLUT4*, a transporter protein important for glucose uptake controlled by insulin, was enhanced in eWAT and skeletal muscle tissues of HFD-fed male *Tshr*^*MKO*^ mice compared with HFD-fed male *Tshr*^*f/f*^ mice (Fig. 2f and Supplementary Fig. 7). The above findings indicate that myeloid *Tshr* knockout effectively improves the intracellular insulin signaling pathway in liver, eWAT and skeletal muscle tissues of HFD-fed mice.

Under inflammatory activation, proinflammatory chemokines lead to the activation of c-Jun N-terminal kinase (JNK), nuclear factor kappa B (NF-κB) and signal transducer and activator of transcription 3 (STAT3) in liver, adipose and skeletal muscle cells, interfering with the normal insulin signaling pathway^21–28^. Thus, we examined the effect of myeloid *Tshr* deficiency on the levels of these insulin resistance-related molecules. The results showed that the levels of p-JNK, p-p65 and p-STAT3 were downregulated in liver, eWAT and skeletal muscle tissues of HFD-fed *Tshr*^*MKO*^ mice (Fig. 2g). Similarly, p-p65 staining in the nucleus of eWAT and skeletal muscle sections of *Tshr*^*MKO*^ mice was also distinctly reduced compared with *Tshr*^*f/f*^ mice (Supplementary Fig. 8a–c). These results suggest that myeloid *Tshr* deficiency inhibits the inflammatory signaling pathway, further improving insulin resistance in HFD-fed mice.

### Myeloid *Tshr* deficiency alleviates macrophage infiltration and M1 polarization in liver, adipose tissue and skeletal muscle of HFD-fed mice and improves insulin resistance

**Fig. 3:**
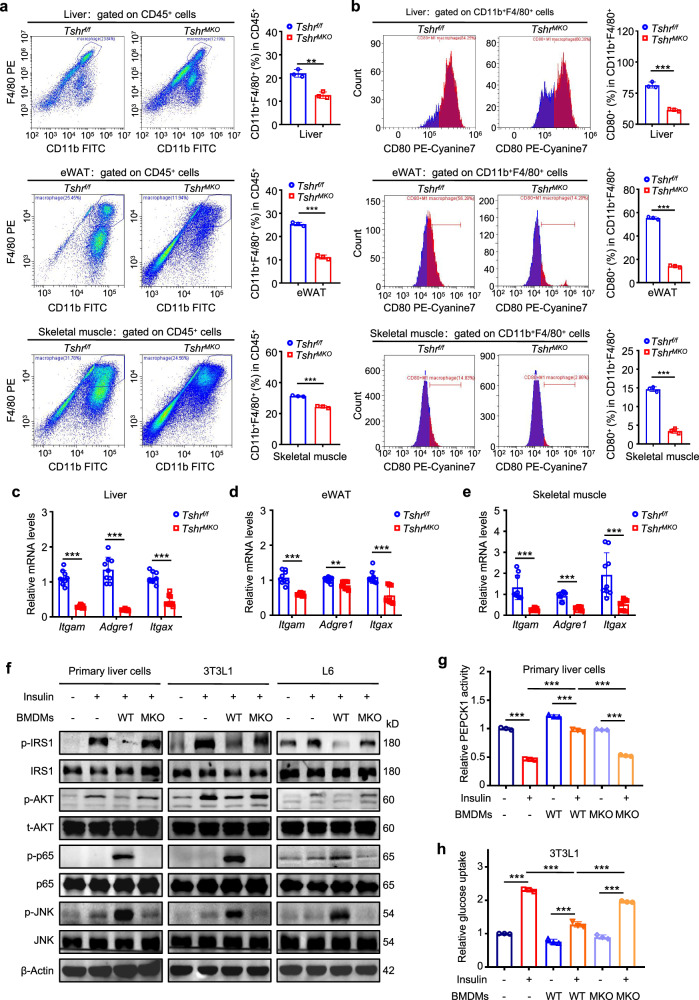
Myeloid *Tshr* deficiency alleviates macrophage infiltration and M1 polarization in liver, adipose tissues and skeletal muscle of HFD-fed mice and improves insulin resistance. Male *Tshr*^*MKO*^ mice and age-matched *Tshr*^*f/f*^ littermates were fed with HFD for 9 weeks. Liver, eWAT and skeletal muscle were isolated from these mice. Flow cytometry was performed to analyze the effect of myeloid *Tshr* deficiency on macrophage infiltration and M1 polarization in liver, eWAT and skeletal muscle. **a** The percentage of CD11b^+^ F4/80^+^ macrophages from the CD45^+^ cell gate in liver, eWAT and skeletal muscle (*n* = 3). **b** The percentage of CD80^+^ macrophages from the CD11b^+^ F4/80^+^ cell gate in liver, eWAT and skeletal muscle (*n* = 3). The mRNA levels of *Itgam*, *Adgre1* and *Itgax* in liver (**c**), eWAT (**d**) and skeletal muscle (**e**) were measured by qRT-PCR (*n* = 9). *β-Actin* was used as an internal control (*n* = 9). Primary hepatocytes, 3T3L1-differentiated adipocytes and L6-differentiated skeletal muscle cells were co-cultivated with *Tshr*^*f/f*^- or *Tshr*^*MKO*^-derived BMDMs for 48 h and then stimulated with 100 nM insulin for 15 min. **f** Western blotting analysis was performed to determine the levels of p-IRS1, IRS1, p-AKT, t-AKT, p-p65, p65, p-JNK and JNK in primary hepatocytes, 3T3L1-differentiated adipocytes and L6-differentiated skeletal muscle cells. β-Actin was used as a loading control. **g** Relative PEPCK1 activity of primary hepatocytes with the indicated treatments (*n* = 3). **h** Relative glucose uptake of 3T3L1-differentiated adipocytes with the indicated treatments (*n* = 3). Data are presented as mean ± s.d. *P < 0.05, **P < 0.01, ***P < 0.001 (unpaired two-tailed Student’s *t*-test for **a** and **b**; one-way ANOVA for **c**–**e**, **g** and **h**).

Macrophage M1 polarization is an important pathogenesis of insulin resistance^19,29,30^. To determine whether insulin resistance is associated with TSH-mediated macrophage infiltration and M1 polarization, we examined the changes in inflammatory cell infiltration of insulin target tissues. First, we observed that HFD-fed *Tshr*^*MKO*^ mice had less infiltration of immune cells in scWAT, eWAT and skeletal muscle except for BAT compared with *Tshr*^*f/f*^ mice by hematoxylin and eosin (H&E) staining (Supplementary Fig. 9). Next, we used fluorescence-activated cell sorting analysis to determine the effect of myeloid *Tshr* deficiency on infiltration and M1 polarization of macrophages infiltrating into livers, eWAT and skeletal muscle. The results revealed that the quantity of both CD11b^+^F4/80^+^ macrophages and CD80^+^ M1-polarized macrophages was reduced in the above tissues of HFD-fed *Tshr*^*MKO*^ mice compared with *Tshr*^*f/f*^ mice (Fig. 3a,b). Likewise, we demonstrated by immunofluorescence staining that eWAT and skeletal muscle of HFD-fed *Tshr*^*MKO*^ mice had less infiltrated CD11b^+^F4/80^+^ macrophages and CD11b^+^CD86^+^ M1-polarized macrophages than those of *Tshr*^*f/f*^ mice (Supplementary Fig. 10). In addition, the mRNA levels of *Itgam*, *Adgre1* and *Itgax*, which encode CD11b, F4/80 and CD11c, respectively, were also strikingly downregulated in liver, adipose tissue and skeletal muscle of *Tshr*^*MKO*^ mice compared with *Tshr*^*f/f*^ mice (Fig. 3c–e), further supporting the above results. Collectively, myeloid *Tshr* deficiency reduces the infiltration and M1 polarization of macrophages, which maybe be involved in insulin resistance in HFD-fed mice.

It has been well established that macrophages contribute to insulin resistance in liver, adipose tissue and skeletal muscle via the secretion of cytokines and exosomes^31,32^. To examine the causal relationship of myeloid *Tshr* deficiency and insulin resistance, we isolated BMDMs from *Tshr*^*f/f*^ or *Tshr*^*MKO*^ mice and constructed co-cultivation system with primary hepatocytes, 3T3L1-differentiated adipocytes or L6-differentiated skeletal muscle cells, respectively. In brief, BMDMs were plated in the upper chamber of transwells and insulin target cells in the lower chamber. The transwells with 0.4 µm pore size allowed the passage of only cytokines and exosomes, but the cells could not pass freely. The results showed that the levels of p-IRS1 and p-AKT were increased and the levels of p-p65 and p-JNK were decreased when primary hepatocytes, 3T3L1-differentiated adipocytes or L6-differentiated skeletal muscle cells were co-cultured with *Tshr*^*MKO*^-derived BMDMs compared with *Tshr*^*f/f*^-derived BMDMs (Fig. 3f). Moreover, our data demonstrated that co-culture of primary hepatocytes with *Tshr*^*f/f*^-derived BMDMs reversed the inhibitory effect of insulin on the activity of PEPCK1, while this effect was significantly attenuated when primary hepatocytes were co-cultivated with *Tshr*^*MKO*^-derived BMDMs (Fig. 3g). In addition, we found that the promoting effect of insulin on glucose uptake of adipocytes and skeletal muscle cells was dramatically impaired when they were co-cultivated with *Tshr*^*f/f*^-derived BMDMs, which could be restored by myeloid *Tshr* knockout (Fig. 3h and Supplementary Fig. 11). These results, taken together, suggest that cytokines or exosomes secreted from macrophages activated by the TSH–TSHR signaling pathway may be related to insulin resistance in HFD-fed mice.

### TSH governs proinflammatory signaling and promotes the secretion of IL-1α, IL-1β and IL-6 in macrophages

**Fig. 4:**
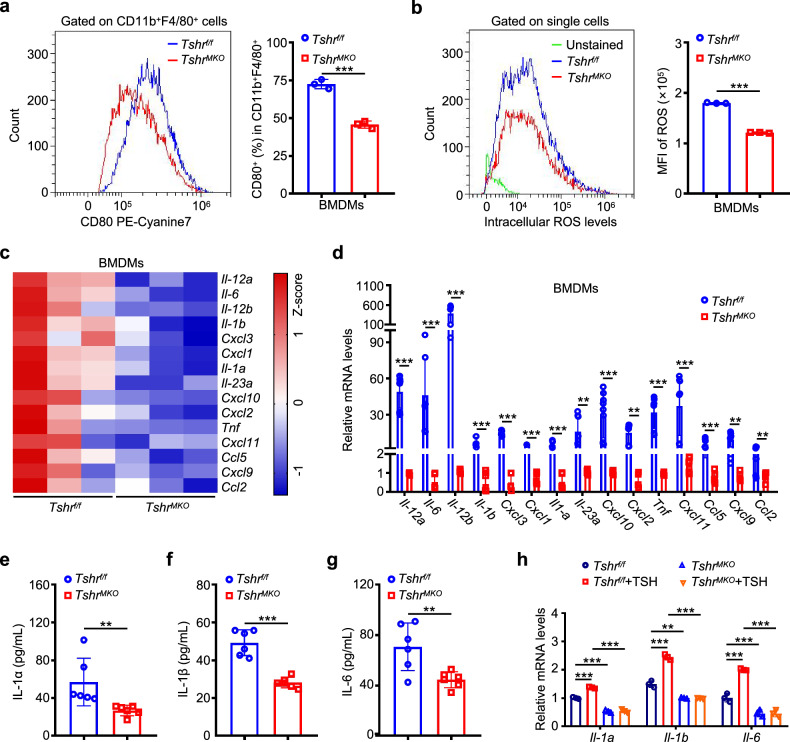
Myeloid *Tshr* deficiency suppresses M1 polarization of macrophages and the secretion of IL-1α, IL-1β and IL-6. *Tshr*^*f/f*^- or *Tshr*^*MKO*^-derived BMDMs were stimulated by 1 ng/mL TSH for 24 h, and flow cytometry was then performed to analyze the effects of myeloid *Tshr* deficiency on M1 polarization of macrophages and intracellular ROS levels. **a** CD80^+^ M1 macrophages from the CD11b^+^ F4/80^+^ cell gate (*n* = 3). **b** Intracellular ROS levels (left) and mean fluorescence intensity (MFI) of ROS (right) (*n* = 3). **c** A heatmap of the proinflammatory cytokines in *Tshr*^*f/f*^- or *Tshr*^*MKO*^-derived BMDMs determined by mRNA sequencing. **d** The mRNA levels of the proinflammatory cytokines in the above BMDMs were measured by qRT-PCR. *β-Actin* was used as an internal control (*n* = 9). *Tshr*^*MKO*^ mice and age-matched *Tshr*^*f/f*^ littermates (male, 6 weeks old) were fed with HFD for 9 weeks. Serum concentrations of IL-1α (**e**), IL-1β (**f**) and IL-6 (**g**) in these mice were then measured by ELISA (*n* = 6). **h** qRT-PCR assays were performed to determine mRNA levels of *Il-1a*, *Il-1b* and *Il-6* in *Tshr*^*f/f*^- or *Tshr*^*MKO*^-derived BMDMs stimulated by 1 ng/mL TSH for 24 h. *β-Actin* was used as an internal control (*n* = 3). Data are presented as mean ± s.d. **P < 0.01, ***P < 0.001 (unpaired two-tailed Student’s *t*-test for **a**, **b** and **e**–**g**; one-way ANOVA for **d** and **h**).

To determine the effect of the TSH–TSHR signaling pathway on macrophages, inflammatory markers were examined in BMDMs by flow cytometry. The results showed that *Tshr*^*MKO*^-derived BMDMs displayed significantly lower levels of CD80 and ROS than *Tshr*^*f/f*^-derived BMDMs (Fig. 4a, b), indicating that the M1 polarization of macrophages induced by TSH was blocked by *Tshr* knockout. Furthermore, to clarify the signaling pathways and specific molecules of macrophages stimulated by TSH, we performed mRNA sequencing and Gene Ontology (GO) term enrichment analysis in *Tshr*^*MKO*^- and *Tshr*^*f/f*^-derived BMDMs. The results indicated that differential genes were enriched in the TNF signaling pathway, NF-κB signaling pathway and AGE–RAGE signaling pathway in diabetic complications (Supplementary Fig. 12a). Moreover, gene set enrichment analysis (GSEA) of mRNA sequencing data also indicated the activation of the TNF signaling pathway, NOD-like signaling pathway and cytokine–cytokine receptor interaction (Supplementary Fig. 12b). As supported, *Tshr* knockout significantly decreased the levels of p-p65 in TSH-treated BMDMs (Supplementary Fig. 13). Consistently, mRNA levels of many proinflammatory cytokines were substantially decreased in *Tshr*-deficient BMDMs compared with wild-type BMDMs (Fig. 4c), as supported by the results of qRT-PCR (Fig. 4d).

Our mRNA sequencing data also showed that the expression abundance of *Il1r1*, *Il6ra* and *Tnfrsf1a/b* in liver tissues was the highest among the receptors of the above cytokines (Supplementary Table 1). Furthermore, IL-1α, IL-1β, IL-6 and TNF have been indicated to aggravate insulin resistance^33–37^. Thus, we hypothesize that Il-1α, Il-1β, Il-6 and TNF may be responsible for hepatic insulin resistance caused by TSH-induced M1 polarized macrophages. Besides, serum concentrations of IL-1α, IL-1β and IL-6 in HFD-fed *Tsh*^*MKO*^ mice were lower than those of *Tshr*^*f/f*^ mice (Fig. 4e–g). However, there was no significant difference in the levels of TNF between two groups of mice (Supplementary Fig. 14). As supported, we also found by qRT-PCR and ELISA that the mRNA and protein levels of IL-1α, IL-1β and IL-6 were significantly decreased in liver and adipose skeletal muscle of *Tshr*^*MKO*^ mice compared with *Tshr*^*f/f*^ mice (Supplementary Fig. 15a–f). To define the regulatory effect of TSH on *Il-1a*, *Il-1b* and *Il-6* in macrophages, we conducted qRT-PCR assays and demonstrated that TSH substantially upregulated the mRNA levels of *Il-1a*, *Il-1b* and *Il-6* in *Tshr*^*f/f*^-derived BMDMs, and this effect could be reversed by *Tshr* knockout (Fig. 4h). Collectively, our data indicate that TSH activates inflammatory signaling and promotes the secretion of IL-1α, IL-1β and IL-6 in macrophages, thus aggravating insulin resistance.

### Myeloid *Tshr* deficiency improves HFD-induced metabolic disorders in liver

**Fig. 5:**
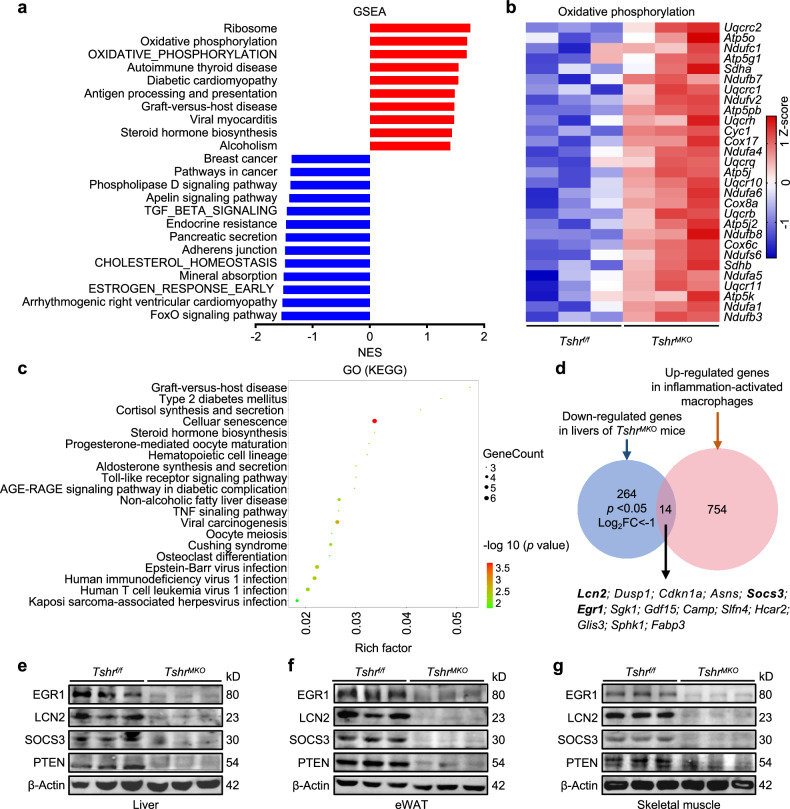
Improvement of HFD-induced metabolic disorders in liver by myeloid *Tshr* deficiency. *Tshr*^*MKO*^ mice and age-matched *Tshr*^*f/f*^ littermates (male, 6 weeks old) were fed with HFD for 9 weeks. They were then euthanized upon insulin administration (1.5 U/kg) for 5 min after 8 h fasting. **a** GSEA of mRNA sequencing data in livers of HFD-fed *Tshr*^*f/f*^ and *Tshr*^*MKO*^ mice (*n* = 3). **b** A heatmap of the oxidative phosphorylation members in livers determined by mRNA sequencing (*n* = 3). **c** GO analysis of differential genes from mRNA sequencing data in livers of HFD-fed *Tshr*^*f/f*^ and *Tshr*^*MKO*^ mice (*n* = 3). **d** A Venn diagram showing overlap of 264 downregulated genes in livers of *Tshr*^*MKO*^ mice and 754 upregulated genes in inflammation-activated macrophages. The protein levels of EGR1, SOCS3, LCN2 and PTEN in liver (**e**), eWAT (**f**) and skeletal muscle (**g**) of HFD-fed *Tshr*^*MKO*^ and *Tshr*^*f/f*^ mice were determined by western blotting analysis. β-Actin was used as a loading control (*n* = 3).

To clarify the mechanism by which TSH-activated macrophages aggravate insulin resistance in insulin target organs, we performed mRNA sequencing of liver tissues of HFD-fed *Tshr*^*f/f*^ and *Tshr*^*MKO*^ mice, which were euthanized after 8-h fasting followed by insulin administration (1.5 U/kg) for 5 min. GSEA analysis of livers mRNA sequencing data indicated the activation of oxidative phosphorylation and the suppression of the FoxO signaling pathway in livers of HFD-fed *Tshr*^*MKO*^ mice compared with *Tshr*^*f/f*^ mice (Fig. 5a). As supported, mRNA levels of oxidative phosphorylation-related genes were distinctly higher in livers of HFD-fed *Tshr*^*MKO*^ mice than those of *Tshr*^*f/f*^ mice (Fig. 5b). GO enrichment analysis indicated that these differential genes were significantly enriched in T2DM, AGE–RAGE signaling pathway in diabetic complications and non-alcoholic fatty liver disease, which are metabolic disorders associated with insulin resistance^38^. In addition, the Toll-like receptor signaling pathway and TNF signaling pathway associated with inflammation were also enriched (Fig. 5c).

To define the specific mechanism by which TSH-activated proinflammatory macrophages induces insulin resistance in liver, 264 downregulated differential genes in liver of *Tshr*^*MKO*^ mice were intersected with 754 upregulated differential genes in inflammation-activated macrophages^39,40^, thereby screening 14 genes (Fig. 5d). Among them, we targeted early growth response 1 (*Egr1*), lipocalin 2 (*Lcn2*) and suppressor of cytokine signaling 3 (*Socs3*), which were relatively high abundant in liver, adipose tissue and skeletal muscle based on human protein atlas and mouse genome informatics database and have been indicated to induce insulin resistance by suppressing the insulin signaling pathway^41–45^. More importantly, *EGR1*^46–49^, *LCN2*^50^ and *SOCS3*^51^ could be upregulated by IL-1α, IL-1β and IL-6 in immune or cancer cells by activating the NF-κB or STAT3 signaling pathway. To validate the above mRNA sequencing results, we examined mRNA expression of *Egr1*, *Lcn2* and *Socs3* in liver, eWAT and skeletal muscle of HFD-fed *Tshr*^*f/f*^ and *Tshr*^*MKO*^ mice and found that these molecules were significantly reduced in these tissues of HFD-fed *Tshr*^*MKO*^ mice compared with *Tshr*^*f/f*^ mice (Supplementary Fig. 16). This was also supported by the results of western blotting (Fig. 5e–g). In addition, the levels of *PTEN* as a downstream target of *EGR1* (ref. ^52^) were decreased in liver, eWAT and skeletal muscle of HFD-fed *Tshr*^*MKO*^ mice in comparison with *Tshr*^*f/f*^ mice (Fig. 5e–g). These results demonstrate the regulatory effect of myeloid *Tshr* deficiency on *EGR1*, *LCN2* and *SOCS3* of insulin target tissues.

### IL-1α, Il-1β and IL-6 from TSH-activated macrophages upregulate *Egr1*, *Lcn2* and *Socs3* and induce insulin resistance in hepatic, adipose and skeletal muscle cells

**Fig. 6:**
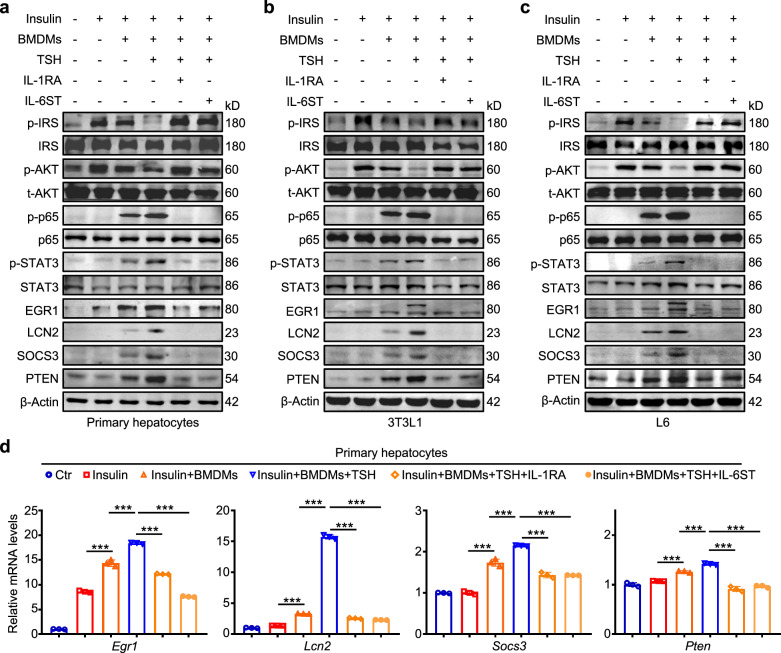
TSH-activated macrophages upregulate *Egr1*, *Lcn2* and *Socs3* and aggravate insulin resistance in hepatic, adipose and skeletal muscle cells via IL-1α, IL-1β and IL-6. Primary hepatocytes, 3T3L1-differentiated adipocytes and L6-differentiated skeletal muscle cells were co-cultivated with wild-type BMDMs stimulated by 1 ng/mL TSH for 24 h and simultaneously treated by 10 ng/mL IL-1RA or IL-6ST for 48 h, followed by 100 nM insulin stimulation for 15 min. **a**–**c** The levels of p-IRS1, IRS1, p-AKT, t-AKT, p-p65, p65, p-STAT3, STAT3, EGR1, LCN2, SOCS3 and PTEN in primary hepatocytes (**a**), 3T3L1-differentiated adipocytes (**b**) and L6-differentiated skeletal muscle cells (**c**) were then determined by western blotting analysis. β-Actin was used as a loading control. **d** The mRNA levels of *Egr1*, *Lcn2*, *Socs3* and *Pten* were determined by qRT-PCR in primary hepatocytes with the indicated treatments. *β-Actin* was used as an internal control (*n* = 3). Data are presented as mean ± s.d. ***P < 0.001 (one-way ANOVA for **d**).

To clarify the causality relationship between cytokines IL-1α, IL-1β and IL-6 from macrophages and insulin resistance, we co-cultured primary hepatocytes with BMDMs and treated them with different combinations of insulin, TSH, IL-1 blocker IL-1RA or IL-6 blocker IL-6ST. The results showed that TSH decreased the levels of insulin signaling pathway-related molecules p-IRS1 and p-AKT while increasing the levels of insulin resistance-related molecules p-p65 and p-STAT3 (Fig. 6a). Expectedly, the protein levels of EGR1, LCN2, SOCS3 and PTEN were upregulated when primary hepatocytes were co-cultured with TSH-stimulated BMDMs, and this effect could be reversed by IL-1 blocker IL-1RA or IL-6 blocker IL-6ST (Fig. 6a), also supported by the results in differentiated 3T3-L1 adipocytes (Fig. 6b) and L6 skeletal muscle cells (Fig. 6c). In addition, we also observed that mRNA levels of *Egr1*, Lcn2, *Socs3* and *Pten* were substantially upregulated in primary hepatocytes co-cultured with TSH-stimulated BMDMs, while this effect could also be reversed by IL-1RA or IL-6ST (Fig. 6d). The above findings indicate that cytokines IL-1α, IL-1β and IL-6 from TSH-activated macrophages upregulate the levels of *EGR1*, *LCN2* and *SOCS3* via the activation of NF-κB and STAT3 signaling pathways, thereby inducing insulin resistance in hepatic, adipose and skeletal muscle cells. However, the regulatory relationship among *EGR1*, *LCN2* and *SOCS3* is still unknown.

### EGR1 transcriptionally activates *LCN*2 and *SOCS3* to aggravate insulin resistance

**Fig. 7:**
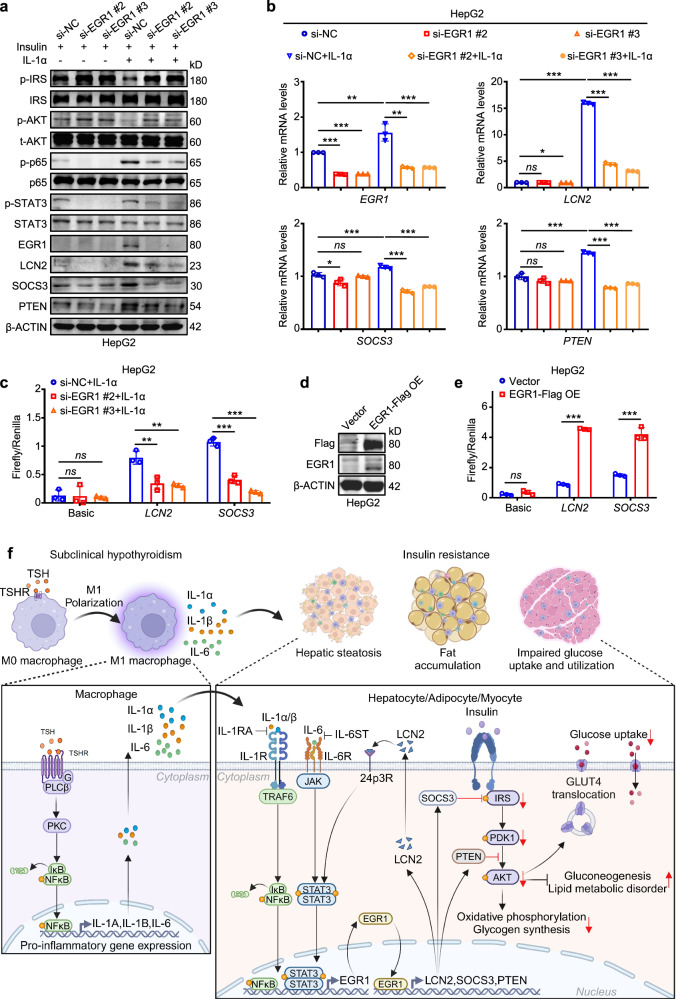
EGR1 aggravates insulin resistance by transcriptionally activating *LCN2* and *SOCS3.* *EGR1*-knockdown HepG2 cells and their control cells were treated with PBS or 10 ng/mL IL-1α for 48 h and then stimulated by 100 nM insulin for 15 min. **a** The levels of p-IRS1, IRS1, p-AKT, t-AKT, p-p65, p65, p-STAT3, STAT3, EGR1, LCN2, SOCS3 and PTEN were determined by western blotting analysis. **b** The mRNA levels of *EGR1*, *LCN2*, *SOCS3* and *PTEN* in HepG2 cells with the indicated treatments (*n* = 3). **c** PGL3.0 plasmids inserted the promoter of *LCN2* or *SOCS3* were co-transfected with pRL-TK plasmid into *EGR1*-knockdown HepG2 cells or control cells, which were treated with 10 ng/mL IL-1α for 48 h. The promoter transcriptional activity of *LCN2* and *SOCS3* was determined by the dual-fluorescence reporter system (*n* = 3). **d** The protein levels of *EGR1* and Flag in *EGR1*-overexpressing HepG2 cells or control cells. **e** PGL3.0 plasmids inserted the promoter of *LCN2* or *SOCS3* were co-transfected with pRL-TK plasmid into *EGR1*-overexpressing HepG2 cells or control cells. The promoter transcriptional activity of *LCN2* and *SOCS3* was determined by the dual-fluorescence reporter system. Luciferase activity was normalized to Renilla luciferase activity (*n* = 3). **f** A schematic model for TSH triggering macrophage inflammation to exacerbate insulin resistance in SH. Data are presented as mean ± s.d. *P < 0.05, **P < 0.01, ***P < 0.001; ns, not significant (one-way ANOVA for **b**, **c** and **e**).

Using the JASPAR database, transcription factor EGR1 was predicted to bind to the promoters of *LCN2* and *SOCS3* (Supplementary Fig. 17a,b, top). It is suggested that EGR1 may transcriptionally regulate *LCN2* and *SOCS3*, thereby impeding the insulin signaling pathway^43,44,53,54^. To prove this, we next knocked down *EGR1* in HepG2 cells stimulated by insulin, and treated *EGR1*-knockdown cells and their control cells with IL-1α. The results showed that *EGR1* knockdown reversed a decrease in the levels of p-IRS1 and p-AKT and an increase in the levels of p-p65, p-STAT3, LCN2, SOCS3 and PTEN induced by IL-1α (Fig. 7a). As expected, we found that *EGR1* knockdown also reversed IL-1α-induced increase in the mRNA levels of *LCN2*, *SOCS3* and *PTEN* (Fig. 7b). The above findings indicate that IL-1α-mediated upregulation of EGR1 promotes insulin resistance probably by transcriptionally activating *LCN2*, *SOCS3* and *PTEN*.

To further determine the transcriptional regulatory effect of EGR1 on *LCN2* and *SOCS3*, we inserted the promoter of *LCN2* or *SOCS3* into PGL3.0 plasmid and co-transfected them with pRL-TK plasmid in HepG2 cells (Supplementary Fig. 17a,b, bottom). We next performed the dual-fluorescence reporter assay and found that *EGR1* knockdown dramatically decreased the promoter activity of *LCN2* and *SOCS3* compared with the control (Fig. 7c). We also ectopically expressed EGR1-Flag in HepG2 cells (Fig. 7d) and demonstrated that *EGR1* overexpression expectedly increased the promoter activity of *LCN2* and *SOCS3* in HepG2 cells (Fig. 7e). Besides, we performed the chromatin immunoprecipitation assays to determine whether EGR1 directly binds to the promoters of *LCN2* and *SOCS3* in HepG2 cells. Immunoprecipitated chromosomal DNA was subjected to quantitative PCR using the primers that were designed to amplify different promoter regions of *LCN2* and *SOCS3* containing the predicted EGR1 binding sites by the JASPAR database (Supplementary Fig. 17a,b). The results showed that the above promoter regions of *LCN2* and *SOCS3* were significantly enriched by anti-Flag antibody in the EGR1-Flag-overexpressing HepG2 cells compared with control cells (Supplementary Fig. 17c,d). These results, taken together, indicate that *LCN2* and *SOCS3* are direct downstream targets of transcription factor EGR1.

In summary, we proposed a model to illustrate how TSH aggravates insulin resistance in SH (Fig. 7f). Specifically, elevated TSH levels in patients with SH activate the NF-κB signaling pathway in macrophages to promote their M1 polarization and the secretion of IL-1α, IL-1β and IL-6. These cytokines cause the upregulation of *EGR1* in liver, adipose and skeletal muscle cells by activating NF-κB and STAT3 signaling pathways. EGR1 as a transcription factor directly activates the transcription of its downstream targets *LCN2*, *SOCS3* and *PTEN* to impair the insulin signaling pathway in insulin target cells. As a result, hepatic oxidative phosphorylation and glycogen synthesis are downregulated, but hepatic gluconeogenesis and lipid metabolic disorders are upregulated, leading to hepatic steatosis and fat accumulation. Also, reduced AKT activity causes a decrease in glucose uptake and utilization in adipose and skeletal muscle cells by impeding the membrane localization of GLUT4, thus aggravating insulin resistance in patients with SH.

### Validation of TSH-induced insulin resistance in patients with SH

**Fig. 8:**
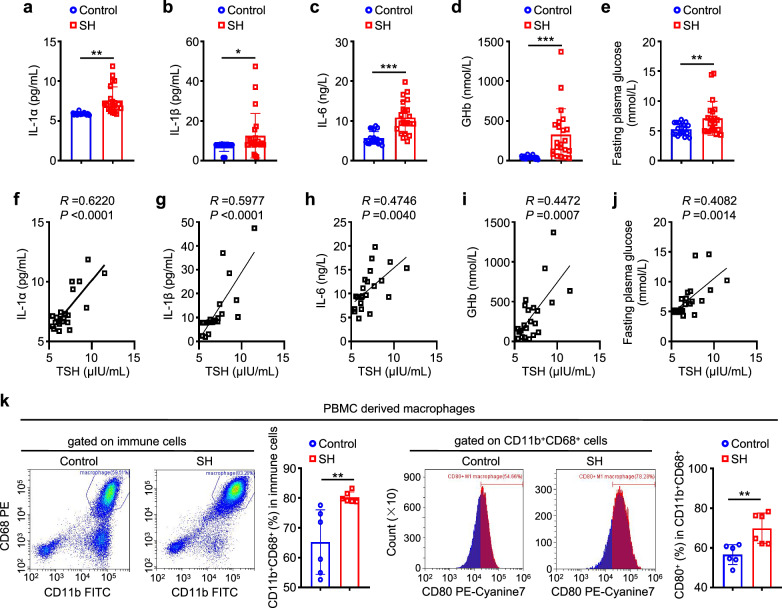
Validation of TSH-triggered insulin resistance in patients with SH. **a**–**e** The levels of IL-1α (**a**), IL-1β (**b**), IL-6 (**c**), GHb (**d**) and FPG (**e**) in the serum from patients with SH and healthy controls (*n* = 20). **f**–**j** Correlations of the levels of IL-1α (**f**), IL-1β (**g**), IL-6 (**h**), GHb (**i**) and FPG (**j**) with TSH levels in patients with SH (*n* = 20). PBMC-derived macrophages were treated with 50 ng/mL of human macrophage colony-stimulating factor (hM-CSF) for 7 days, accompanied by stimulation with the serum from patients with SH and healthy controls. Flow cytometry was then used to determine their effect on macrophage differentiation and M1 polarization. **k** The percentage of CD11b^+^CD68^+^ macrophages from the immune cell gate and CD80^+^ macrophages from the CD11b^+^ CD68^+^ cell gate (*n* = 6). Data are presented as mean ± s.d. *P < 0.05, **P < 0.01, ***P < 0.001 (unpaired two-tailed Student’s *t*-test for **a**–**e** and **k**; Pearson linear correlation analysis for **f**–**j**).

To verify whether patients with SH could have similar patterns in macrophage polarization markers and cytokine levels, we collected peripheral blood samples from patients with SH and healthy controls and measured the levels of IL-1α, IL-1β, IL-6, glycated hemoglobin (GHb) and fasting plasma glucose (FPG). The results showed that the above indexes in patients with SH were higher than those in healthy controls (Fig. 8a–e). Also, we demonstrated that there were positive associations of the levels of IL-1α, IL-1β, IL-6, GHb and FPG with TSH levels in patients with SH (Fig. 8f–j). In addition, we used flow cytometry to evaluate the M1 polarization of peripheral blood mononuclear cell (PBMC)-derived macrophages, which were induced by macrophage colony-stimulating factor and stimulated with the serum from patients with SH or healthy controls. The results showed that the serum from patients with SH more significantly increased the percentage of CD11b^+^CD68^+^ macrophages and CD80^+^ M1 macrophages compared with that from healthy controls (Fig. 8k). Collectively, these findings in human samples strongly support our conclusions from murine models.

## Discussion

Thyroid dysfunction and diabetes mellitus are two of the most frequent chronic endocrine disorders with variable prevalence among different populations^3^. In the long early stages of these diseases, the patients can be asymptomatic and, therefore, undiagnosed and untreated, leading to important adverse events^5,6^. The risk of insulin resistance or T2DM is increased among patients with SH, and the TSH levels, even within the normal reference range, are an additional risk factor of incident T2DM^8–11^. In SH, the levels of thyroid hormone remain normal and only TSH levels are increased. This suggests that TSH may also contribute to insulin resistance independent of its effect on thyroid hormones. In addition, *TSHR* expression was highest in macrophages except for thyroid follicular cells, and M1 polarization of macrophages has been shown to be critically involved in insulin resistance^19,20^. We thus speculate that increased TSH levels may aggravate insulin resistance by promoting M1 polarization of macrophages in SH.

To validate the above scientific hypothesis, we established *Tshr*^*MKO*^ mice to test the effect of the TSH–TSHR signaling pathway in macrophages on insulin sensitivity. Our data indicated a significant decrease of the infiltrated M1 macrophages in liver, adipose and skeletal muscle tissues of HFD-fed *Tshr*^*MKO*^ mice. Moreover, we also found that M1 polarization of macrophages and ROS levels were decreased in *Tshr*-deficient BMDMs despite TSH stimulation. Likewise, the levels of p-p65 were decreased in *Tshr*-deficient BMDMs, which was supported by GO analysis of mRNA sequencing in *Tshr*^*MKO*^- and *Tshr*^*f/f*^-derived BMDMs. The mRNA sequencing data also indicated that the expression of 15 cytokines was different between *Tshr*^*MKO*^- and *Tshr*^*f/f*^-derived BMDMs. Furthermore, among the corresponding receptors of these 15 cytokines, IL-1α/β receptor *IL-1r1*, IL-6 receptor *IL-6ra* and TNF receptor *Tnfrsf1a* have been shown to be the most abundant in liver and to aggravate insulin resistance^33–37^. As supported, serum levels of IL-1α, IL-1β and IL-6 were significantly reduced in HFD-fed *Tshr*^*MKO*^ mice in comparison with control mice.

To gain mechanistic insight into the protective action of myeloid *Tshr* deficiency against HFD-induced insulin resistance, we performed comparative liver transcriptome in HFD-fed *Tshr*^*MKO*^ mice versus *Tshr*^*f/f*^ mice. In the 250 downregulated differentially expressed genes, 14 genes could be upregulated in response to inflammation activation^39,40^. Among them, *Egr1*, *Lcn2* and *Socs3* were more abundant in liver and closely associated with insulin resistance. Previous studies have shown that *EGR1* as a transcription factor induces insulin resistance by activating the transcription of *PTEN*^52^, *PEPCK1*^55^ and *TNFα*^56^. *LCN2* as an inflammatory marker can activate the STAT3 signaling pathway by its receptor 24p3R to exacerbate insulin resistance, nonalcoholic steatohepatitis and obesity^43,54^. *SOCS3* has been demonstrated to induce IRS1 degradation and transform phosphorylation from tyrosine 895 to serine 307, inhibiting Akt phosphorylation and subsequently promoting insulin resistance^44,45^. Besides, in primary hepatocytes, 3T3L1-differentiated adipocytes and L6-differentiated skeletal muscle cells co-cultured with wild-type BMDMs, TSH stimulation caused an increase in the protein and mRNA levels of *Egr1*, *Lcn2* and *Socs3*, which was accompanied by a decrease in the levels of insulin signaling pathway-related molecules such as p-IRS1 and p-AKT and an increase in the levels of insulin resistance-related molecules such as p-p65 and p-STAT3. These effects could be reversed by IL-1α/β blocker IL-1RA or IL-6 blocker IL-6ST.

*EGR1* as a transcription factor was predicted to bind to the promoters of *LCN2* and *SOCS3*. Furthermore, knocking down *EGR1* in HepG2 cells reversed an increase in the expression of *LCN2* and *SOCS3* and levels of insulin resistance-related molecules p-p65 and p-STAT3 as well as a decrease in the levels of insulin signaling pathway-related molecules p-IRS1 and p-AKT, which were induced by IL-1α. In addition, Further studies identified that *LCN2* and *SOCS3* were the downstream targets of *EGR1*. The above findings suggest that IL-1α, IL-1β and IL-6 secreted from M1-polarized macrophages stimulated by abnormally elevated TSH upregulate *EGR1* expression, transcriptionally activating *LCN2* and *SOCS3* and aggravating insulin resistance in hepatic, adipose and skeletal muscle cells. Our study thus provides strong evidence for the link between SH and insulin resistance.

Similar to the results of the present study, a previous study elucidated that TSH aggravated vascular inflammation and thus contributed to atherogenesis^18^. Another study also found that TSH enhanced the activities of NF-κB and the extracellular signal-regulated kinase (ERK)-p38 signaling pathways in macrophages by activating the TSHR-G protein-coupled receptor signaling axis, promoting their M1-type polarization^13^. As supported, increased levels of macrophage infiltration in liver tissues were found in a rat model of thyroidectomy-induced SH^57^. In addition, there is also evidence showing that cytokines such as IL-1α, IL-6, TNF, INF and CCL2 from proinflammatory macrophages impair metabolic homeostasis^58^. These observations further support our conclusions.

What are the clinical implications of these findings? There is increasing evidence indicating that elevated TSH levels in SH are strongly associated with increased risk of developing obesity and type 2 diabetes^8–11^. These findings were confirmed across multiple ethnic groups, underscoring the physiological importance of TSH in metabolic diseases. Furthermore, several clinical studies showed that a progressive increase in TSH was independently associated with the risk of developing T2D regardless of sex and thyroid autoimmunity, indicating that SH and T2DM are not concomitant results of autoimmune disorders^11^. Nonetheless, these clinical studies fail to reveal the cell type and specific mechanisms, which are responsible for insulin resistance. In the present study, the causal relationship between TSH-mediated M1 polarization of macrophages and insulin resistance was demonstrated using myeloid-specific *Tshr*-knockout mice and a co-cultivation system.

Our data also revealed a proinflammatory effect of TSH on macrophages, further confirming its pathophysiological significance in insulin resistance. Thus, the present study will provide mechanistic insights into the predisposition to insulin resistance in SH and come up with a previously unrecognized role of TSH in metabolic disorders. The mechanism of insulin resistance caused by TSH-activated macrophage inflammation suggests that patients with SH should be screened for inflammatory markers and blood glucose to achieve early diagnosis and avoid serious consequences. Our results also suggest that blocking proinflammation cytokines from abnormal macrophages activated by TSH can improve insulin sensitivity and metabolism to prevent the development of T2DM in patients with SH.

We would like to acknowledge the limitations of the present study. Due to the ubiquitous nature of macrophages in tissue distribution, the Lys2–Cre system is expected to mediate the deletion of loxP-floxed *Tshr* gene in macrophages throughout the body. Here, we focused on the TSH–TSHR signaling pathway in the infiltrated macrophages in liver, adipose and skeletal muscle tissues, owing to the fact that infiltrating macrophages in these tissues account for >90% of the total number of macrophages in the body and are the main source of circulating cytokines. The brain also contains macrophages, known as microglial cells, which have been shown to modulate central inflammation, locomotor activity and feeding in mice^59^. Thus, myeloid *Tshr* depletion in other tissues, including the brain, may also affect HFD-induced weight gain and insulin resistance in mice. Another variable is urinary glucose excretion, an alternative route whereby the body can expend calories^60^. It is possible that hyperglycemia will lead to greater expenditure of calories through urinary glucose excretion in HFD-fed mice. This effect may affect the body weight and energy balance of HFD-fed *Tshr*^*MKO*^ and *Tshr*^*f/f*^ mice. Moreover, we have focused on cytokines from TSH-activated macrophages and ignored exosomes, which can also modulate insulin sensitivity^31^.

In conclusion, the present study demonstrates that TSH exacerbates insulin resistance by triggering macrophage inflammation in patients with SH, thus explaining an important clinical phenomenon that patients with SH are more susceptible to insulin resistance. Notably, although we do not observe any changes in food intake and water drinking between male *Tshr*^*MKO*^ and *Tshr*^*f/f*^ mice, more evidence is needed to determine the role of the TSH–TSHR signaling pathway in microglia and its impact on central inflammation and energy homeostasis. Moreover, whether exosomes from TSH-activated macrophages aggravate insulin resistance also needs to be further investigated.

## Supplementary information

Supplementary Information

Raw Imaging

## Data Availability

mRNA sequencing data are available via NCBI Gene Expression Omnibus (GEO GSE275079). Supplementary Information accompanies the Article on the *Experimental & Molecular Medicine* website (http://www.nature.com/emm/).

## Study approval

Protocols for animal research were approved by the Institutional Animal Ethics Committee of Xi’an Jiaotong University. In compliance with the Declaration of Helsinki, patients were anonymously coded for human experimentation involving specimens. The Institutional Review Board and Human Ethics Committee of the First Affiliated Hospital of Xi’an Jiaotong University authorized the study.

## References

[1] Biondi, B., Cappola, A. R. & Cooper, D. S. Subclinical hypothyroidism: a review. *JAMA* **322**, 153–160 (2019). 31287527 10.1001/jama.2019.9052

[2] Vanderpump, M. P. J. et al. The incidence of thyroid disorders in the community: a twenty-year follow-up of the Whickham Survey. *Clin. Endocrinol.* **43**, 55–68 (2008). 10.1111/j.1365-2265.1995.tb01894.x 7641412

[3] Canaris, G. J., Manowitz, N. R., Mayor, G. & Ridgway, E. C. The Colorado Thyroid Disease Prevalence Study. *Arch. Intern. Med.* **160**, 526–534 (2000). 10695693 10.1001/archinte.160.4.526

[4] Surks, M. I. et al. Subclinical thyroid disease. *JAMA* **291**, 228–238 (2004). 14722150 10.1001/jama.291.2.228

[5] Cooper, D. S. & Biondi, B. Subclinical thyroid disease. *Lancet* **379**, 1142–1154 (2012). 22273398 10.1016/S0140-6736(11)60276-6

[6] Biondi, B., Solomon, C. G. & Cooper, D. S. Subclinical hyperthyroidism. *N. Engl. J. Med.* **378**, 2411–2419 (2018). 29924956 10.1056/NEJMcp1709318

[7] Hatziagelaki, E., Paschou, S. A., Schön, M., Psaltopoulou, T. & Roden, M. NAFLD and thyroid function: pathophysiological and therapeutic considerations. *Trends Endocrinol. Metab.* **33**, 755–768 (2022). 36171155 10.1016/j.tem.2022.08.001

[8] Biondi, B., Kahaly, G. J. & Robertson, R. P. Thyroid dysfunction and diabetes mellitus: two closely associated disorders. *Endocr. Rev.* **40**, 789–824 (2019). 30649221 10.1210/er.2018-00163 PMC6507635

[9] Yang, W. et al. Subclinical hypothyroidism increases insulin resistance in normoglycemic people. *Front. Endocrinol.* **14**, 1106968 (2023). 10.3389/fendo.2023.1106968 PMC10358968 37484968

[10] Jun, J. E. et al. TSH increment and the risk of incident type 2 diabetes mellitus in euthyroid subjects. *Endocrine* **55**, 944–953 (2017). 28042645 10.1007/s12020-016-1221-1

[11] Jun, J. E. et al. Association between changes in thyroid hormones and incident type 2 diabetes: a seven-year longitudinal study. *Thyroid* **27**, 29–38 (2017). 27809684 10.1089/thy.2016.0171

[12] Marians, R. C. et al. Defining thyrotropin-dependent and -independent steps of thyroid hormone synthesis by using thyrotropin receptor-null mice. *Proc. Natl Acad. Sci. USA* **99**, 15776–15781 (2002). 12432094 10.1073/pnas.242322099 PMC137792

[13] Yang, C. et al. TSH activates macrophage inflammation by G13- and G15-dependent pathways. *Endocrinology* **162**, bqab077 (2021). 33851697 10.1210/endocr/bqab077

[14] Yu, Y. et al. Subclinical hypothyroidism is associated with elevated high-sensitive C-reactive protein among adult Taiwanese. *Endocrine* **44**, 716–722 (2013). 23468096 10.1007/s12020-013-9915-0

[15] Bayraktar, M. Serum resistin and high sensitive CRP levels in patients with subclinical hypothyroidism before and after l-thyroxine therapy. *Med. Sci. Monit.* **19**, 210–215 (2013). 23518675 10.12659/MSM.883847 PMC3628353

[16] Vudu, S. & Behnke, A. C-reactive protein levels in patients with autoimmune hypothyroidism before and after levothyroxine treatment. *Cureus* **15**, e50848 (2023). 38249164 10.7759/cureus.50848 PMC10798680

[17] Huang, B., Wen, W. & Ye, S. TSH–SPP1/TRβ–TSH positive feedback loop mediates fat deposition of hepatocyte: crosstalk between thyroid and liver. *Front. Immunol.* **13**, 1009912 (2022). 36300106 10.3389/fimmu.2022.1009912 PMC9589424

[18] Yang, C. et al. Thyrotropin aggravates atherosclerosis by promoting macrophage inflammation in plaques. *J. Exp. Med.* **219**, 1182–1198 (2022). 10.1084/jem.20181473 PMC6504213 30940720

[19] Xu, H. et al. Chronic inflammation in fat plays a crucial role in the development of obesity-related insulin resistance. *J. Clin. Invest.* **112**, 1821–1830 (2003). 14679177 10.1172/JCI19451 PMC296998

[20] Olefsky, J. M. & Glass, C. K. Macrophages, inflammation, and insulin resistance. *Annu. Rev. Physiol.* **72**, 219–246 (2010). 20148674 10.1146/annurev-physiol-021909-135846

[21] Huang, T. et al. Adipocyte-derived kynurenine promotes obesity and insulin resistance by activating the AhR/STAT3/IL-6 signaling. *Nat. Commun.* **13**, 3489 (2022). 35715443 10.1038/s41467-022-31126-5 PMC9205899

[22] Hirosumi, J. et al. A central role for JNK in obesity and insulin resistance. *Nature* **420**, 333–336 (2002). 12447443 10.1038/nature01137

[23] Hundal, R. S. et al. Mechanism by which high-dose aspirin improves glucose metabolism in type 2 diabetes. *J. Clin. Invest.* **109**, 1321–1326 (2002). 12021247 10.1172/JCI14955 PMC150979

[24] Shoelson, S. E. Inflammation and insulin resistance. *J. Clin. Invest.* **116**, 1793–1801 (2006). 16823477 10.1172/JCI29069 PMC1483173

[25] Arkan, M. C. et al. IKK-β links inflammation to obesity-induced insulin resistance. *Nat. Med.* **11**, 191–198 (2005). 15685170 10.1038/nm1185

[26] Solinas, G. et al. JNK1 in hematopoietically derived cells contributes to diet-induced inflammation and insulin resistance without affecting obesity. *Cell Metab.* **6**, 386–397 (2007). 17983584 10.1016/j.cmet.2007.09.011

[27] Nguyen, M. T. A. et al. A subpopulation of macrophages infiltrates hypertrophic adipose tissue and is activated by free fatty acids via toll-like receptors 2 and 4 and JNK-dependent pathways. *J. Biol. Chem.* **282**, 35279–35292 (2007). 17916553 10.1074/jbc.M706762200

[28] Itani, S. I., Ruderman, N. B., Schmieder, F. & Boden, G. Lipid-induced insulin resistance in human muscle is associated with changes in diacylglycerol, protein kinase C, and IκB-α. *Diabetes* **51**, 2005–2011 (2002). 12086926 10.2337/diabetes.51.7.2005

[29] Heilbronn, L. & Campbell, L. Adipose tissue macrophages, low grade inflammation and insulin resistance in human obesity. *Curr. Pharm. Des.* **14**, 1225–1230 (2008). 18473870 10.2174/138161208784246153

[30] Weisberg, S. P. et al. Obesity is associated with macrophage accumulation in adipose tissue. *J. Clin. Invest.* **112**, 1796–1808 (2003). 14679176 10.1172/JCI19246 PMC296995

[31] Fuchs, A. et al. Associations among adipose tissue immunology, inflammation, exosomes and insulin sensitivity in people with obesity and nonalcoholic fatty liver disease. *Gastroenterology* **161**, 968–981 (2021). 34004161 10.1053/j.gastro.2021.05.008 PMC8900214

[32] Manowsky, J., Camargo, R. G., Kipp, A. P., Henkel, J. & Püschel, G. P. Insulin-induced cytokine production in macrophages causes insulin resistance in hepatocytes. *Am. J. Physiol. Endocrinol. Metab.* **310**, E938–E946 (2016). 27094035 10.1152/ajpendo.00427.2015

[33] Kim, H. et al. Differential effects of interleukin-6 and -10 on skeletal muscle and liver insulin action in vivo. *Diabetes* **53**, 1060–1067 (2004). 15047622 10.2337/diabetes.53.4.1060

[34] Lambertucci, F. et al. Disruption of tumor necrosis factor alpha receptor 1 signaling accelerates NAFLD progression in mice upon a high-fat diet. *J. Nutr. Biochem.* **58**, 17–27 (2018). 29860102 10.1016/j.jnutbio.2018.04.013

[35] Errafii, K., Boujraf, S. & Chikri, M. Transcriptomic analysis from normal glucose tolerance to T2D of obese individuals using bioinformatic tools. *Int. J. Mol. Sci.* **24**, 6337 (2023). 37047308 10.3390/ijms24076337 PMC10093815

[36] van den Hoek, A. M. et al. Unraveling the transcriptional dynamics of NASH pathogenesis affecting atherosclerosis. *Int. J. Mol. Sci.* **23**, 8229 (2022). 35897797 10.3390/ijms23158229 PMC9331250

[37] Nguyen‐Ngo, C., Willcox, J. C. & Lappas, M. Anti-diabetic, anti-inflammatory, and anti-oxidant effects of naringenin in an in vitro human model and an in vivo murine model of gestational diabetes mellitus. *Mol. Nutr. Food Res.* **63**, e1900224 (2019). 31343820 10.1002/mnfr.201900224

[38] Muzurović, E., Mikhailidis, D. P. & Mantzoros, C. Non-alcoholic fatty liver disease, insulin resistance, metabolic syndrome and their association with vascular risk. *Metabolism* **119**, 154770 (2021). 33864798 10.1016/j.metabol.2021.154770

[39] Nau, G. J. et al. Human macrophage activation programs induced by bacterial pathogens. *Proc. Natl Acad. Sci. USA* **99**, 1503–1508 (2002). 11805289 10.1073/pnas.022649799 PMC122220

[40] Takiguchi, H. et al. Macrophages with reduced expressions of classical M1 and M2 surface markers in human bronchoalveolar lavage fluid exhibit pro-inflammatory gene signatures. *Sci. Rep.* **11**, 8282 (2021). 33859282 10.1038/s41598-021-87720-y PMC8050093

[41] Shen, N. et al. An early response transcription factor, Egr-1, enhances insulin resistance in type 2 diabetes with chronic hyperinsulinism. *J. Biol. Chem.* **286**, 14508–14515 (2011). 21321112 10.1074/jbc.M110.190165 PMC3077649

[42] Wu, J. et al. Egr-1 transcriptionally activates protein phosphatase PTP1B to facilitate hyperinsulinemia-induced insulin resistance in the liver in type 2 diabetes. *FEBS Lett.* **593**, 3054–3063 (2019). 31309546 10.1002/1873-3468.13537

[43] Yan, Q. et al. The adipokine lipocalin 2 is regulated by obesity and promotes insulin resistance. *Diabetes* **56**, 2533–2540 (2007). 17639021 10.2337/db07-0007

[44] Xu, T. et al. Angptl7 promotes insulin resistance and type 2 diabetes mellitus by multiple mechanisms including SOCS3‐mediated IRS1 degradation. *FASEB J.* **34**, 13548–13560 (2020). 32786125 10.1096/fj.202000246RR

[45] Song, H., Huang, Q., Zhang, Y. & Shen, X. Wheat germ peptide improves glucose metabolism and insulin resistance in HepG2 hepatocytes via regulating SOCS3/IRS1/Akt pathway. *Nutr. Res.* **120**, 135–144 (2023). 38000279 10.1016/j.nutres.2023.10.005

[46] Chaudhary, L., Cheng, S. & Avioli, L. Induction of early growth response-1 gene by interleukin-1 beta and tumor necrosis factor- alpha in normal human bone marrow stromal and osteoblastic cells: regulation by a protein kinase C inhibitor. *Mol. Cell. Biochem.* **156**, 69–77 (1996). 8709978 10.1007/BF00239321

[47] Fitzgerald, K. A. & O’Neill, L. A. Characterization of CD44 induction by IL-1: a critical role for Egr-1. *J. Immunol.* **162**, 4920–4927 (1999). 10202038

[48] Zheng, C. et al. E2F1 induces tumor cell survival via nuclear factor-κB–dependent induction of EGR1 transcription in prostate cancer cells. *Cancer Res.* **69**, 2324–2331 (2009). 19276347 10.1158/0008-5472.CAN-08-4113

[49] Bongartz, H., Seiß, E. A., Bock, J. & Schaper, F. Glucocorticoids attenuate interleukin-6-induced c-Fos and Egr1 expression and impair neuritogenesis in PC12 cells. *J. Neurochem.* **157**, 532–549 (2021). 33454999 10.1111/jnc.15305

[50] Xu, A. et al. Lipocalin-2 is an inflammatory marker closely associated with obesity, insulin resistance, and hyperglycemia in humans. *Clin. Chem.* **53**, 34–41 (2007). 17040956 10.1373/clinchem.2006.075614

[51] Shi, M. et al. Estrogen receptor-regulated SOCS3 modulation via JAK2/STAT3 pathway is involved in BPF-induced M1 polarization of macrophages. *Toxicology* **433-434**, 152404 (2020). 32044397 10.1016/j.tox.2020.152404

[52] Kim, J. et al. EGR1-dependent PTEN upregulation by 2-benzoyloxycinnamaldehyde attenuates cell invasion and EMT in colon cancer. *Cancer Lett.* **349**, 35–44 (2014). 24704156 10.1016/j.canlet.2014.03.025

[53] Jorgensen, S. B. et al. Deletion of skeletal muscle SOCS3 prevents insulin resistance in obesity. *Diabetes* **62**, 56–64 (2013). 22961088 10.2337/db12-0443 PMC3526029

[54] Kim, K. E. et al. Lipocalin‐2 activates hepatic stellate cells and promotes nonalcoholic steatohepatitis in high‐fat diet–fed Ob/Ob mice. *Hepatology* **77**, 888–901 (2022). 10.1002/hep.32569 PMC9936980 35560370

[55] Jia, R. B. et al. Mitigation mechanisms of Hizikia fusifarme polysaccharide consumption on type 2 diabetes in rats. *Int. J. Biol. Macromol.* **164**, 2659–2670 (2020). 32846181 10.1016/j.ijbiomac.2020.08.154

[56] Zhang, J. et al. Dietary obesity-induced Egr-1 in adipocytes facilitates energy storage via suppression of FOXC2. *Sci. Rep.* **3**, 1476 (2013). 23502673 10.1038/srep01476 PMC3600596

[57] Bao, S., Li, F., Duan, L., Li, J. & Jiang, X. Thyroid-stimulating hormone may participate in insulin resistance by activating toll-like receptor 4 in liver tissues of subclinical hypothyroid rats. *Mol. Biol. Rep.* **50**, 10637–10650 (2023). 37884783 10.1007/s11033-023-08834-2

[58] Krenkel, O. & Tacke, F. Liver macrophages in tissue homeostasis and disease. *Nat. Rev. Immunol.* **17**, 306–321 (2017). 28317925 10.1038/nri.2017.11

[59] Kim, J. D., Yoon, N. A., Jin, S. & Diano, S. Microglial UCP2 mediates inflammation and obesity induced by high-fat feeding. *Cell Metab.* **30**, 952–962 (2019). 31495690 10.1016/j.cmet.2019.08.010 PMC7251564

[60] Rave, K. et al. Renal glucose excretion as a function of blood glucose concentration in subjects with type 2 diabetes-results of a hyperglycaemic glucose clamp study. *Nephrol. Dial. Transplant.* **21**, 2166–2171 (2006). 16627603 10.1093/ndt/gfl175

