# Supplementary material for: Thyrotropin exacerbates insulin resistance by triggering macrophage inflammation in subclinical hypothyroidism
Source: Exp Mol Med. 2025 Jun 16;57(6):1246–59. doi: 10.1038/s12276-025-01478-1 (PMC12229657; doi:10.1038/s12276-025-01478-1)
Supplement: Supplementary file 1 — Supplementary Information [file 12276_2025_1478_MOESM1_ESM.pdf]

# Supplementary Information

## MATERIALS AND METHODS

### Mice

Lysozyme 2 (Lyz2)-cre mice were purchased from the Jackson Laboratory (Stock No. 004781), which express the Cre recombinase transgene under the control of the *Lyz2* promoter/enhancer elements. *Tshr*$^{f/f}$ mice were generated under the C57BL/6N genetic background by Cyagen Co. Ltd, in which exon 10-termination codon of the *Tshr* allele was flanked by *loxP* sites. Myeloid cell-specific *Tshr* knockout mice (*Tshr*$^{MKO}$) were subsequently produced by intercrossing *Tshr*$^{f/f}$ mice with homozygous *Lyz2-Cre* mice. Mice were genotyped by PCR with DNA isolated from tails using the primers (Primer-1, 5'-CAC CTG CAC ATG AAA GCA TAT GTA A-3', Primer-2, 5'-GGT CAA CGT GGC AGA ATT AAA CT-3'). Genotyping of *Lyz2-Cre* was performed using specific primers (Primer-1, 5'-CTT GGG CTG CCA GAA TTT CTC-3', Primer-2, 5'-CCC AGA AAT GCC AGA TTA CG-3' and Primer-3, 5'-TTA CAG TCG GCC AGG CTG AC-3'). *Tshr*$^{MKO}$ mice were further verified for myeloid-conditional *Tshr* depletion in BMDMs by western blotting and immunofluorescence assays. *Tshr*$^{f/f}$ mice were used as wild-type (WT) control in this study.

All animal studies were supervised and approved by the Institutional Animal Ethics Committee of Xi'an Jiaotong University. Mice were housed in laboratory cages at 22–24 °C, 50–60% humidity under controlled conditions (12 h light/dark

cycle) with free access to food and water. The animals were housed with 5–6 mice per cage. Mice were maintained on a normal diet (P1200F, Jiangsu medicine). For diet-induced obesity, mice (with same genders) were fed on a high-fat diet (HFD, Fat content >60% Kcal, TP23300, Trophic) for 6 to 15 weeks old. Mice were monitored on a daily basis and weighted every week. Food intake and water drinking were tested by only one mouse in single cage every day for 13-14 weeks. At the end of the experiment (on HFD for 9 weeks), mice were euthanized via $CO_2$-dependent asphyxiation and their tissues were harvested.

**Clinical samples**

Peripheral blood samples of 26 SH patients and 26 healthy controls were obtained from The First Affiliated Hospital of Xi'an Jiaotong University. All patients did not receive any treatment interventions and signed an informed consent form. This study was approved by the Institutional Review Board and Human Ethics Committee of the First Affiliated Hospital of Xi'an Jiaotong University. Peripheral blood mononuclear cells (PBMCs) were isolated from heparinized blood by density gradient centrifugation (Ficoll density gradient medium, GE17-1440-02, Merck) according to standard protocols. Cells were cultured in RPMI-1640 medium with 100 units/mL penicillin, 100 μg/mL streptomycin (P/S, 03-031-5B, BI) and supplemented with 10% fetal bovine serum (FBS), at 37°C in a 5% $CO_2$ atmosphere. PBMCs were differentiated into immature macrophages (M0 macrophages) using 50 ng/mL of human macrophage colony-stimulating factor (hM-CSF, HY-P73827, MCE)

for 7 days, accompanied by stimulation with the serum from SH patients and health controls. The levels of CD80 were examined using flow cytometry.

**Cell culture and stimulation**

To isolate primary mouse bone marrow derived macrophages (BMDMs), 6 to 8-week-old mice were euthanized. Briefly, their femurs and tibias were collected in sterile conditions, and all the tissues were removed from the bones. Each end of bone was cut off and the bone marrow was flushed out using a 1 mL syringe filled with Dulbecco's Modified Eagle's Medium (DMEM, MI00622, MISHU). After centrifuging at 300 × g for 5 min, the pellets were collected and dissociated in lysis buffer to lyse red blood cells. A single cell suspension was prepared by passing the cells through a 40 μm nylon cell strainer (352340, Falcon). Bone marrow cells were cultured and differentiated for 7 days in DMEM containing 10% FBS and 1% penicillin/streptomycin, supplemented with murine recombinant M-CSF (10ng/mL, 315-02-50UG, Gibco) at 37 °C with 5% $CO_2$. The efficiency of *Tshr* knockdown was verified by western blotting and immunofluorescence assays. BMDMs were stimulated by 1ng/mL TSH (8885-TH-010, R&D Systems) for 24 h. The levels of CD80 and ROS was examined using flow cytometry. The effect of TSH on the phosphorylation of p65 in BMDMs was detected by western blotting analysis. The mRNA sequencing was used to determine the change of proinflammatory cytokines in TSH-stimulated BMDMs. We also performed qRT-PCR assay to confirm the

mRNA changes of *Il-1a* , *Il-1b* and *Il-6* in BMDMs stimulated by 1 ng/mL TSH or PBS for 24 h.

Primary hepatocytes were isolated according to a previous method[61]. Briefly, Buffer A (Calcium and magnesium-free PBS containing 0.2 mM EGTA, 10 mM HEPES, 5 mM KCl, 1 mM glucose and 0.2% BSA, all from Aladdin) and Buffer B (PBS with 1 mM magnesium and 1 mM calcium, 0.2% BSA, 30 mM HEPE and 5 mM KCl) containing collagenase (100 IU/ml, 17104019, Gibco) were aliquoted into 50 mL tubes and kept warm at 40℃ in a water bath. Mice were anaesthetized by intramuscular injection of a ketamine-xylazine-acepromazine cocktail (KXA). Hepatocytes were prepared by collagenase digestion via catheterization of the inferior vena cava (IVC) using a 24G needle catheter. Prior to collagenase infusion, the liver was perfused (3-4 min) with Buffer A via IVC at a speed of 5 mL per min after severing the portal vein. The color of the liver was observed, which changed to a beige or light brown color. Next, perfusion was continued with collagenase in Buffer B for 2 min. Within 2 min of digestion, the liver was monitored for the appearance of cracking on the surface. Perfusion was stopped immediately and the liver was excised out into ice-chilled dispersion buffer (DMEM, 10% FBS, 1% penicillin and streptomycin, 0.5 μg/mL insulin (I189675, Aladdin), 100 nM dexamethasone (D8040, Solarbio), 15 mM HEPES). Cells from the digested livers were teased out, suspended in dispersion buffer, filtered through 70 μm nylon filter and centrifuged at 50 × g for 1 min at 4℃. The precipitation was hepatocytes, while the supernatant was liver nonparenchymal cells (including macrophages, hepatic

stellate cells and endotheliocytes), which were subsequently used for flow cytometry analysis. The cell pellet in the precipitation was washed once in dispersion buffer by resuspending and centrifuging at 50×g for 1 min at 4 ℃. The cell pellet was then mixed with 15 mL Percoll (P8370, Solarbio, adjusted to physiological ionic strength with 10 × PBS) to a final concentration of 40% and centrifuged at 100 × g for 10 min at 4 ℃. Hepatocytes were collected as a pellet and washed once with dispersion buffer and then cultured on collagen (C8062， Solarbio)-coated plates (200000 cells per well in 12-well plate) in DMEM containing 1% penicillin and streptomycin, 100 nM dexamethasone and 10% FBS.

3T3L1 preadipocytes (CL-0006, Procell Life Science & Technology Co. Ltd, Wuhan, China) were maintained in DMEM (MI00622, MISHU) containing 10% FBS and 1% P/S and kept at 37 °C and 5% $CO_2$. To induce preadipocyte differentiation, at 48 h post-confluence (day 0), cells were cultured in differentiation medium containing 10% FBS, 0.5 mM 3-isobutyl-1methylxanthine (IBMX, I106812, Aladdin), 1 μM dexamethasone and 1 μg/mL insulin until day 2. Cells were then cultured with DMEM supplemented with 10% FBS and 1 μg/mL insulin for 2 days, after which they were cultured with DMEM containing 10% FBS. L6 rat skeletal myoblasts cells (CL-0136, Procell Life Science & Technology Co. Ltd, Wuhan, China) were maintained in DMEM containing 10% FBS and 1% P/S and kept at 37 °C and 5% $CO_2$. After cultures reached 80%-90% confluency, the medium was switched to 2% FBS to allow differentiation and myotube formation. After 8-10 days, cultures were used for experiments. HepG2 cells (ATCC, #HB-8065) were

maintained in DMEM containing 10% FBS and 1% P/S and kept at 37 °C and 5% $CO_2$.

**Determination of fat and lean mass**

Fat and lean mass of mice (male, HFD-fed for 8 weeks) were determined using the EchoMRI™ (EchoMRI™ Medical Systems).

**Fasting plasma insulin concentration**

Fasting plasma insulin concentration was measured using mouse insulin ELISA kit (E-EL-M1382c, Elabscience) after 8 h fasting.

**Glucose tolerance test (GTT)**

Mice were intraperitoneal injection of glucose (1.5 g/kg, D432808, Aladdin) and blood glucose levels were measured at 0, 15, 30, 60 and 120 min after injection after 16 h fasting.

**Insulin tolerance test (ITT)**

Mice were injected intraperitoneally with insulin (1.5 U/kg weight of mice) for 8 h fasting, and blood glucose levels were then measured at 0, 15, 30, 60 and 120 min after injection.

**Intra-hepatic triglyceride content**

Liver tissues (100 mg) were homogenized in 900 μL HPLC-grade ethanol. After homogenized at 4℃, the samples were centrifuged at 4℃, 10000 ×g for 10 min. The supernatant was measured enzymatically using kit (E-BC-K261-M, Elabscience). Hepatic triglyceride content was defined as mmol of triglyceride per gram of total liver proteins.

**Serum total cholesterol, ALT and AST content**

Kits for total cholesterol (TC, E-BC-K109-M, Elabscience), ALT (C009-2-1, jjcbio) and AST (C010-2-1, jjcbio) were used to measure their serum levels of mice after 8 h fasting.

**Measurement of triiodothyronine (T3), free thyroxine (FT4) and TSH**

T3 kit (mouse triiodothyronine ELISA Kit, JL13028, Joln), FT4 kit (Mouse free tetraiodothyronine ELISA kit, F2576, Fankew) and TSH kit (mouse thyroid stimulating hormone ELISA Kit, JL20301, Joln) were used to measure their levels in liver, adipose, skeletal muscle and serum of mice.

**Measurement of cytokines**

The levels of IL-1α, IL-1β, IL-6 and TNF in liver, adipose, skeletal muscle and serum of mice were measured by ELISA.

**Western blotting analysis**

Mice were fasted for 8 h, intraperitoneally injected with insulin (2 U/kg) and euthanized 5 min later. Liver, adipose, skeletal muscle, brain, testis and ovary tissues (5 mg in aliquots) were collected to prepare total protein lysates by liquid nitrogen grinding method. Cells were lysed in prechilled RIPA buffer (WB3100, NCM) supplemented with protease and phosphatase inhibitors (P003, NCM) and 0.2 mM PMSF (PL012, ZHHC). Proteins were separated by SDS-PAGE and transferred electrophoretically to polyvinylidene fluoride (PVDF) membranes (IPVH00010, Millipore). After blocking with 5% BSA (A1933, Sigma-Aldrich) in TBST (pH 7.4, 20 mM Tris-HCl, 15 m NaCl and 0.1% [vol/vol] Tween-20), the membranes were incubated overnight at 4°C with primary antibodies, which were described in Table S3. After washing with TBST, the membranes were incubated with the appropriate HRP-conjugated secondary antibody at 25℃ for 1.5 h. The bands were visualized by FluorChemQ system using a chemiluminescence kit (MI00607, MISHU). The images of western blotting were obtained using Image Lab software version 6.0. ImageJ version 1.52 (National Institutes of Health) was used for band densitometry analysis.

**Histologic analysis**

Livers, adipose tissues or skeletal muscle were dissected into sections (4 μm in thickness), after fixed in 4% PFA (P0099, Beyotime Biotechnology) for at least 24 h, and embedded in paraffin. Paraffin tissue sections were stained with hematoxylin and eosin (H&E, according to the manufacturer's instructions, G1004 and G1001,

Servicebio) or analyzed by immunofluorescence assay. Frozen sections (8 μm in thickness) were prepared for oil red staining (according to the manufacturer's instructions, 1320-06-5, Sigma-aldrich). BMDMs were seeded and allowed to be adhered on a coverslip for 24 h. Coverslip were then wash twice with PBS, fixed 15 min with methanol and permeated 10 min with PBS containing 0.3% Triton X-100 (P0096, Beyotime). For immunofluorescence experiments, after the paraffin was removed and antigen was unmasked, tissue sections or BMDMs-adhered coverslips were blocked with the goat serum (ZLI-9056, ZSGB-BIO) for 30 min and then incubated with the following primary antibodies: anti-TSHR (53542, Santa,1:50), anti-GLUT4 (347063, Zenbio,1:100), anti-p-p65 (3033, CST, 1:100), anti-CD11b (2488586, invitrogen,1:200), anti-F4/80 (28463-1-AP, proteintech,1:200) and anti-CD86 (NBP2-25208, NOVUS,1:100). After washing with PBS, tissue sections or BMDMs-adhered coverslips were incubated with the following secondary antibodies purchased from Thermo Fisher Scientific: Alexa Fluor 488 goat anti-mouse IgG (A32723, 1:2000), Alexa Fluor 488 Goat anti-rabbit IgG (A32731, 1:2000) and Alexa Fluor 647 goat anti-rat IgG (A48265, 1:2000). DAPI (C1002, Beyotime, 1:5000) was used to stain cell nuclei. Images were then captured using a fluorescence microscope (Leica) and analyzed by ImageJ (National Institutes of Health) version 1.52.

**Flow cytometry analysis of macrophages**

Mouse livers were perfused and digested with collagenase, followed by centrifuged at 50×g for 1 min at 4°C to obtain the supernatant. Next, the supernatant was centrifuged at 400×g for 7 min at 4°C, washed twice with PBS and resuspended with PBS. Skeletal muscle tissue was mechanically chopped and digested in HBSS supplemented with 1.5% bovine serum albumin (BSA), 2 mg/mL collagenase type II (17101015, ThermoFisher Scientific) and 2 mg/mL dispase II (4942078001, Roche) for 1 h at 37 °C. Cell suspension was filtered in sequence through 100μm and 40μm cell filters and harvested by centrifugation at 500 g for 10 min. Visceral adipose tissues (VATs) were mechanically chopped and then digested with collagenase II (17101015, ThermoFisher Scientific) for 15 min at 37 °C. After passing cells through a 100 μm cell strainer and centrifugation at 1000 g for 10 min, primary adipocytes gathering in the top layer of the supernatant were throwed away gently and the pellet containing macrophages was harvested. Differentiated macrophages from BMDMs were stimulated by 1 ng/mL TSH for 24 h before harvested. PBMCs derived macrophages were stimulated by the serum from SH patients and health controls for 7 days before harvested. To block Fc receptors, cells were pre-incubated with mouse TruStain FcX™ PLUS or human TruStain FcX™ (156603 or 422301 BioLegend; 0.25 μg per $10^6$ cells in a volume of 100 μL) for 5-10 min on ice. Cells were then stained with the following fluorescent-conjugated antibodies for 30 min at 4 °C in the dark: APC-anti-mouse-CD45 (147707, BioLegend, 0.25 μg/test), FITC-anti-human/mouse-CD11b (101205, BioLegend, 0.25 μg/test), PE-anti-mouse-F4/80 (111603, BioLegend, 0.25 μg/test) and

PE-Cyanine7-anti-mouse CD80 (104711, BioLegend, 0.5 μg/test), PE-anti-human-CD68 (333807, BioLegend, 0.25 μg/test), PE-Cyanine7-anti-human CD80 (305217, BioLegend, 0.25 μg/test). To exclude dead cells, 7-AAD (420403, BioLegend,1 μL/test) was added in the dark for 5-10 min before flow cytometry analysis. The mouse macrophages were defined as $CD45^{+}CD11b^{+}F4/80^{+}$ and further identified as $CD80^{+}$ M1 subsets. The human macrophages were defined as $CD11b^{+}CD68^{+}$ and further identified as $CD80^{+}$ M1 subsets. To measure ROS levels, differentiated BMDMs cells stimulated by 1 ng/mL TSH for 24 h in 6-well-plates were incubated in 1 mL DMEM with 1 μM DCFH-DA (D6883, Sigma Aldrich) for 1 h washed 3 times with PBS and detected in FITC channel. Samples were then subjected to flow cytometry analysis using Beckman coulter cytoflex. Data were analyzed with CytExpert (Version 2.5).

**RNA isolation and quantitative reverse transcription PCR (qRT-PCR)**

RNA was isolated from tissue (10 mg) by liquid nitrogen grinding method and from cells using the Trizol reagent (15596018, ThermoFisher Sicentific). cDNA was generated from 1 μg RNA using reverse transcription reagent Kit (RR047A, TaKaRa), and qRT-PCR assays were performed with SYBR green master mix (FS-Q1002, ForeverStar) using a QuantStudio Real-Time PCR System according to the manufacturer's instructions. Relative mRNA levels of the indicated genes were normalized to *β-actin* as an internal control. The primer sequences were presented in Table S4.

**Co-culture of primary hepatocytes, 3T3L1 adipocytes and L6 skeletal muscle cells with *Tshr*$^{f/f}$- and *Tshr*$^{MKO}$- derived BMDMs**

BMDMs (0.1 ×$10^6$/well) from *Tshr*$^{f/f}$ and *Tshr*$^{MKO}$ mice were co-cultured with primary hepatocytes, 3T3L1 adipocytes and L6 skeletal muscle cells at a ratio of 1:1 in transwells plate (0.4 μm polycarbonate filter, 3401, Corning), which were treated with IL-1RA (10 ng/mL, HY-P72566, MCE), IL-6ST (10 ng/mL, HY-P76370, MCE) or PBS for 48 h. BMDMs were plated in the upper chamber of transwells dish while insulin target cells were in the lower chamber. The upper chamber was then removed and the cells in the lower chamber were stimulated with insulin (100 nM) or PBS for 15 min before extracting proteins or RNAs.

**Measurement of PEPCK1 activity**

Primary hepatocytes were co-cultivated with *Tshr*$^{f/f}$- and *Tshr*$^{MKO}$-derived BMDMs for 48 h, and PEPCK1 activity assay kit (SH0441, Warbio) was used to measure PEPCK1 activity in hepatocytes. Briefly, the acid extract lysed cells (5×$10^6$ cells in 1 mL) by ultrasonic fracturing. The lysates were then centrifugated at 8000×g for 10 min at 4℃. Next, 50 μL supernatant, 50 μL Reagent 4 and 900 μL working liquid were mixed promptly. The initial absorbance values A1 and A2 after 1 min respectively were measured at 340 nm and 37 ℃. PEPCK activity was then calculated using the following formula: nmol/min/$10^4$ cell = 6.43×(A1-A2).

**Glucose uptake assay**

BMDMs from *Tshr*$^{f/f}$ and *Tshr*$^{MKO}$ mice were differentiated into macrophages, and continued to be cultivated for 48 h in fresh DMEM supplemented with 10% FBS. The medium was collected and centrifuged at 300×g for 10 min. The supernatant was then filtered through 40 μm nylon filter as conditioned media (CM). Next, 3T3L1 adipocytes and L6 myocytes were respectively plated at 20,000 or 5000 cells per 100 μL in a 96-well plate. Differentiation was induced by the method mentioned above. Cells were treated with CM for 48 h, followed by glucose uptake assay (J1341, Promega).

**mRNA sequencing and data analysis**

RNeasy Micro Kit (QIAGEN) was used to isolate RNA from 10 mg liver tissues of euthanized mice (male *Tshr*$^{MKO}$ and *Tshr*$^{f/f}$ littermates, for 9 weeks of HDF-fed, *n* =3 per group) stimulated by insulin (1.5 U/kg weight of mice) for 5 min after 8 h fasting. RNA was similarly isolated from BMDMs from mice (male *Tshr*$^{MKO}$ and *Tshr*$^{f/f}$ littermates, *n* =3 per group) stimulated by 1 ng/mL TSH for 24 h using RNeasy Micro Kit (QIAGEN). Quantity and quality of each mRNA sample were examined using gel electrophoresis and with Qubit (Thermo, Waltham, MA, USA). Strand-specific libraries were constructed using the TruSeq RNA sample preparation kit (Illumina, San Diego, CA, USA), and the sequencing was then carried out using the Illumina Novaseq 6000 instrument by the commercial service of Genergy Biotechnology Co. Ltd. (Shanghai, China).

The raw data were handled by Skewer and data quality was checked by FastQC v0.11.2. Differentially expression genes (DEGs) were determined using the MA-plot-based method with Random Sampling (MARS) model in the DEGseq package between different groups. Generally, in MARS model, M = log2C1 - log2C2, and A = (log2C1 + log2C2)/2 (C1 and C2 denote the counts of reads mapped to a specific gene obtained from two samples). The thresholds for determining DEGs are $P < 0.05$ and absolute fold change ≥2. DEGs were then chosen for function and signaling pathway enrichment analysis using GO and KEGG database. We use the local version of the Gene Set Enrichment Analysis (GSEA) tool. The significantly enriched pathways were determined when $P < 0.05$ and at least two affiliated genes were included.

**RNA interference (RNAi)-mediated downregulation of *EGR1***

Control siRNA (si-NC) and siRNAs targeting *EGR1* (si-EGR1 #2 and si-EGR1 #3) were obtained from Tsingke Biotechnology (Beijing, China). HepG2 cells were transfected at 45-50% confluence using X-tremeGENE siRNA transfection reagent (4476093001, Roche) according to the instructions of the manufacturer. Meanwhile, cells were treated with PBS or IL-1α for 48 h, followed by 100 nM insulin stimulation for 15 min. siRNA sequences were presented in Table S5.

**Ectopic expression of *EGR1***

*EGR1*-Flag-overexpressing plasmid (P23788, Miaoling Biology) or empty vector were transfected into HepG2 cells for 48 h using X-tremeGENE™ DNA transfection reagent (6365779001, Roche) according to the instructions of the manufacturer.

**Dual-luciferase reporter assay**

JASPAR (http://jaspardev.genereg.net/) was used to analyze the potential binding sites of transcription factor *EGR1* in the promoters of *LCN2* and *SOCS3*. Next, the promoter regions of *LCN2* and *SOCS3* were amplified from human genomic DNA and cloned into the pGL3.0-basic luciferase reporter vector. The primer sequences were presented in Table S6. Then, 100 nM si-EGR1 or 1000 ng EGR1-Flag-overexpressing plasmid, 1000 ng reporter vector and 200 ng pRL-TK plasmid were co-transfected into HepG2 cells in duplicate 24-well plates using Lipofectamine 3000 (L3000015, Invitrogen) according to the manufacturer's instructions. Cells were harvested to analyze the relative luciferase activity using dual-luciferase reporter system (E1910, Promega).

**Chromatin immunoprecipitation (ChIP) assay**

ChIP assays were performed using a simple ChIP enzymatic chromatin IP kit (9003, CST). *EGR1*-Flag overexpressing HepG2 cells or control cells were cross linked with 1% formaldehyde at room temperature for 10 min, and glycine solution was then added to stop the cross-linking reaction. Fragmented chromatin was treated with nuclease and subjected to sonication. Chromatin immunoprecipitation was

performed overnight at 4 °C with mouse anti-Flag antibody (1:50, HA601167, HUABIO). Anti-acetylated histone H3 (1:250) was used as a positive control, and normal rabbit IgG (1:250) was used as a negative control. Protein G Magnetic Beads were added for another 24 h at 4 °C. The chromatins were then washed and eluted from the protein G magnetic beads using buffers supplied with the kit and DNA was analyzed by conventional or real-time PCR. The primers used to amplify different promoter regions of *LCN2* or *SOCS3* containing EGR1 binding sites were also presented in Table S6.

**Statistical analysis**

All data were analyzed using GraphPad Prism 9. Statistical significance between two groups was determined using unpaired two tailed Student's *t* test or 1-way ANOVA with Tukey's multiple-comparison test or two-way analysis of variance (ANOVA) with Sidak's multiple comparisons test. Linear correlation analysis with Pearson. Data are presented as mean ± standard error (SE) or standard deviation (SD). *P* values <0.05 were considered statistically significant.

# References

61. Li, P. et al. Hematopoietic-Derived Galectin-3 Causes Cellular and Systemic Insulin Resistance. *cell*. **167**, 973-984 (2016).

# Supplementary Tables

Supplementary **Table 1. The mRNA expression of cytokine receptors in liver tissues**

| **Cytokines** | **WT1** | **WT2** | **WT3** | **MKO1** | **MKO2** | **MKO3** |
|---|---|---|---|---|---|---|
| *Il12rb2* | 4 | 1 | 31 | 0 | 27 | 2 |
| *Il12rb1* | 49 | 19 | 85 | 46 | 57 | 51 |
| *Il6ra* | 3253 | 3656 | 4969 | 3926 | 6255 | 3407 |
| *Il1r2* | 15 | 2 | 7 | 11 | 20 | 11 |
| *Il1r1* | 7097 | 3184 | 4761 | 4888 | 6455 | 3212 |
| *Cxcr2* | 36 | 9 | 36 | 20 | 35 | 24 |
| *Cxcr3* | 3 | 0 | 7 | 3 | 17 | 20 |
| *Il23r* | 0 | 0 | 0 | 0 | 0 | 0 |
| *Tnfrsf1b* | 1448 | 2189 | 1992 | 1794 | 2806 | 2099 |
| *Tnfrsf1a* | 2446 | 2206 | 2698 | 3298 | 2392 | 2030 |
| *Ccr3* | 0 | 0 | 10 | 5 | 5 | 2 |
| *Ccr2* | 32 | 27 | 31 | 86 | 101 | 35 |

Supplementary **Table 2**. Chemical and biological reagents used in this study

| Chemicals | Company | Cat # | Application |
|---|---|---|---|
| High-fat diet (HFD) | Trophic | TP23300 | Rodent diet |
| Normal diet | Jiangsu medicine | P1200F | Rodent diet |
| Insulin | aladdin | I189675 | Insulin tolerance test |
| Glucose | aladdin | D432808 | Glucose tolerance test |
| DMEM | MISHU | MI00622 | Cell culture |
| FBS | YOSHI | A1015 | Cell culture |
| Penicillin/streptomycin | BI | 03-031-5B | Cell culture |
| Recombinant mouse M-CSF protein | Gibco | 315-02-50UG | Cell culture |
| Recombinant mouse TSH α / β Heterodimer protein | R&D Systems | 8885-TH-010 | Cell culture |
| EGTA | aladdin | 67-42-5 | Primary hepatocytes isolation |
| HEPES | aladdin | H109408 | Primary hepatocytes isolation |
| KCl | aladdin | P301833 | Primary hepatocytes isolation |
| BSA (Bovine serum albumin) | Sigma-Aldrich | A1933 | Primary hepatocytes isolation and immunoblot |
| Collagenase Type IV | Gibco | 17104019 | Liver tissue digestion |
| Dexamethasone | Solarbio | D8040 | Cell induction |
| Percoll | Solarbio | P8370 | Primary hepatocytes isolation |
| Collagen | Solarbio | C8062 | Primary hepatocytes culture |
| Collagenase Type II | Gibco | 17101015 | Adipose and skeletal muscle digestion |
| Dispase II | Roche | 4942078001 | skeletal muscle digestion |
| 3T3L1 preadipocytes | Procell Life Science & Technology | CL-0006 | Cell culture |
| L6skeletal myoblasts cells | Procell Life Science & Technology | CL-0136 | Cell culture |
| 3-isobutyl-1methylxanthine (IBMX) | Aladdin | I106812 | Cell induction |
| Mouse insulin ELISA kit | Elabscience | E-EL-M1382c | Insulin analysis |
| Triglyceride (TG) colorimetric assay kit | Elabscience | E-BC-K261-M | TG analysis |
| Total cholesterol (TC) colorimetric assay kit | Elabscience | E-BC-K109-M | TC analysis |
| ALT Assay Kit | jjcbio | C009-2-1 | ALT activity |
| AST Assay Kit | jjcbio | C010-2-1 | AST activity |

| | | | |
|---|---|---|---|
| Mouse thyroid stimulating hormone(TSH) ELISA kit | Joln | JL20301 | TSH levels |
| Mouse triiodothyronine (T3) ELISA kit | Joln | JL13028 | T3 levels |
| Mouse free tetraiodothyronine (FT4) ELISA kit | Fankew | F2576 | FT4 levels |
| RIPA(Radio Immunoprecipitation Assay) | NCM | WB3100 | Lysate of cell and tissue |
| Phosphatase inhibitors | NCM | P003 | Lysate of cell and tissue |
| PMSF(Phenylmethanesulfonyl fluoride) | ZHHC | PL012 | Lysate of cell and tissue |
| Polyvinylidene fluoride membranes (PVDF) | Millipore | IPVH00010 | Immunoblot |
| Chemiluminescence kit | MISHU | MI00607 | Immunoblot |
| PFA | Beyotime | P0099 | Fixing tissue |
| Hematoxylin | Servicebio | G1004 | H&E staining |
| Eosin | Servicebio | G1001 | H&E staining |
| Oil red O solution | Sigma-aldrich | 1320-06-5 | Oil red staining |
| Triton X-100 | Beyotime | P0096 | Cell permeation of immunofluorescence |
| Goat serum | ZSGB-BIO | ZLI-9056 | Tissues blocking of immunofluorescence |
| DAPI | Beyotime | C1002 | Staining cell nuclei of IF |
| 7-AAD | BioLegend | 420403 | FACS |
| DCFH-DA | Sigma Aldrich | D6883 | FACS |
| Trizol reagent | ThermoFisher Sicentific | 15596018 | RNA extraction |
| Reverse transcription reagent Kit | TaKaRa | RR047A | RNA reverse transcription |
| SYBR green master mix | ForeverStar | FS-Q1002 | qRT-PCR assay |
| Mouse IL-1α ELISA kit | Fankew | F2168 | IL-1α levels |
| Mouse IL-1β ELISA kit | Fankew | F2040 | IL-1β levels |
| Mouse IL-6 ELISA kit | Fankew | F2163 | IL-6 levels |
| Mouse TNF ELISA kit | Fankew | F2132 | TNF levels |
| Recombinant mouse IL-1RA protein | MCE | HY-P72566 | Cell culture |
| Recombinant mouse IL-6ST protein | MCE | HY-P76370 | Cell culture |
| Recombinant human IL-1α protein | MCE | HY-P7027 | Cell culture |
| Pepck activity Assay | Warbio | SH0441 | Pepck activity |
| Glucose uptake assay | Promega | J1341 | Glucose uptake analysis |
| DMEM without serum or glucose | Life Technologies | 11966 | Cell culture |

| | | | |
|---|---|---|---|
| X-tremeGENE siRNA transfection reagent | Roche | 4476093001 | siRNA transfection |
| EGR1-Flag-overexpressing plasmid | Miaoling Biology | P23788 | Overexpressing EGR1 |
| X-tremeGENE™ DNA transfection reagent | Roche | 6365779001 | DNA transfection |
| Lipofectamine 3000 | Invitrogen | L3000015 | DNA transfection |
| Dual-luciferase reporter assay | Promega | E1910 | Luciferase activity |
| Simple ChIP enzymatic chromatin IP kit | CST | 9003 | ChIP |
| Human IL-1α ELISA kit | Fankew | F0056 | IL-1αlevels |
| Human IL-1β ELISA kit | Fankew | F0179 | IL-1βlevels |
| Human IL-6 ELISA kit | Fankew | F0049 | IL-6 levels |
| Human GHb ELISA kit | Fankew | F10587 | GHb levels |
| Ficoll density gradient medium | Merck | GE17-1440-02 | PBMC isolation |
| Human M-CSF protein | MCE | HY-P73827 | PBMC induction |

Supplementary **Table 3**. The antibodies used in this study

| **Antibodies** | **Company** | **Cat #** | **Application** |
|---|---|---|---|
| Rabbit PEPCK1 (D12F5) | CST | 12940 | Western |
| Rabbit phosphorylated GSK 3 β (Ser9) (D85E12) | CST | 5558 | Western |
| Rabbit GSK-3 β (27C10) | CST | 9315 | Western |
| Rabbit PPAR γ | abcam | ab310323 | Western |
| Mouse GCK (G-6) | Santa | 17819 | Western |
| Rabbit phosphorylated Akt (Ser473) (D9E) | CST | 4060 | Western |
| Rabbit Akt (pan) (C67E7) | CST | 4691 | Western |
| Rabbit phosphorylated PDK1 (Ser241) | CST | 3061 | Western |
| Rabbit PDK1 | CST | 3062 | Western |
| Rabbit phosphorylated IRS1 (Ser636) | Zenbio | 251484 | Western |
| Rabbit IRS1 | Zenbio | R381383 | Western |
| Rabbit β-Actin | Abways | AB0033 | Western |
| Rabbit glucose Transporter (GLUT4) | Zenbio | 347063 | IF |
| Mouse TSHR(3B12) | Santa | 53542 | Western, IF |

| | | | |
|---|---|---|---|
| Rabbit Thyroid Hormone Receptor alpha(THR α ) | Zenbio | R389157 | Western |
| Rabbit Hsp90 alpha/beta | Zenbio | R24635 | Western |
| Rabbit phosphorylated Stat3 (Tyr705) (D3A7) | CST | 9145 | Western |
| Rabbit Stat3 (79D7) | CST | 4904 | Western |
| Rabbit phosphorylated NF- κ B p65 (Ser536) (93H1) | CST | 3033 | Western, IF |
| Rabbit NF-KB p65 | Zenbio | 380172 | Western |
| Rabbit phosphorylated JNK (Thr183/Tyr185) | Zenbio | 340810 | Western |
| Rabbit JNK1 | Zenbio | R24778 | Western |
| Rabbit EGR1 | Zenbio | R30051 | Western |
| Rabbit SOCS3 | Zenbio | 500694 | Western |
| Rabbit Lipocalin 2 | Zenbio | R381715 | Western |
| Rabbit PTEN (138G6) | CST | 9559 | Western |
| Rat CD11b | invitrogen | 2488586 | IF |
| Rabbit F4/80 | proteintech | 28463-1-AP | IF |
| Mouse CD86 | NOVUS | NBP2-25208 | IF |
| TruStain FcX™ PLUS (Fc block) | BioLegend | 156603 | FACS |
| APC-anti-mouse-CD45 | BioLegend | 147707 | FACS |
| FITC-anti-human/mouse-CD11b | BioLegend | 101205 | FACS |
| PE-anti-mouse-F4/80 | BioLegend | 111603 | FACS |
| PE-CY7-anti-mouse CD80 | BioLegend | 104711 | FACS |
| PE-CY7-Hamster IgG isotype control | BioLegend | 400921 | FACS, isotype control |
| Mouse anti-FLAG antibody | HUABIO | HA601167 | ChIP |
| Human TruStain FcX™ (Fc block) | BioLegend | 422301 | FACS |
| PE-anti-human-CD68 | BioLegend | 333807 | FACS |
| PE-CY7-anti-human CD80 | BioLegend | 305217 | FACS |

Supplementary **Table 4**. The primers used in this study for qRT-PCR

| Genes | Forward primer (5'-3') | Reverse primer (5'-3') |
|---|---|---|
| *mTshr* | GTCACTGCCCTTCCTTCCAA | CCTCAAGATGTTCACTGATTTCCT |
| *mItgam* | TACTTCGGGCAGTCTCTGAGTG | ATGGTTGCCTCCAGTCTCAGCA |
| *mAdgre1* | CGTGTTGTTGGTGGCACTGTGA | CCACATCAGTGTTCCAGGAGAC |
| *mItgax* | TGCCAGGATGACCTTAGTGTCG | CAGAGTGACTGTGGTTCCGTAG |
| *mIl12a* | ACGAGAGTTGCCTGGCTACTAG | CCTCATAGATGCTACCAAGGCAC |
| *mIl6* | TACCACTTCACAAGTCGGAGGC | CTGCAAGTGCATCATCGTTGTTC |
| *mIl12b* | TTGAACTGGCGTTGGAAGCACG | CCACCTGTGAGTTCTTCAAAGGC |
| *mIl1b* | TGGACCTTCCAGGATGAGGACA | GTTCATCTCGGAGCCTGTAGTG |
| *mCxcl3* | TGAGACCATCCAGAGCTTGACG | CCTTGGGGGTTGAGGCAAACTT |
| *mCxcl1* | TCCAGAGCTTGAAGGTGTTGCC | AACCAAGGGAGCTTCAGGGTCA |
| *mIl1a* | ACGGCTGAGTTTCAGTGAGACC | CACTCTGGTAGGTGTAAGGTGC |
| *mIl23a* | CATGCTAGCCTGGAACGCACAT | ACTGGCTGTTGTCCTTGAGTCC |
| *mCxcl10* | ATCATCCCTGCGAGCCTATCCT | GACCTTTTTTGGCTAAACGCTTTC |
| *mCxcl2* | GAAGTCATAGCCACTCTCAAGG | CCTCCTTTCCAGGTCAGTTAGC |
| *mTnf* | GGTGCCTATGTCTCAGCCTCTT | GCCATAGAACTGATGAGAGGGAG |
| *mCxcl11* | CCGAGTAACGGCTGCGACAAAG | CCTGCATTATGAGGCGAGCTTG |
| *mCcl5* | CCTGCTGCTTTGCCTACCTCTC | ACACACTTGGCGGTTCCTTCGA |
| *mCxcl9* | CCTAGTGATAAGGAATGCACGATG | CTAGGCAGGTTTGATCTCCGTTC |
| *mCcl2* | AGGTCCCTGTCATGCTTCTG | GCTGCTGGTGATCCTCTTGT |
| *mEgr1* | AGCGAACAACCCTATGAGCACC | ATGGGAGGCAACCGAGTCGTTT |
| *mLcn2* | ATGTCACCTCCATCCTGGTCAG | GCCACTTGCACATTGTAGCTCTG |
| *mSocs3* | GGACCAAGAACCTACGCATCCA | CACCAGCTTGAGTACACAGTCG |
| *mPten* | TGAGTTCCCTCAGCCATTGCCT | GAGGTTTCCTCTGGTCCTGGTA |
| *mActb* | AACAGTCCGCCTAGAAGCAC | CGTTGACATCCGTAAAGACC |
| *hEGR1* | AGCAGCACCTTCAACCCTCAGG | GAGTGGTTTGGCTGGGGTAACT |
| *hLCN2* | GTGAGCACCAACTACAACCAGC | GTTCCGAAGTCAGCTCCTTGGT |

| | | |
|---|---|---|
| *hSOCS3* | CATCTCTGTCGGAAGACCGTCA | GCATCGTACTGGTCCAGGAACT |
| *hPTEN* | TGAGTTCCCTCAGCCGTTACCT | GAGGTTTCCTCTGGTCCTGGTA |
| *hACTB* | CACCATTGGCAATGAGCGGTTC | AGGTCTTTGCGGATGTCCACGT |

Supplementary **Table 5**. Oligonucleotide sequences of siRNAs targeting *EGR1*

| **Gene** | **Sense (5'-3')** | **Antisense (5'-3')** |
|---|---|---|
| si-EGR1#1 | CAGUAUCAUCUCCAUCAUA | UAUGAUGGAGAUGAUACUG |
| si-EGR1#2 | CAAUUACUAUUCCCUUUGA | UCAAAGGGAAUAGUAAUUGGG |
| si-EGR1#3 | UCUCCCAGGACAAUUGAAAUUU GCU | AGCAAAUUUCAAUUGUCCUGGGA GA |

Supplementary **Table 6**. The primers used in this study for dual-luciferase reporter assays and ChIP

| **Name** | **Forward primer (5'-3')** | **Reverse primer (5'-3')** |
|---|---|---|
| PGL3.0-*LCN2* (promoter) | GAGCTCTTACGCGTGTGGGCTG CTGGGTTCAA | ACAGTACCGGAATGCGGTCTG AGGTGGAGTCC |
| PGL3.0-*SOCS3* (promoter) | GAGCTCTTACGCGTGAACGGA AGCCCAGAGAAACT | ACAGTACCGGAATGCCGCGGA AGTTAGGTCTCCAG |
| ChIP-*LCN2*-P | AAGCAACAGGTGTCCAGAGC | GGACCCTTCCTCTCCTGTGA |
| ChIP-*SOCS3*-P1 | CCTGACCCGCAGTTGGG | AGAGCGGGCAGTTCTAGGAG |
| ChIP-*SOCS3*-P2 | CCTCTGCCAGAAATCAGCCT | CAGGGACCGGGAGGGAC |
| ChIP-*SOCS3*-P3 | CTCCAGGTCGGCCTCCTA | AGGCTGATTTCTGGCAGAGG |

Supplementary **Table 7**. Information on healthy controls and patients with subclinical hypothyroidism.

| **Code** | **Sex** | **Age** (Year) | **TSH** (μIU/mL) | **FT4** (μg/dL) | **FT3** (μg/dL) |
|---|---|---|---|---|---|
| Control#1 | Male | 54 | 3.45 | 15.0 | 4.86 |
| Control#2 | Female | 27 | 1.83 | 18.0 | 5.4 |
| Control#3 | Male | 67 | 0.925 | 18.7 | 4.5 |
| Control#4 | Male | 65 | 1.61 | 14.1 | 4.49 |
| Control#5 | Male | 40 | 3.14 | 16 | 3.88 |
| Control#6 | Male | 37 | 2.12 | 16.1 | 4.81 |
| Control#7 | Female | 63 | 2.07 | 15.9 | 4.21 |
| Control#8 | Male | 41 | 2.11 | 15.9 | 4.54 |
| Control#9 | Male | 56 | 3.04 | 12.5 | 4.09 |
| Control#10 | Male | 54 | 2.45 | 16 | 4.8 |
| Control#11 | Female | 74 | 2.89 | 19.6 | 3.8 |
| Control#12 | Male | 38 | 0.769 | 18.6 | 4.66 |
| Control#13 | Male | 69 | 1.18 | 16.6 | 5.16 |
| Control#14 | Female | 52 | 2.67 | 13.7 | 4.36 |
| Control#15 | Female | 41 | 1.53 | 13.1 | 4.04 |
| Control#16 | Male | 50 | 1.2 | 20.1 | 5.48 |
| Control#17 | Female | 55 | 1.27 | 18.3 | 4.47 |
| Control#18 | Male | 42 | 1.14 | 8.0 | 4.62 |
| Control#19 | Male | 27 | 1.1 | 18.4 | 4.2 |
| Control#20 | Female | 34 | 0.899 | 14.6 | 4.81 |
| Control#21 | Female | 40 | 0.584 | 15 | 5.06 |
| Control#22 | Female | 58 | 2.18 | 16.0 | 4.32 |
| Control#23 | Female | 32 | 1.34 | 14.3 | 3.97 |
| Control#24 | Female | 31 | 3.66 | 18.2 | 4.42 |
| Control#25 | Female | 58 | 2.07 | 17.0 | 4.38 |
| Control#26 | Female | 61 | 1.87 | 14.0 | 3.83 |
| Patient#1 | Female | 76 | 6.9 | 16.5 | 4.54 |
| Patient#2 | Female | 46 | 5.55 | 15.2 | 4.77 |
| Patient#3 | Female | 74 | 6.23 | 15.9 | 3.81 |
| Patient#4 | Female | 75 | 7.43 | 14.7 | 3.14 |
| Patient#5 | Male | 71 | 5.41 | 13.4 | 3.57 |
| Patient#6 | Male | 79 | 7.23 | 14.8 | 4.75 |
| Patient#7 | Male | 56 | 11.5 | 12.7 | 4.14 |

| | | | | | |
|---|---|---|---|---|---|
| Patient#8 | Male | 49 | 7.19 | 14 | 3.39 |
| Patient#9 | Female | 51 | 5.73 | 14.2 | 3.23 |
| Patient#10 | Male | 70 | 6.38 | 15.1 | 4.34 |
| Patient#11 | Female | 65 | 7.7 | 13.5 | 3.83 |
| Patient#12 | Male | 50 | 5.18 | 13.3 | 5.12 |
| Patient#13 | Female | 40 | 5.18 | 13.3 | 5.12 |
| Patient#14 | Female | 72 | 7.8 | 12.7 | 3.66 |
| Patient#15 | Male | 55 | 5.9 | 15 | 3.72 |
| Patient#16 | Male | 63 | 6.31 | 16.4 | 3.99 |
| Patient#17 | Male | 33 | 6.12 | 16.5 | 5.51 |
| Patient#18 | Female | 70 | 6.19 | 13.9 | 4.09 |
| Patient#19 | Female | 55 | 5.41 | 12.2 | 4.37 |
| Patient#20 | Female | 41 | 9.4 | 15 | 4.02 |
| Patient#21 | Male | 63 | 14.3 | 14 | 4.71 |
| Patient#22 | Female | 75 | 6.74 | 13.9 | 4.67 |
| Patient#23 | Male | 59 | 6.61 | 16.5 | 4.19 |
| Patient#24 | Male | 72 | 4.85 | 22.0 | 4.38 |
| Patient#25 | Female | 36 | 8.57 | 13.0 | 4.85 |
| Patient#26 | Female | 48 | 9.57 | 14.11 | 4.15 |

# Supplementary Figures

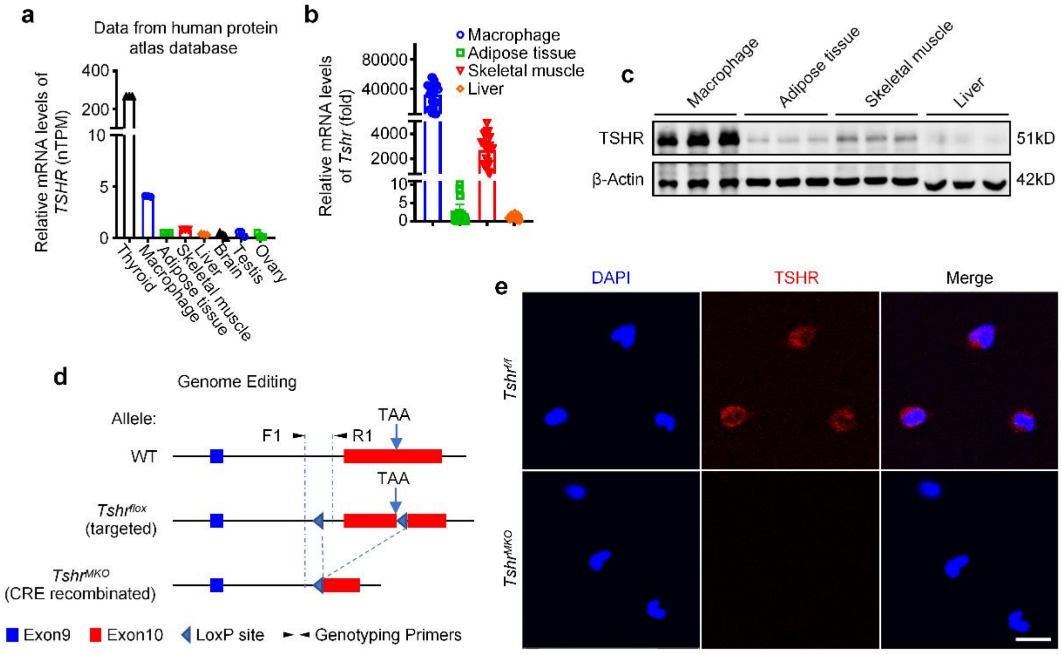

**Supplementary Fig. 1 A mouse model of myeloid-specific *Tshr* knockout. a** The mRNA levels of human *TSHR* in different tissues (data from the human protein atlas database). The mRNA and protein levels of *Tshr* in different tissues of C57BL/6N mice (*n* =3) were assessed by qRT-PCR (**b**) and western blotting (**c**) assays. **d** Schematic illustration of the manipulated locus in *Tshr*$^{f/f}$ mice. The primers (F1 and R1) were used for genotyping. **e** Representative immunofluorescence staining of TSHR (red) in BMDMs. Nuclei were stained with DAPI (blue). Scale bars:10 µm.

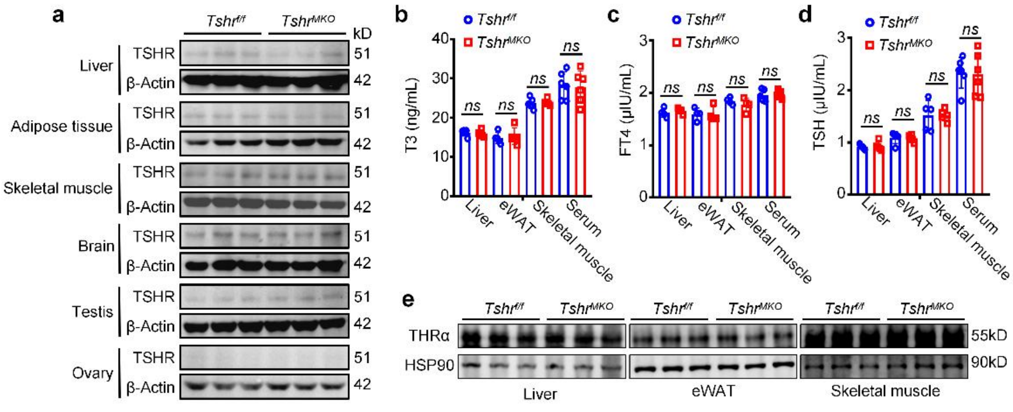

**Supplementary Fig. 2 *Tshr* knockout in myeloid cells has no effect on thyroid function in**

**mice.**

**a** The protein levels of TSHR in different tissues of C57BL/6N mice ($n$ =3) were assessed by western blotting analysis. $\beta$-Actin was used as a loading control. The levels of T3 (**b**), FT4 (**c**) and TSH (**d**) in liver, eWAT, skeletal muscle and serum of $Tshr^{MKO}$ and $Tshr^{f/f}$ mice ($n$ =4-6). **e** The protein levels of THRα in different tissues of C57BL/6N mice ($n$ =3) were assessed by western blotting analysis. HSP90 was used as a loading control. Data are presented as mean ± standard deviation (SD). *ns*, no significance (1-way ANOVA for **b-d**).

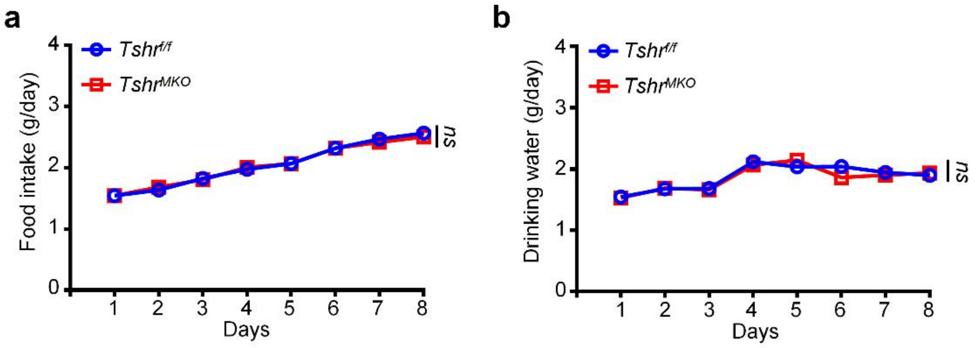


**Supplementary Fig. 3** Food intake (**a**) and drinking water (**b**) of $Tshr^{MKO}$ and $Tshr^{f/f}$ mice (male, 6 weeks old), which were fed with HFD for 9 weeks ($n$ =6). Data are presented as mean ± standard error (SE). *ns*, no significance (two-way ANOVA for **a**, **b**).

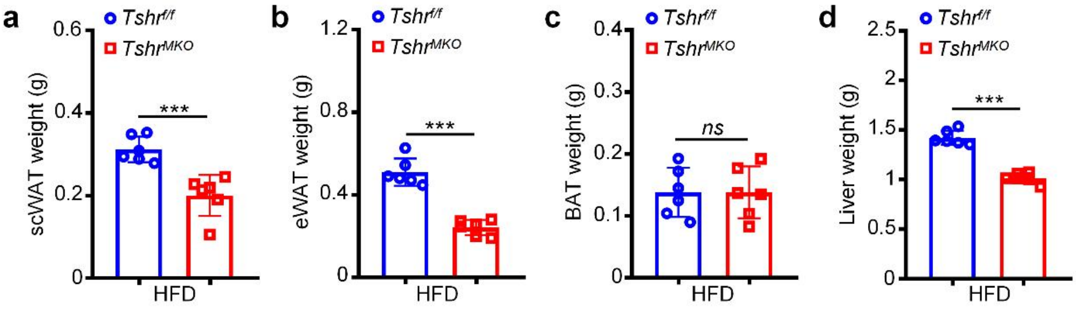


**Supplementary Fig. 4** $Tshr^{MKO}$ and $Tshr^{f/f}$ mice (male, 6 weeks old) were fed with HFD for 9 weeks. The weights of scWAT (**a**), eWAT (**b**), BAT (**c**) and liver (**d**) were then measured after euthanasia ($n$ =6). Data are presented as mean ± standard deviation (SD). ***$P < 0.001$, *ns*, no significance (unpaired two tailed Student's $t$ test for **a-d**).

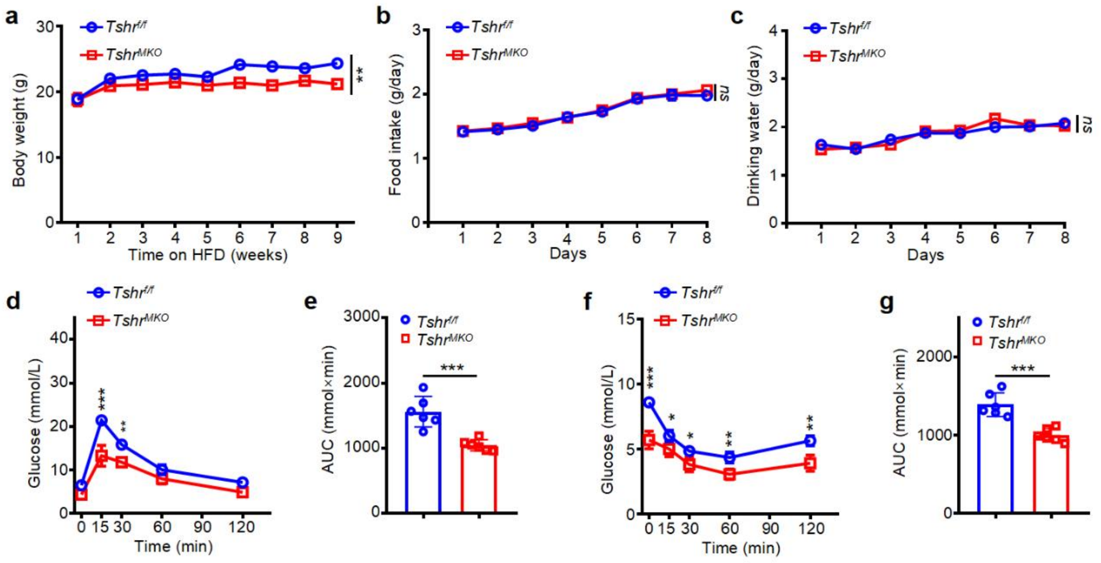


**Supplementary Fig. 5 Metabolic characterizations of HFD-fed female *Tshr*$^{MKO}$ and *Tshr*$^{f/f}$ mice.** *Tshr*$^{MKO}$ mice and age-matched *Tshr*$^{f/f}$ littermates (female, 6 weeks old) were fed with HFD for 9 weeks. Body weight (**a**), food intake (**b**), drinking water (**c**), glucose tolerance test (**d**) and AUC (**e**) as well as insulin tolerance test (**f**) and AUC (**g**) were then measured. Data are presented as mean ± standard error (SE) in (**a-d**, **f**), and as mean ± standard deviation (SD) in (**e**, **g**) *$P < 0.05$, **$P < 0.01$, ***$P < 0.001$, *ns*, no significance (two-way ANOVA for **a-d**, **f**, unpaired two tailed Student's *t* test for **e**, **g**).

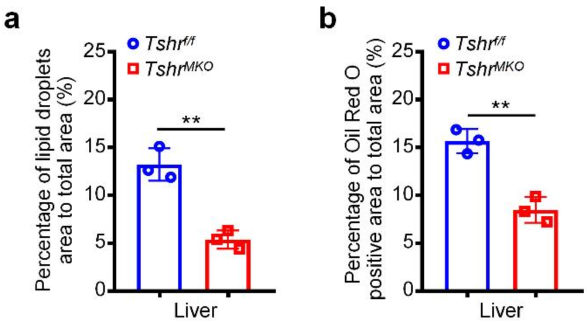


**Supplementary Fig. 6 Myeloid *Tshr* deficiency protects from HFD-induced hepatic steatosis.** **a** The percentage of lipid droplets area to total area of liver sections in Fig 2d ($n$ =3). **b** The percentage of Oil Red O positive area to total area of liver sections in Fig 2e ($n$ =3). Data are presented as mean ± standard deviation (SD). **$P < 0.01$ (unpaired two tailed Student's *t* test for **a**, **b**)

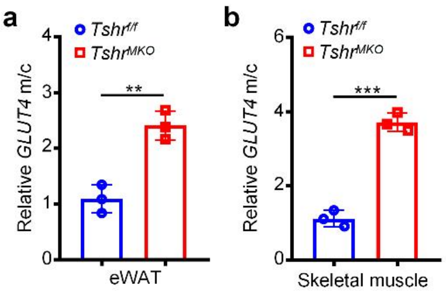


**Supplementary Fig. 7 Myeloid *Tshr* deficiency enhances the membrane localization of GLUT4 in eWAT and skeletal muscle tissues of HFD-fed male mice.** Relative GLUT4 cytomembrane/cytoplasm (m/c) of eWAT sections (**a**) and skeletal muscle sections (**b**) of HFD-fed male mice in Fig 2f ($n$ =3). Data are presented as mean ± standard deviation (SD). $^{**}P < 0.01$, $^{***}P < 0.001$ (unpaired two tailed Student's $t$ test for **a**, **b**).

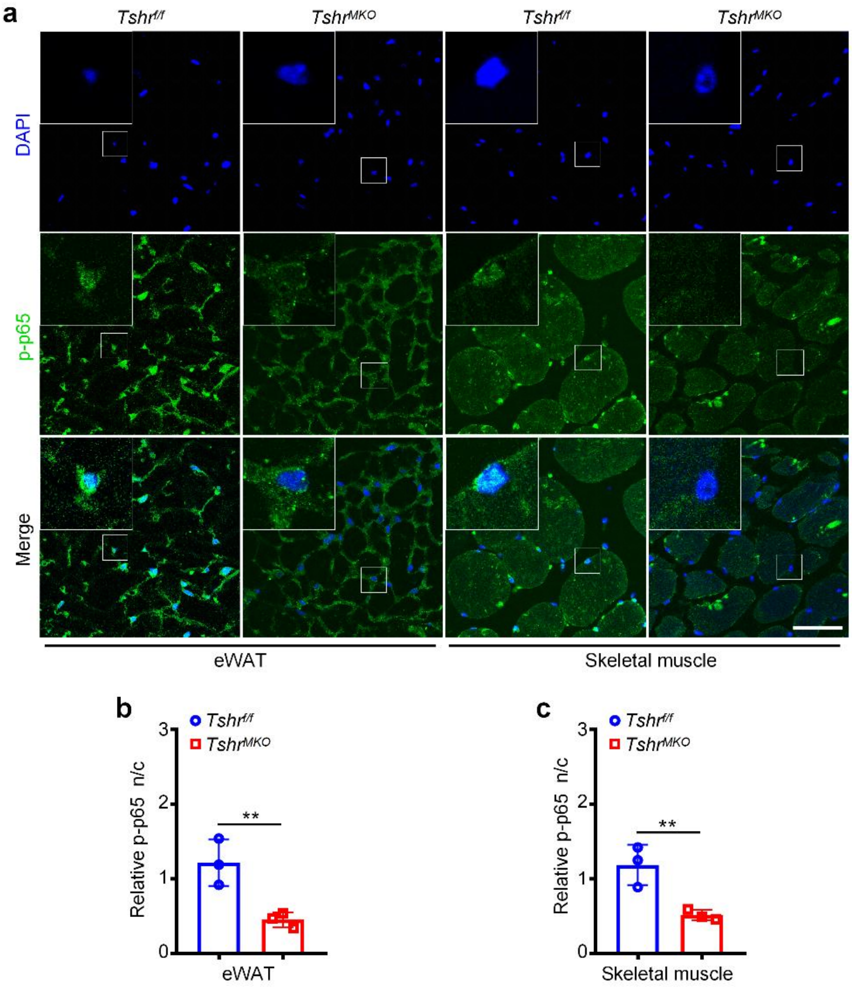


**Supplementary Fig. 8 Myeloid *Tshr* deficiency reduces the nucleus localization of p-p65 in eWAT and skeletal muscle sections tissues of HFD-fed male mice. a** Representative immunofluorescence staining of p-p65 (green) in eWAT and skeletal muscle sections, Nuclei were

stained with DAPI (blue). Showed on the upper left corner is an enlarged image. Scale bars: 50 μm. Relative p-p65 nucleus/cytoplasm (n/c) of eWAT sections (**b**) and skeletal muscle sections (**c**) of HFD-fed male mice in Supplementary Fig. 8a ($n$ = 3). Data are presented as mean ± standard deviation (SD). **$P$ < 0.01 (unpaired two tailed Student's $t$ test for **b**, **c**).

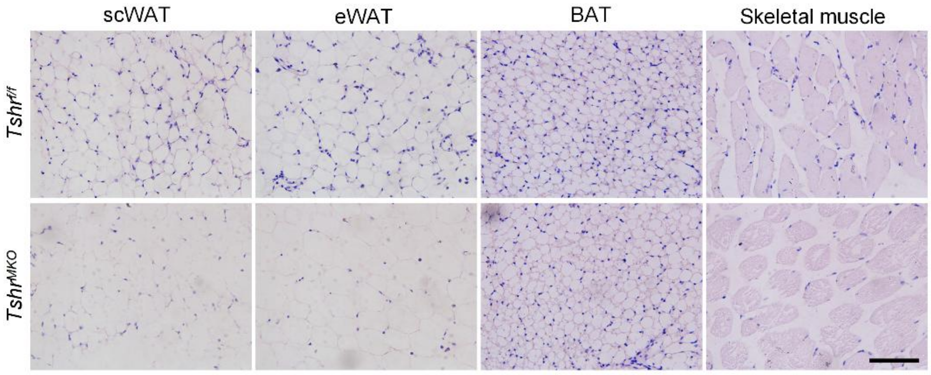


**Supplementary Fig. 9** Representative H&E staining of scWAR, eWAT, BAT and skeletal muscle sections in *Tshr*$^{MKO}$ and *Tshr*$^{f/f}$ mice. Scale bars: 100 μm.

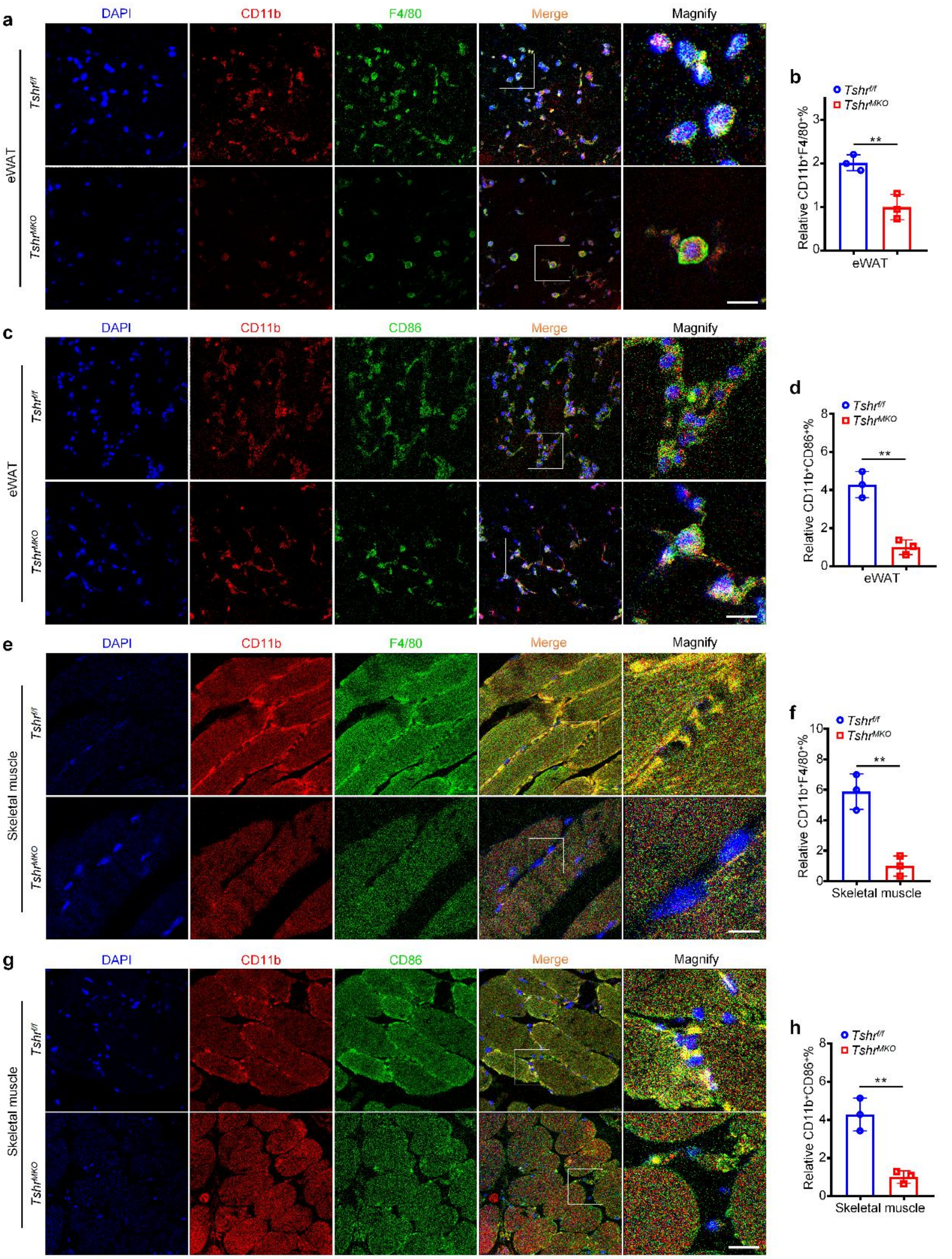


**Supplementary Fig. 10 Myeloid *Tshr* deficiency alleviates macrophage infiltration and M1 polarization in adipose tissue and skeletal muscle of HFD-fed *Tshr*$^{MKO}$ and *Tshr*$^{f/f}$ mice.** *Tshr*$^{MKO}$ mice and age-matched *Tshr*$^{f/f}$ littermates (male, 6 weeks old) were fed with HFD for 9 weeks. **a** Representative immunofluorescence staining of CD11b (red) and F4/80 (green) in eWAT sections. Nuclei were stained with DAPI (blue). Scale bars: 10 μm. **b** The relative percentage of

CD11b$^+$F4/80$^+$ in all cells of eWAT sections ($n$ = 3). **c** Representative immunofluorescence staining of CD11b (red) and CD86 (green) in eWAT sections. Nuclei were stained with DAPI (blue) Scale bars: 10 μm. **d** The relative percentage of CD11b$^+$CD86$^+$ in all cells of eWAT sections ($n$ = 3). **e** Representative immunofluorescence staining of CD11b (red) and F4/80 (green) in skeletal muscle sections. Nuclei were stained with DAPI (blue). Scale bars: 10 μm. **f** The relative percentage of CD11b$^+$F4/80$^+$ in all cells of skeletal muscle sections ($n$ = 3). **g** Representative immunofluorescence staining of CD11b (red) and CD86 (green) in skeletal muscle sections. Nuclei were stained with DAPI (blue). Scale bars: 10 μm. **h** The relative percentage of CD11b$^+$CD86$^+$ in all cells of skeletal muscle sections ($n$ = 3). Data are presented as mean ± standard deviation (SD). **$P < 0.01$ (unpaired two tailed Student's $t$ test for **b**, **d**, **f**, **h**).

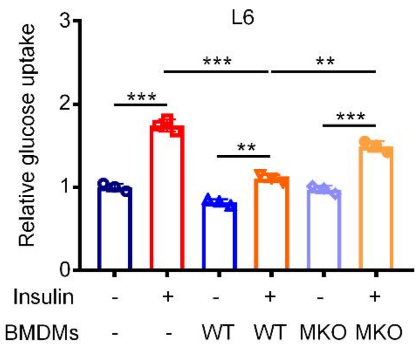


**Supplementary Fig. 11** L6 differentiated skeletal muscle cells were co-cultivated with *Tshr$^{f/f}$*- or *Tshr$^{MKO}$*-derived BMDMs treated with the indicated conditions. Relative glucose uptake was then measured ($n$ =3). Data are presented as mean ± standard deviation (SD). **$P < 0.01$, ***$P < 0.001$ (1-way ANOVA).

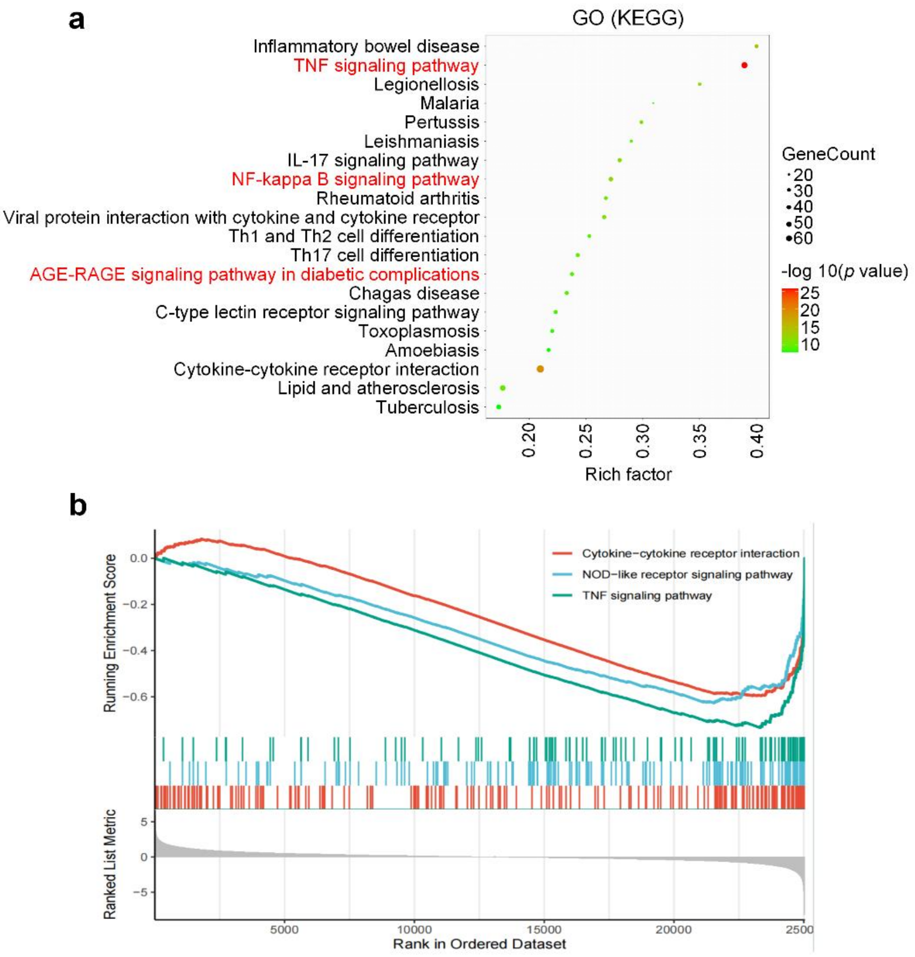


**Supplementary Fig. 12** GO analysis (**a**) and GSEA (**b**) of mRNA sequencing data in *Tshr*$^{f/f}$- or *Tshr*$^{MKO}$-derived BMDMs.

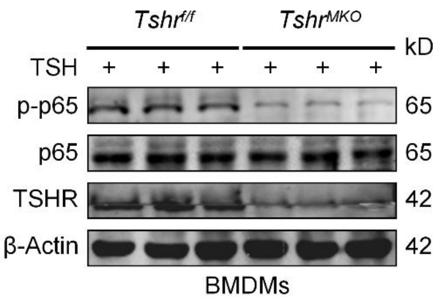


**Supplementary Fig. 13** Western blotting analysis was performed to determine the levels of TSHR and p-p65 in BMDMs from *Tshr*$^{MKO}$ mice and age-matched *Tshr*$^{f/f}$ littermates stimulated by 1 ng/mL TSH for 24 h. $\beta$-Actin was used as a loading control.

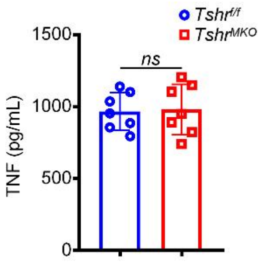


**Supplementary Fig. 14** *Tshr*$^{MKO}$ mice and age-matched *Tshr*$^{f/f}$ littermates (male, 6 weeks old) were fed with HFD for 9 weeks. Serum concentrations of TNF of these mice were determined by ELISA (*n* =7). Data are presented as mean ± standard deviation (SD). *ns*, no significance (unpaired two tailed Student's *t* test).

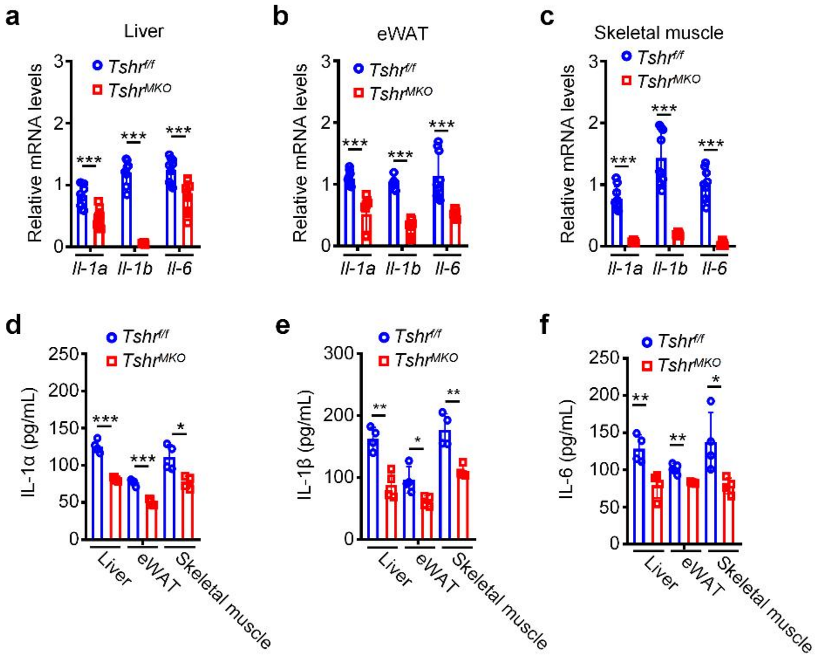


**Supplementary Fig. 15 Myeloid *Tshr* deficiency reduces the levels of cytokines in liver, adipose and skeletal muscle tissues.** qRT-PCR assays were performed to determine the mRNA levels of *Il-1a*, *Il-1b* and *Il-6* in liver (**a**), eWAT (**b**) and skeletal muscle (**c**) of *Tshr*$^{MKO}$ and *Tshr*$^{f/f}$ mice (n =9). The protein levels of IL-1α (**d**), IL-1β (**e**) and IL-6 (**f**) in these mice were then measured by ELISA (n = 6). Data are presented as mean ± standard deviation (SD). *$P < 0.05$, **$P < 0.01$, ***$P < 0.001$ (1-way ANOVA for **a-f**).

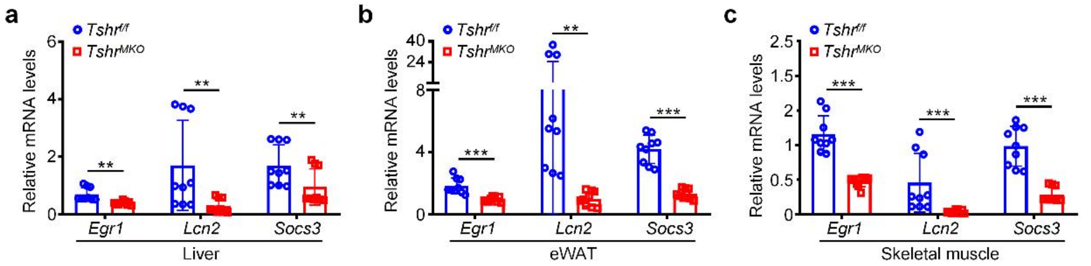


**Supplementary Fig. 16** *Tshr*$^{MKO}$ mice and age-matched *Tshr*$^{f/f}$ littermates (male, 6 weeks old) were fed with HFD for 9 weeks. The mRNA levels of *Egr1*, *Lcn2* and *Socs3* in liver (**a**), eWAT (**b**) and skeletal muscle (**c**) of *Tshr*$^{MKO}$ and *Tshr*$^{f/f}$ mice were determined by qRT-PCR ($n$ = 9). Data are presented as mean ± standard deviation (SD). $^{**}P < 0.01$, $^{***}P < 0.001$ (1-way ANOVA for **a**-**c**).

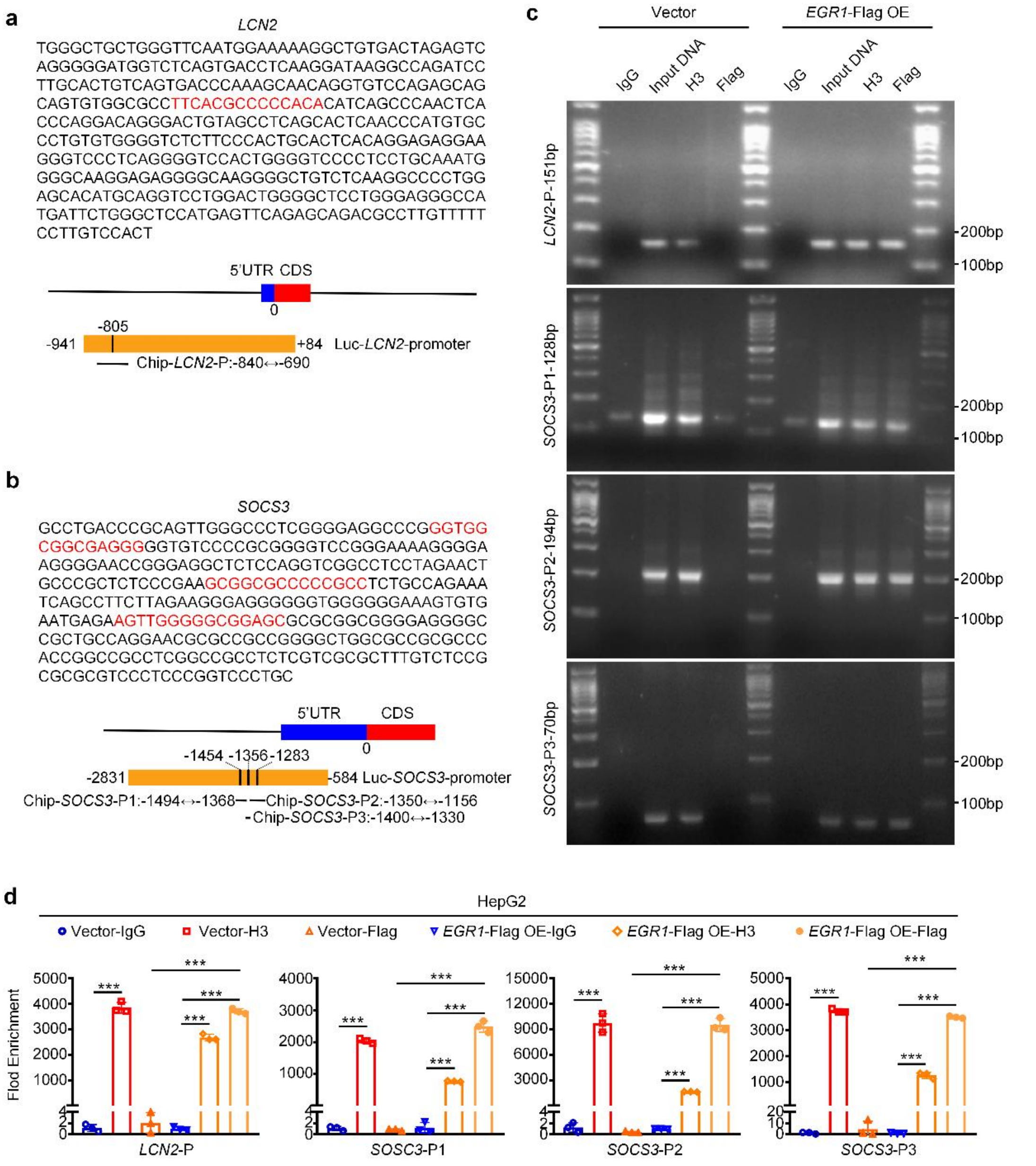


**Supplementary Fig. 17 EGR1 activates the transcription of *LCN2* and *SOCS3*.** DNA sequences (upper panel) and schematic representation (lower panel) of the predicted *EGR1* binding sites in the promoters of *LCN2* (**a**) and *SOCS3* (**b**). **c** HepG2 cells were transfected with *EGR1*-Flag-overexpressing plasmid or vector plasmid. ChIP assays were performed to confirm the binding of *EGR1* to the promoter regions of *LCN2* and *SOCS3*, including one promoter fragment of *LCN2* (LCN-P) and three promoter fragments of *SOCS3* (*SOCS3*-P1, *SOCS3*-P2 and *SOCS3*-P3). Cross-linked chromatin isolated from the indicated cells was immunoprecipitated with nonspecific IgG (lanes 1 and 5), positive control anti-H3 (lanes 3 and 7) and anti-Flag (lanes 4 and 8). Chromosomal DNA which was not immunoprecipitated was regarded as input (lanes 2

and 6). PCR products were separated on a 2% agarose gel containing ethidium bromide and detected via ultraviolet illumination. **d** qPCR were performed for immunoprecipitated DNA to confirm the binding of *EGR1* to the promoter regions of *LCN2* and *SOCS3*, including one promoter fragment of *LCN2* (*LCN*-P) and three promoter fragments of *SOCS3* (*SOCS3*-P1, *SOCS3*-P2 and *SOCS3*-P3) ($n$ =3). Data are presented as mean ± standard deviation (SD). ***$P <$ 0.001 (1-way ANOVA for **d**).
