# Supplementary material for: Thyrotropin exacerbates insulin resistance by triggering macrophage inflammation in subclinical hypothyroidism
Source: Exp Mol Med. 2025 Jun 16;57(6):1246–59. doi: 10.1038/s12276-025-01478-1 (PMC12229657; doi:10.1038/s12276-025-01478-1)

Figure1-a

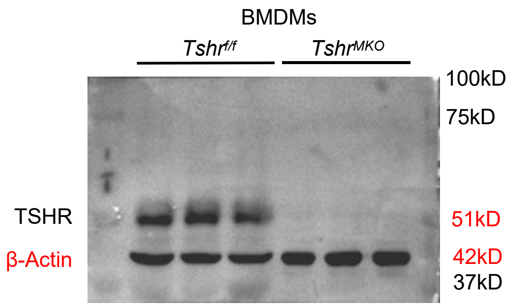

Figure2-a

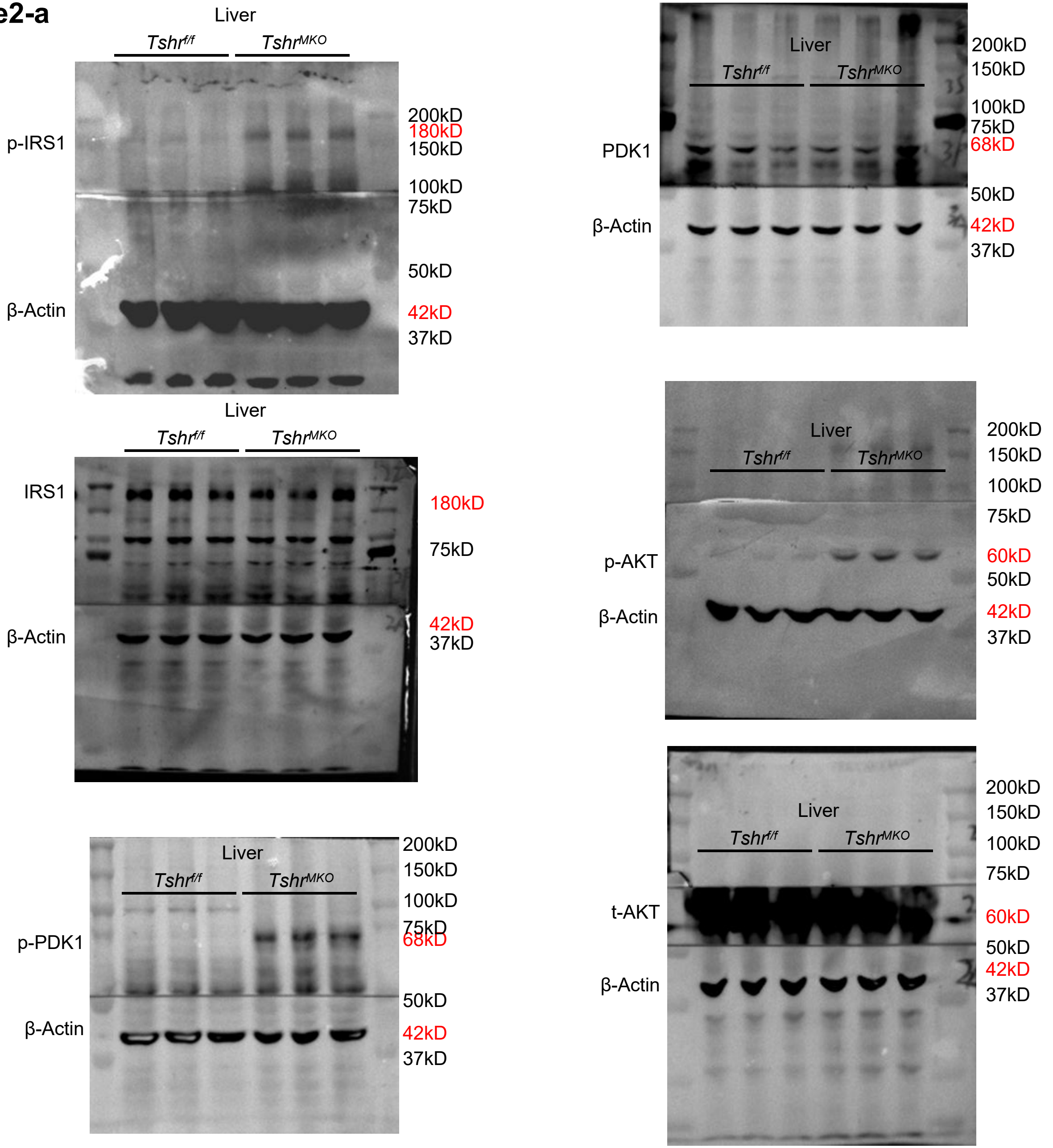
Liver
Tshr f/f
Tshr MKO
p-IRS1
β-Actin
200kD
180kD
150kD
100kD
75kD
50kD
42kD
37kD
IRS1
p-PDK1
68kD
PDK1
60kD
p-AKT
t-AKT

Figure2-a

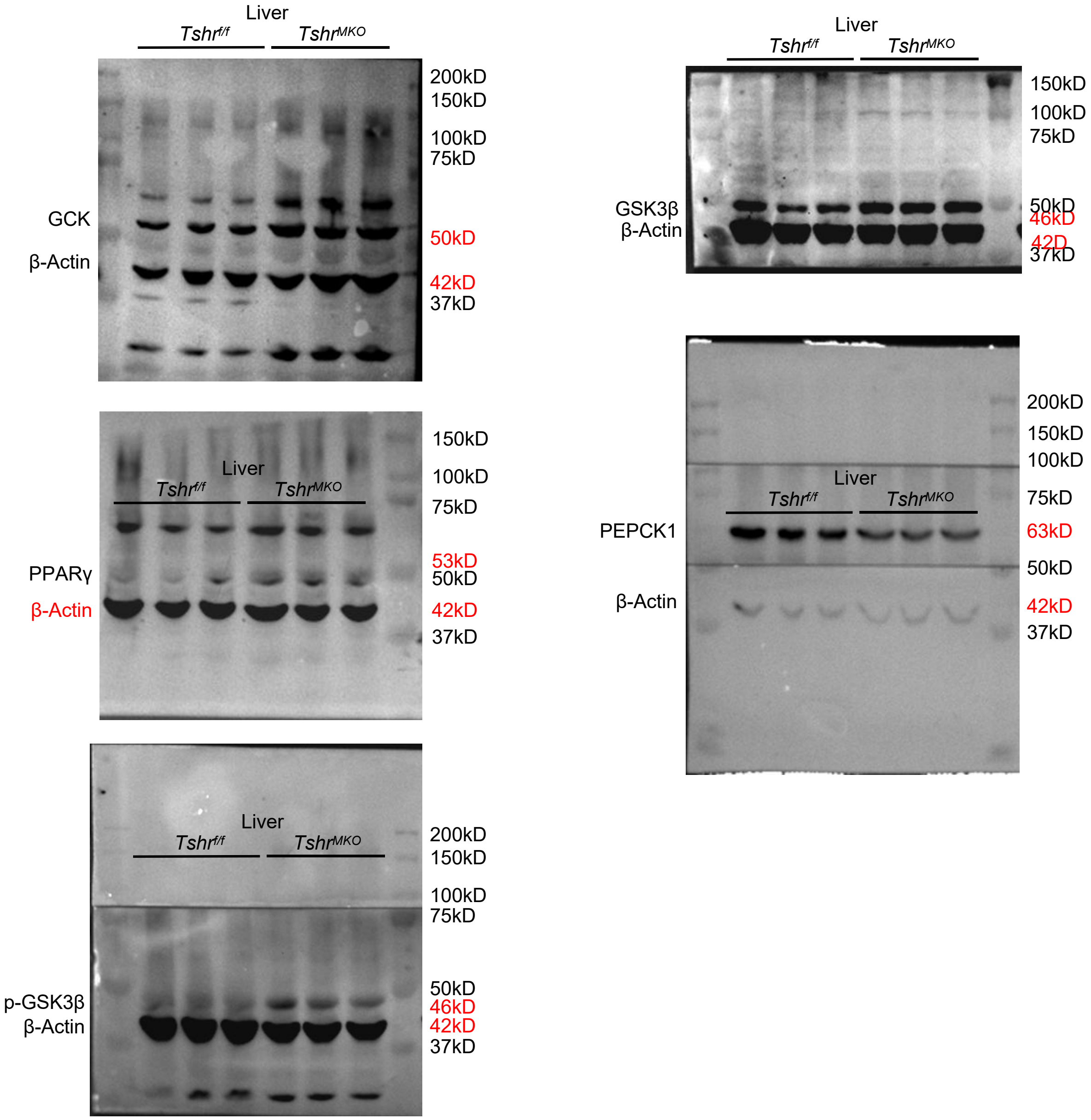

Liver
Tshrf/f
TshrMKO
GCK
β-Actin
200kD
150kD
100kD
75kD
50kD
42kD
37kD
PPARγ
β-Actin
150kD
100kD
75kD
53kD
50kD
42kD
37kD
p-GSK3β
β-Actin
200kD
150kD
100kD
75kD
50kD
46kD
42kD
37kD
GSK3β
β-Actin
150kD
100kD
75kD
50kD
46kD
42D
37kD
PEPCK1
β-Actin
200kD
150kD
100kD
75kD
63kD
50kD
42kD
37kD

Figure2-b

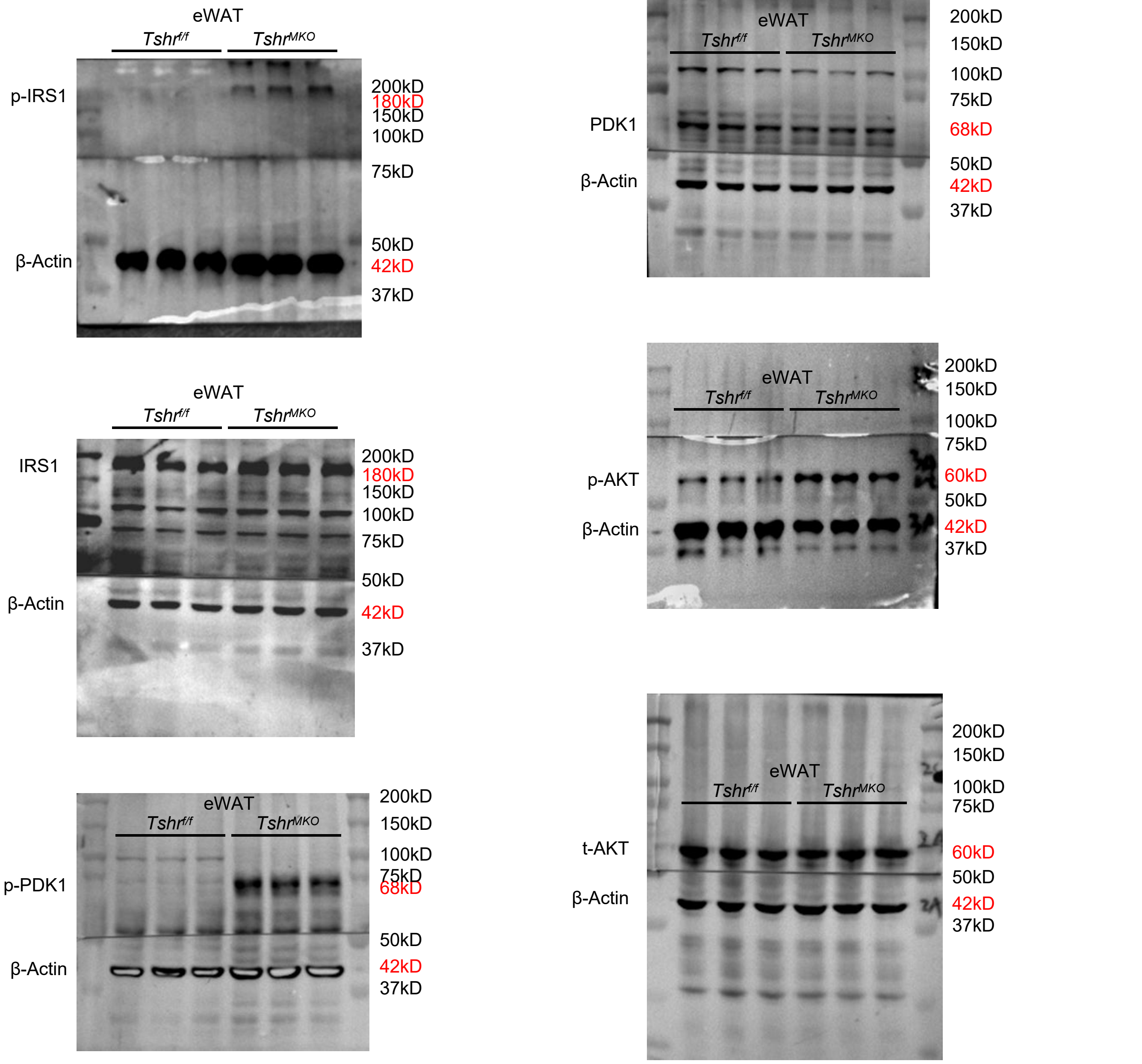
eWAT
Tshr f/f
Tshr MKO
p-IRS1
β-Actin
200kD
180kD
150kD
100kD
75kD
50kD
42kD
37kD
eWAT
Tshr f/f
Tshr MKO
IRS1
β-Actin
200kD
180kD
150kD
100kD
75kD
50kD
42kD
37kD
eWAT
Tshr f/f
Tshr MKO
p-PDK1
β-Actin
200kD
150kD
100kD
75kD
68kD
50kD
42kD
37kD
eWAT
Tshr f/f
Tshr MKO
PDK1
β-Actin
200kD
150kD
100kD
75kD
68kD
50kD
42kD
37kD
eWAT
Tshr f/f
Tshr MKO
p-AKT
β-Actin
200kD
150kD
100kD
75kD
60kD
50kD
42kD
37kD
eWAT
Tshr f/f
Tshr MKO
t-AKT
β-Actin
200kD
150kD
100kD
75kD
60kD
50kD
42kD
37kD

Figure2-b

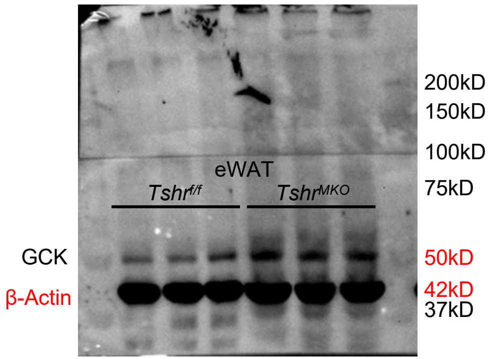
eWAT
Tshrf/f
TshrMKO
GCK
β-Actin
200kD
150kD
100kD
75kD
50kD
42kD
37kD

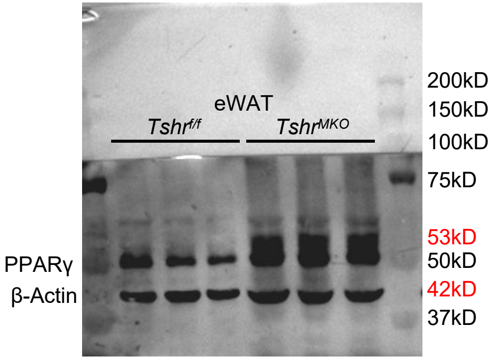
eWAT
Tshrf/f
TshrMKO
PPARγ
β-Actin
200kD
150kD
100kD
75kD
53kD
50kD
42kD
37kD

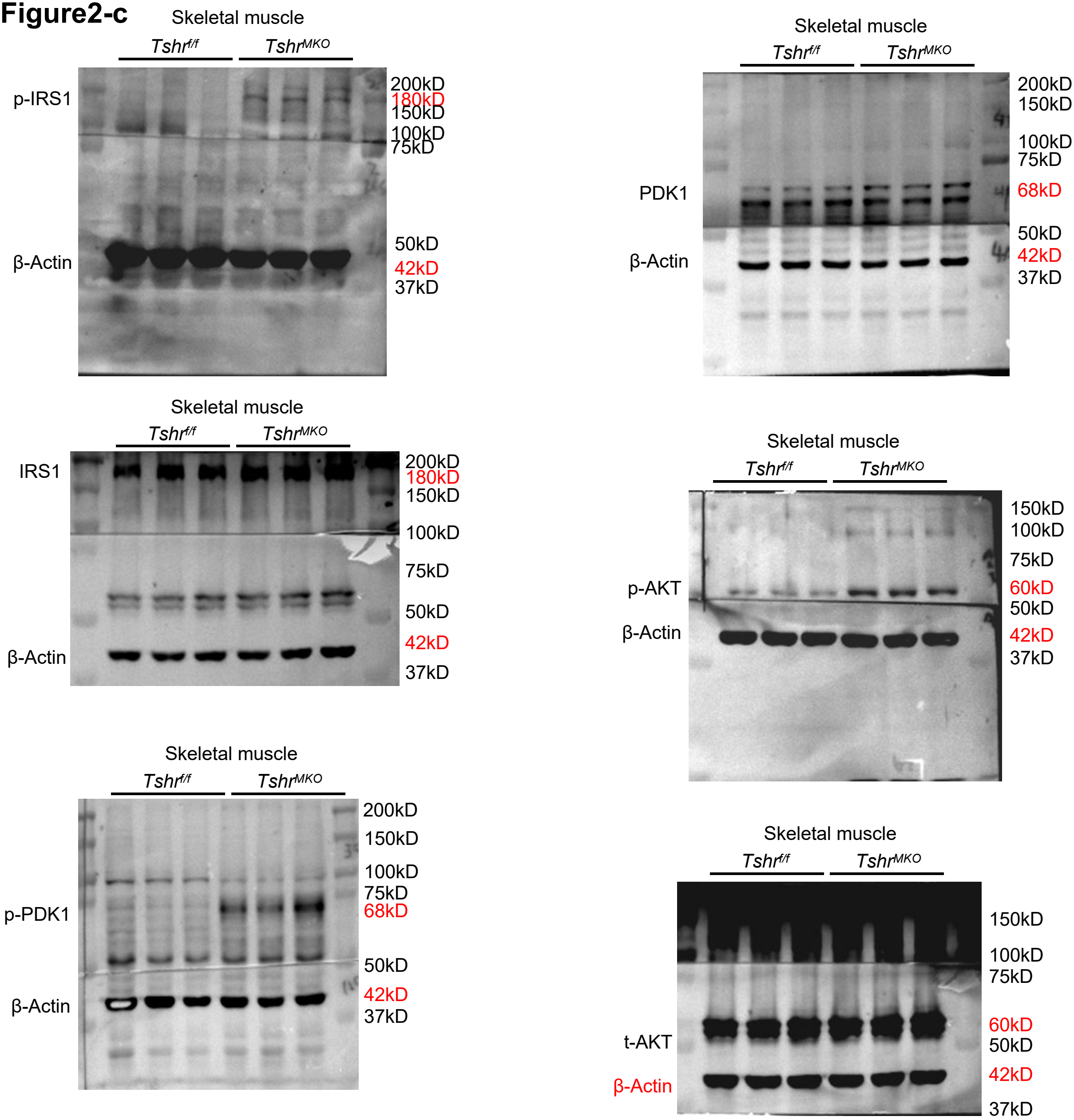
Skeletal muscle
Tshrf/f
TshrMKO
p-IRS1
β-Actin
200kD
180kD
150kD
100kD
75kD
50kD
42kD
37kD
Skeletal muscle
Tshrf/f
TshrMKO
PDK1
β-Actin
200kD
150kD
100kD
75kD
68kD
50kD
42kD
37kD
Skeletal muscle
Tshrf/f
TshrMKO
IRS1
β-Actin
200kD
180kD
150kD
100kD
75kD
50kD
42kD
37kD
Skeletal muscle
Tshrf/f
TshrMKO
p-AKT
β-Actin
150kD
100kD
75kD
60kD
50kD
42kD
37kD
Skeletal muscle
Tshrf/f
TshrMKO
p-PDK1
β-Actin
200kD
150kD
100kD
75kD
68kD
50kD
42kD
37kD
Skeletal muscle
Tshrf/f
TshrMKO
t-AKT
β-Actin
150kD
100kD
75kD
60kD
50kD
42kD
37kD

Figure2-c

Figure2-c

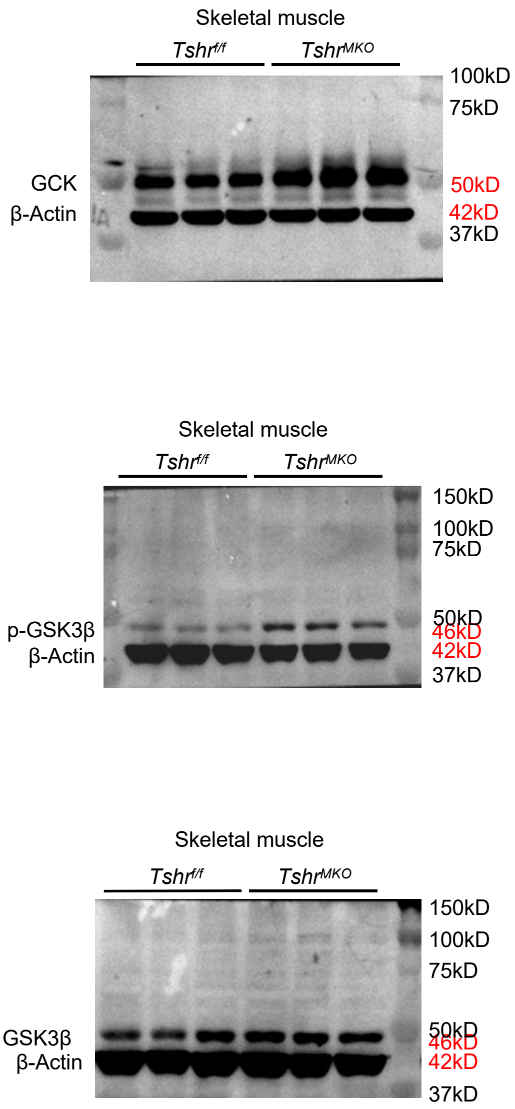
Skeletal muscle
Tshr f/f
Tshr MKO
100kD
75kD
GCK
β-Actin
50kD
42kD
37kD
Skeletal muscle
Tshr f/f
Tshr MKO
150kD
100kD
75kD
p-GSK3β
β-Actin
50kD
46kD
42kD
37kD
Skeletal muscle
Tshr f/f
Tshr MKO
150kD
100kD
75kD
GSK3β
β-Actin
50kD
46kD
42kD
37kD

Figure2-g

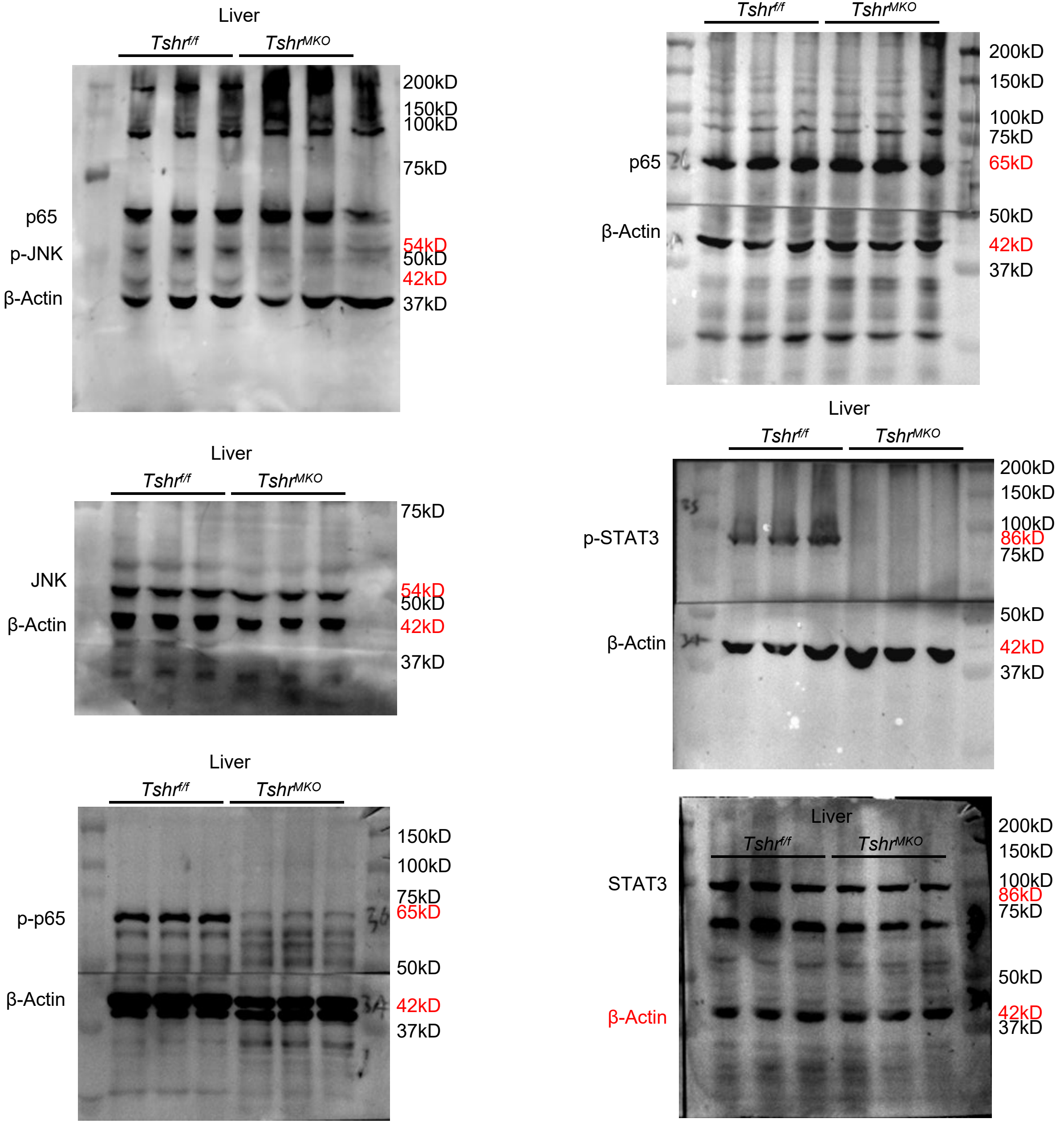

Liver
Tshr^f/f
Tshr^MKO
p65
p-JNK
β-Actin
200kD
150kD
100kD
75kD
54kD
50kD
42kD
37kD
Liver
Tshr^f/f
Tshr^MKO
p65
β-Actin
200kD
150kD
100kD
75kD
65kD
50kD
42kD
37kD
Liver
Tshr^f/f
Tshr^MKO
JNK
β-Actin
75kD
54kD
50kD
42kD
37kD
Liver
Tshr^f/f
Tshr^MKO
p-STAT3
β-Actin
200kD
150kD
100kD
86kD
75kD
50kD
42kD
37kD
Liver
Tshr^f/f
Tshr^MKO
p-p65
β-Actin
150kD
100kD
75kD
65kD
50kD
42kD
37kD
Liver
Tshr^f/f
Tshr^MKO
STAT3
β-Actin
200kD
150kD
100kD
86kD
75kD
50kD
42kD
37kD

Figure2-g

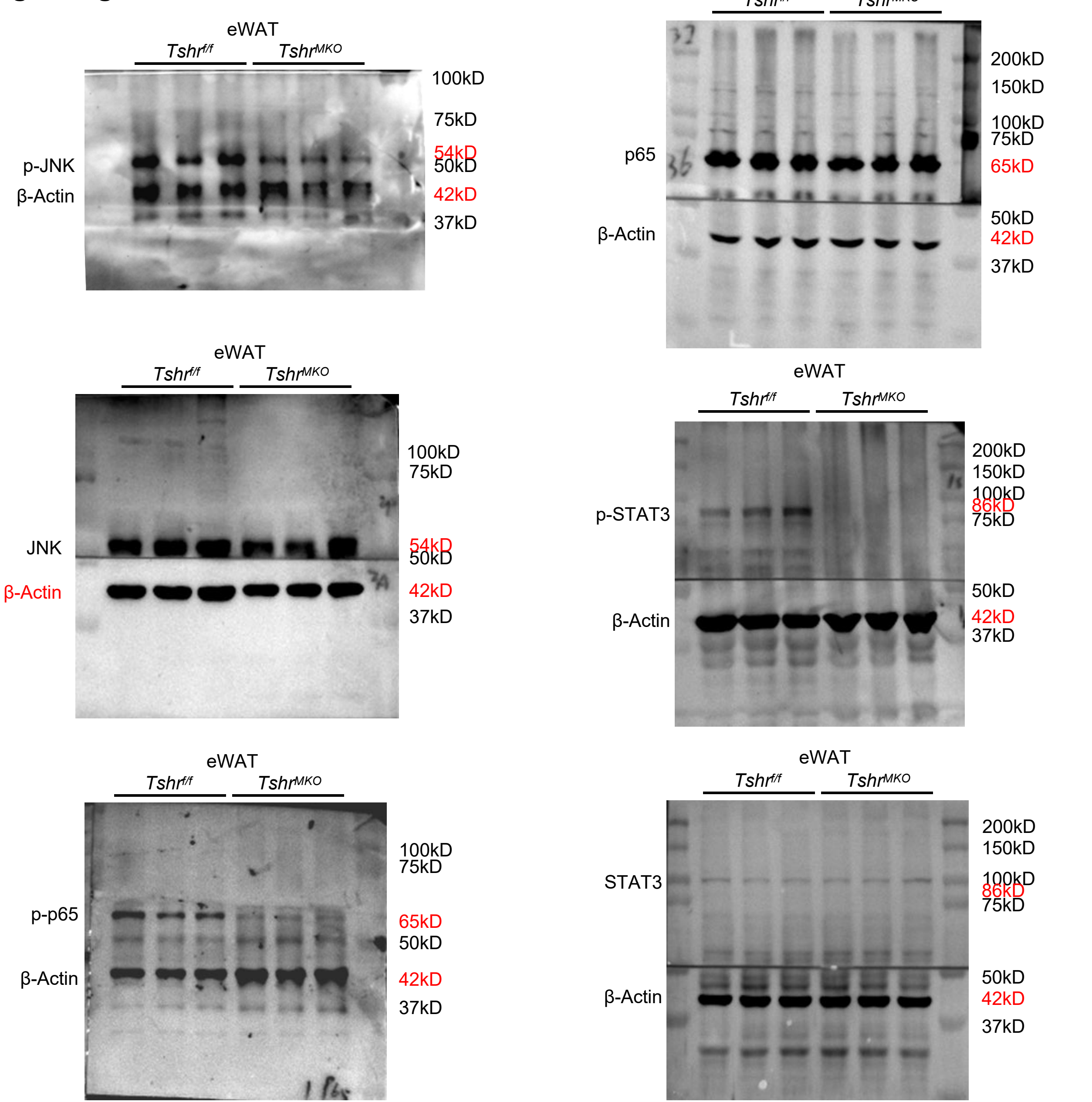
eWAT
Tshr^f/f
Tshr^MKO
p-JNK
β-Actin
100kD
75kD
54kD
50kD
42kD
37kD
eWAT
Tshr^f/f
Tshr^MKO
p65
β-Actin
200kD
150kD
100kD
75kD
65kD
50kD
42kD
37kD
eWAT
Tshr^f/f
Tshr^MKO
JNK
β-Actin
100kD
75kD
54kD
50kD
42kD
37kD
eWAT
Tshr^f/f
Tshr^MKO
p-STAT3
β-Actin
200kD
150kD
100kD
86kD
75kD
50kD
42kD
37kD
eWAT
Tshr^f/f
Tshr^MKO
p-p65
β-Actin
100kD
75kD
65kD
50kD
42kD
37kD
eWAT
Tshr^f/f
Tshr^MKO
STAT3
β-Actin
200kD
150kD
100kD
86kD
75kD
50kD
42kD
37kD

Figure2-g

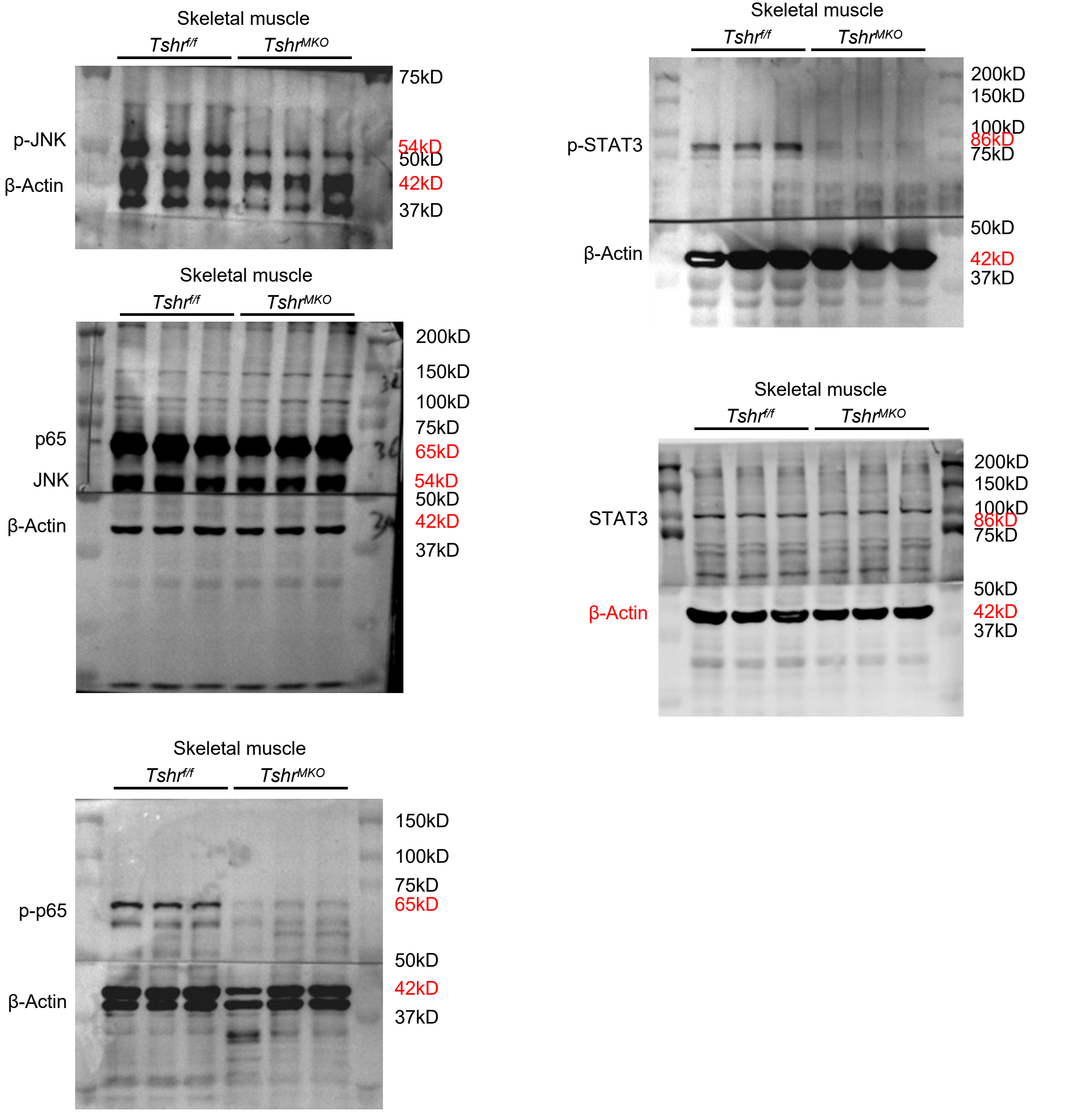
Skeletal muscle
Tshr$^{f/f}$
Tshr^{MKO}
p-JNK
β-Actin
75kD
54kD
50kD
42kD
37kD
Skeletal muscle
Tshr$^{f/f}$
Tshr^{MKO}
p65
JNK
β-Actin
200kD
150kD
100kD
75kD
65kD
54kD
50kD
42kD
37kD
Skeletal muscle
Tshr$^{f/f}$
Tshr^{MKO}
p-p65
β-Actin
150kD
100kD
75kD
65kD
50kD
42kD
37kD
Skeletal muscle
Tshr$^{f/f}$
Tshr^{MKO}
p-STAT3
β-Actin
200kD
150kD
100kD
86kD
75kD
50kD
42kD
37kD
Skeletal muscle
Tshr$^{f/f}$
Tshr^{MKO}
STAT3
β-Actin
200kD
150kD
100kD
86kD
75kD
50kD
42kD
37kD

Figure3-f

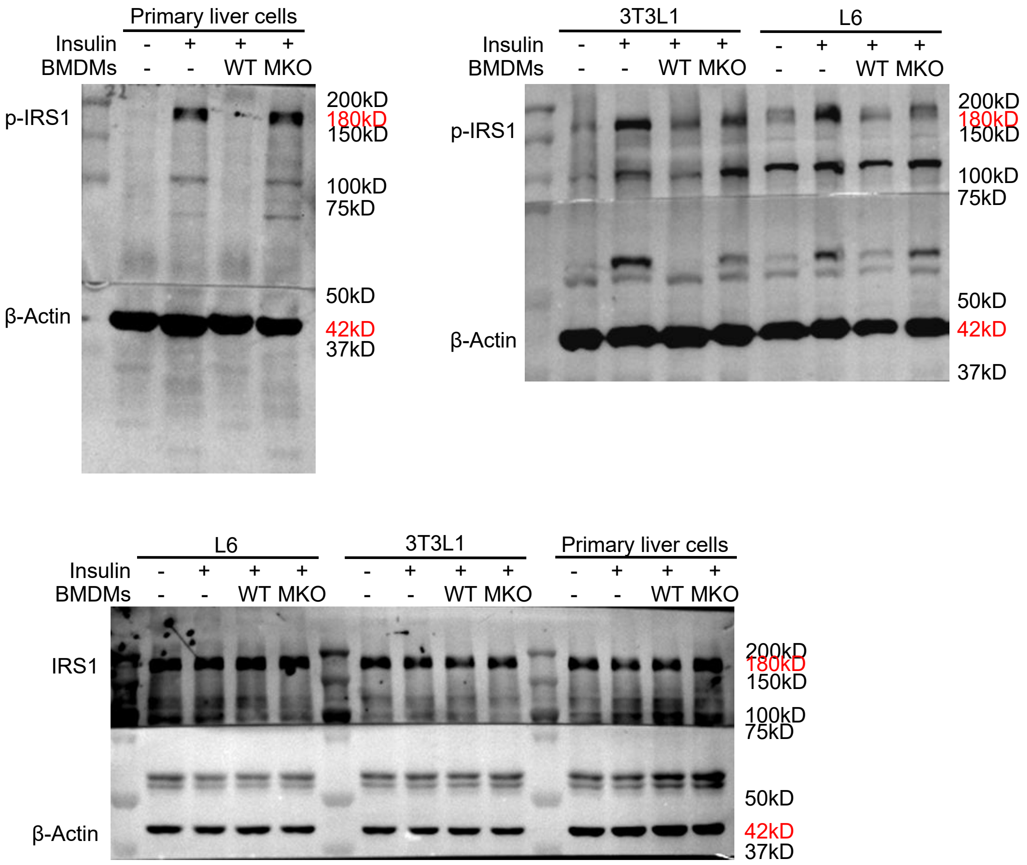

Primary liver cells
Insulin - + + +
BMDMs - - WT MKO
p-IRS1
β-Actin
200kD
180kD
150kD
100kD
75kD
50kD
42kD
37kD
3T3L1
L6
Insulin - + + + - + + +
BMDMs - - WT MKO - - WT MKO
p-IRS1
β-Actin
200kD
180kD
150kD
100kD
75kD
50kD
42kD
37kD
L6
3T3L1
Primary liver cells
Insulin - + + + - + + + - + + +
BMDMs - - WT MKO - - WT MKO - - WT MKO
IRS1
β-Actin
200kD
180kD
150kD
100kD
75kD
50kD
42kD
37kD

Figure3-f

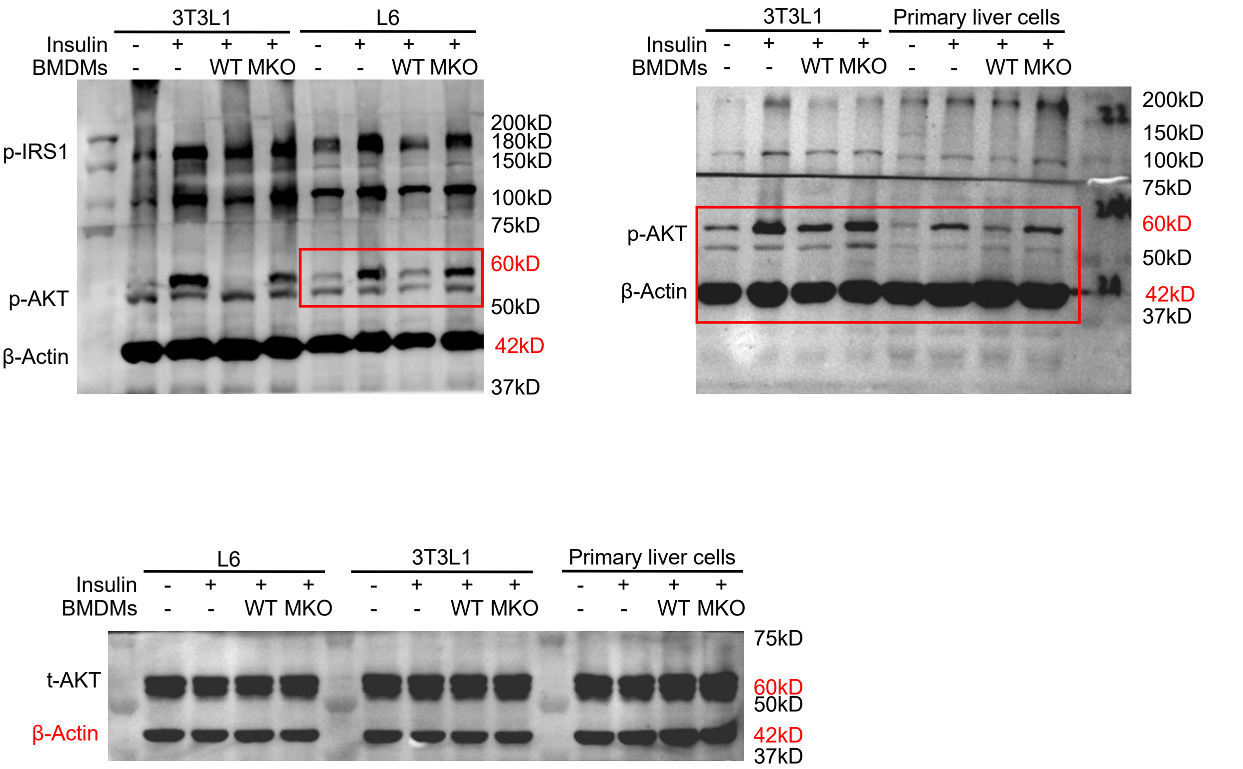

3T3L1
L6
Insulin - + + + - + + +
BMDMs - - WT MKO - - WT MKO
p-IRS1
p-AKT
β-Actin
200kD
180kD
150kD
100kD
75kD
60kD
50kD
42kD
37kD
3T3L1
Primary liver cells
Insulin - + + + - + + +
BMDMs - - WT MKO - - WT MKO
p-AKT
β-Actin
200kD
150kD
100kD
75kD
60kD
50kD
42kD
37kD
L6
3T3L1
Primary liver cells
Insulin - + + + - + + + - + + +
BMDMs - - WT MKO - - WT MKO - - WT MKO
t-AKT
β-Actin
75kD
60kD
50kD
42kD
37kD

Figure3-f

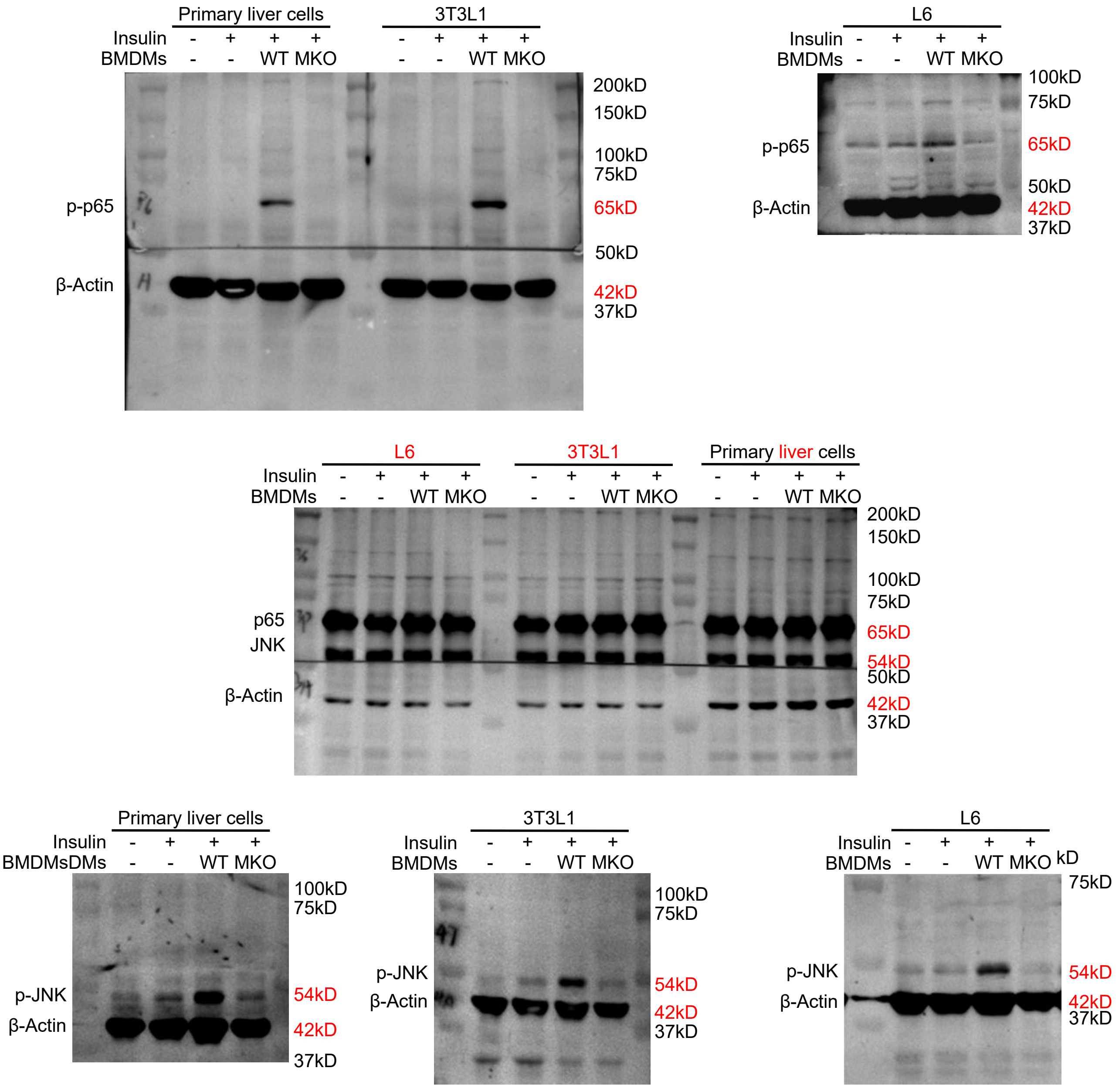
Primary liver cells
3T3L1
Insulin - + + +
BMDMs - - WT MKO
200kD
150kD
100kD
75kD
p-p65
65kD
50kD
β-Actin
42kD
37kD
L6
Insulin - + + +
BMDMs - - WT MKO
100kD
75kD
p-p65
65kD
50kD
β-Actin
42kD
37kD
L6
3T3L1
Primary liver cells
Insulin - + + +
BMDMs - - WT MKO
200kD
150kD
100kD
75kD
p65
JNK
65kD
54kD
50kD
β-Actin
42kD
37kD
Primary liver cells
Insulin - + + +
BMDMsDMs - - WT MKO
100kD
75kD
p-JNK
54kD
β-Actin
42kD
37kD
3T3L1
Insulin - + + +
BMDMs - - WT MKO
100kD
75kD
p-JNK
54kD
β-Actin
42kD
37kD
L6
Insulin - + + +
BMDMs - - WT MKO
kD
75kD
p-JNK
54kD
β-Actin
42kD
37kD

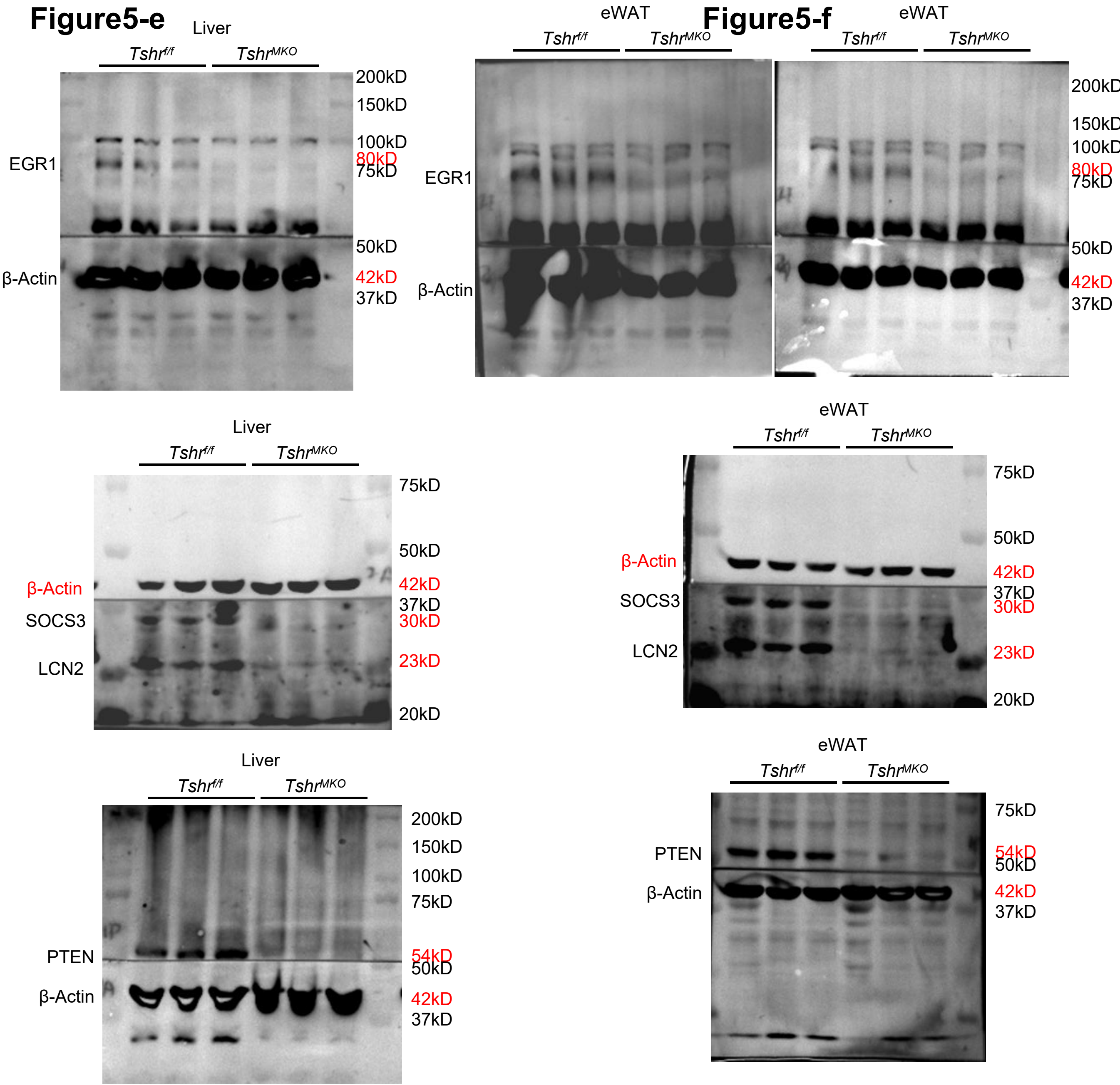
Figure5-e
Liver
Tshr^f/f
Tshr^MKO
200kD
150kD
100kD
80kD
75kD
50kD
42kD
37kD
EGR1
β-Actin
Figure5-f
eWAT
Tshr^f/f
Tshr^MKO
EGR1
β-Actin
SOCS3
LCN2
PTEN
75kD
54kD
30kD
23kD
20kD

Figure5-g

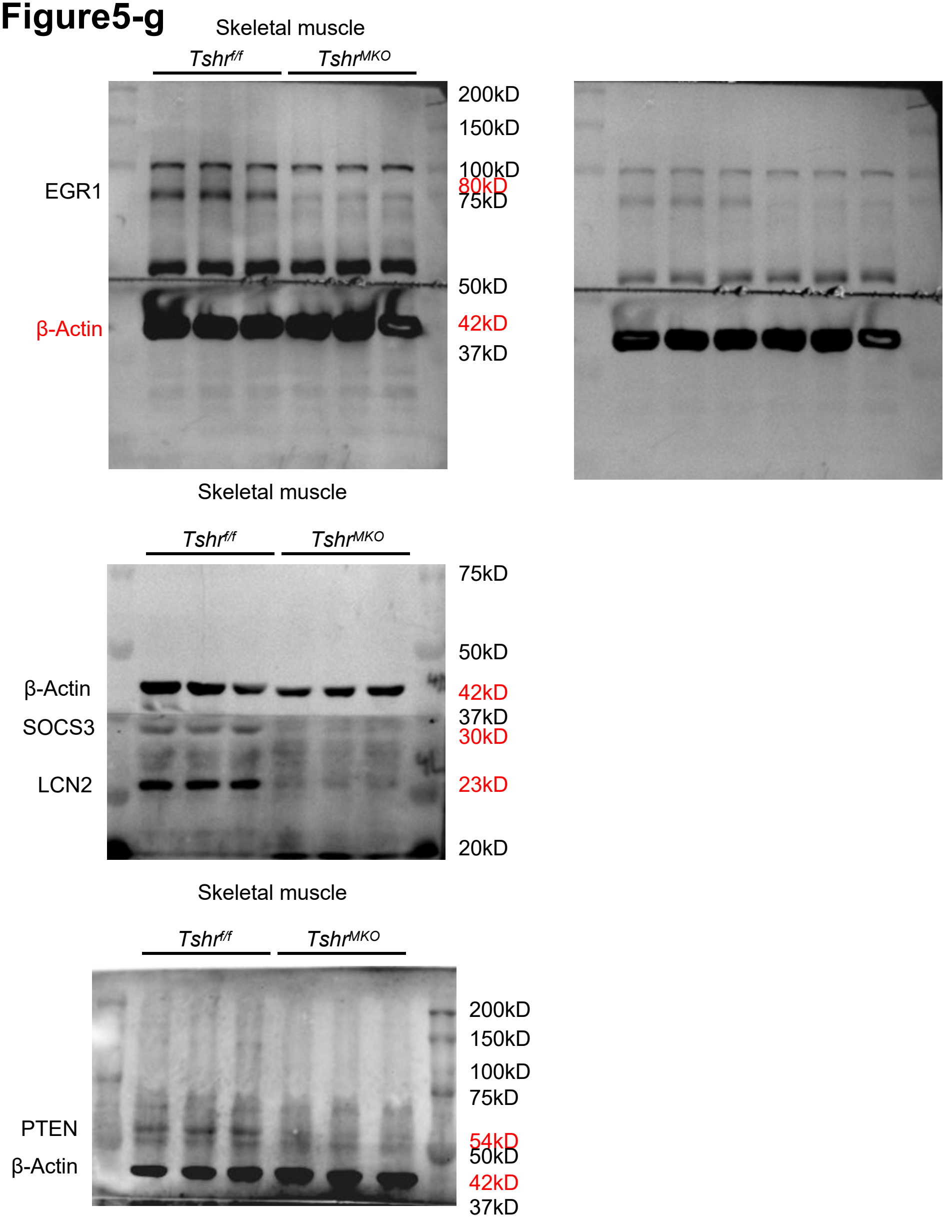
Skeletal muscle
Tshr f/f
Tshr MKO
EGR1
β-Actin
200kD
150kD
100kD
80kD
75kD
50kD
42kD
37kD
Skeletal muscle
Tshr f/f
Tshr MKO
β-Actin
SOCS3
LCN2
75kD
50kD
42kD
37kD
30kD
23kD
20kD
Skeletal muscle
Tshr f/f
Tshr MKO
PTEN
β-Actin
200kD
150kD
100kD
75kD
54kD
50kD
42kD
37kD

Figure6-a

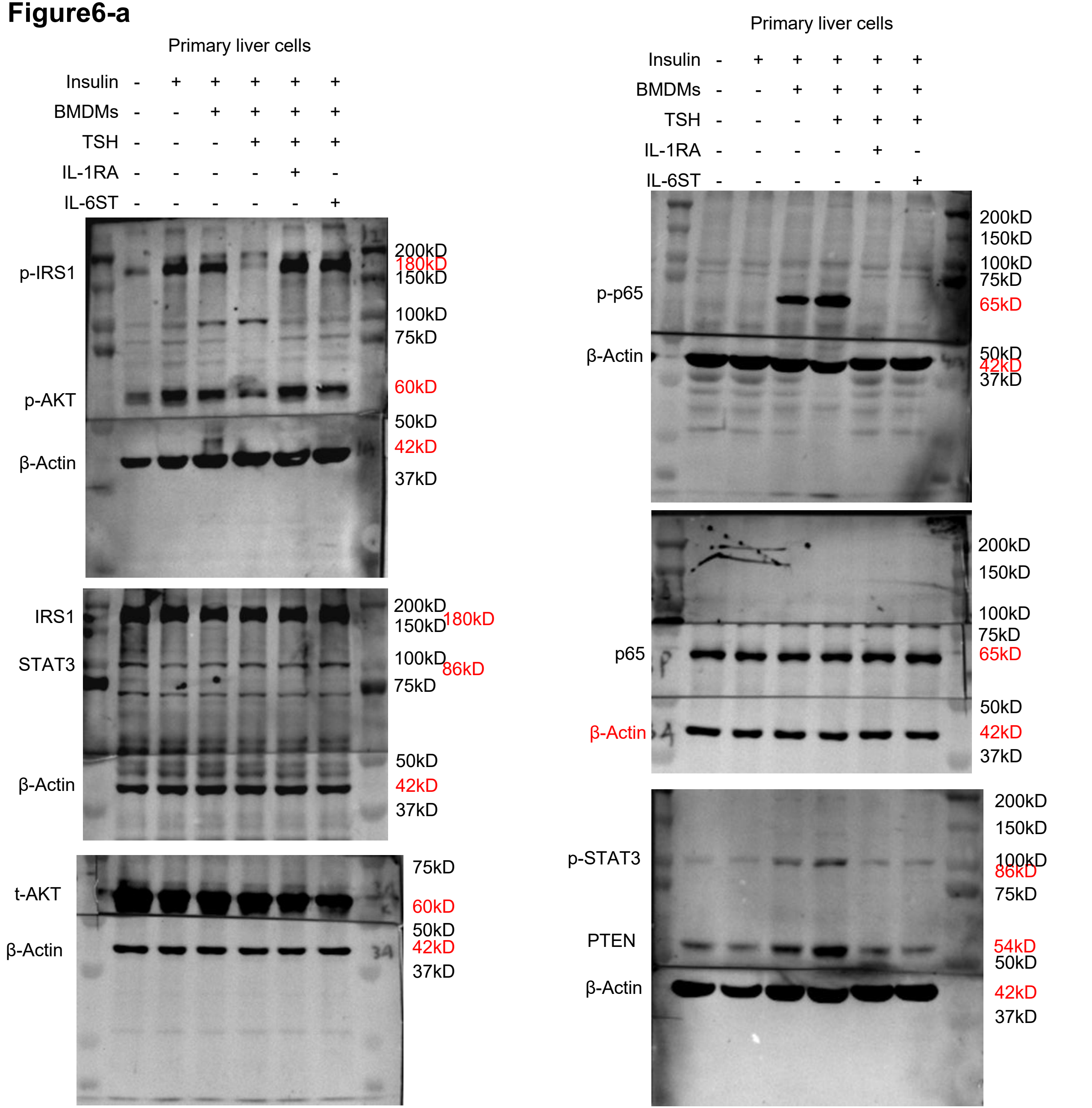
Primary liver cells
Insulin - + + + + +
BMDMs - - + + + +
TSH - - - + + +
IL-1RA - - - - + -
IL-6ST - - - - - +
p-IRS1
p-AKT
β-Actin
200kD
180kD
150kD
100kD
75kD
60kD
50kD
42kD
37kD
IRS1
STAT3
β-Actin
200kD
150kD
180kD
100kD
86kD
75kD
50kD
42kD
37kD
t-AKT
β-Actin
75kD
60kD
50kD
42kD
37kD
Primary liver cells
Insulin - + + + + +
BMDMs - - + + + +
TSH - - - + + +
IL-1RA - - - - + -
IL-6ST - - - - - +
p-p65
β-Actin
200kD
150kD
100kD
75kD
65kD
50kD
42kD
37kD
p65
β-Actin
200kD
150kD
100kD
75kD
65kD
50kD
42kD
37kD
p-STAT3
PTEN
β-Actin
200kD
150kD
100kD
86kD
75kD
54kD
50kD
42kD
37kD

Figure6-a

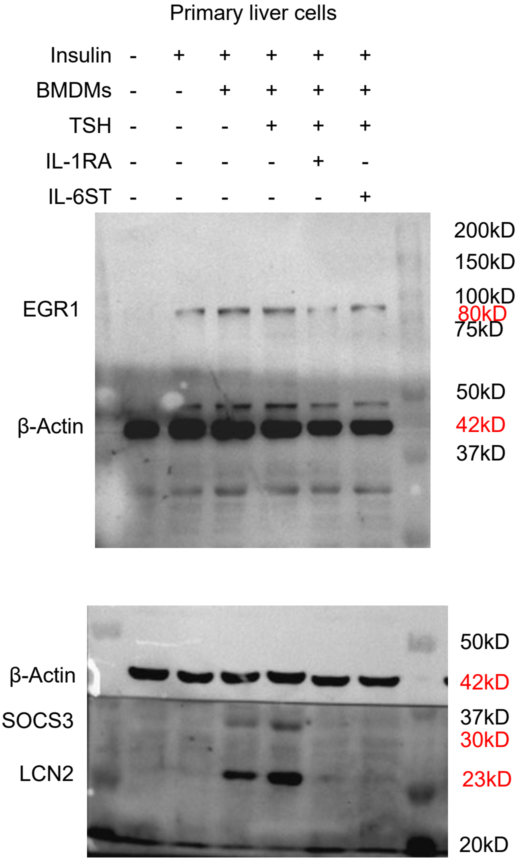

Figure6-b

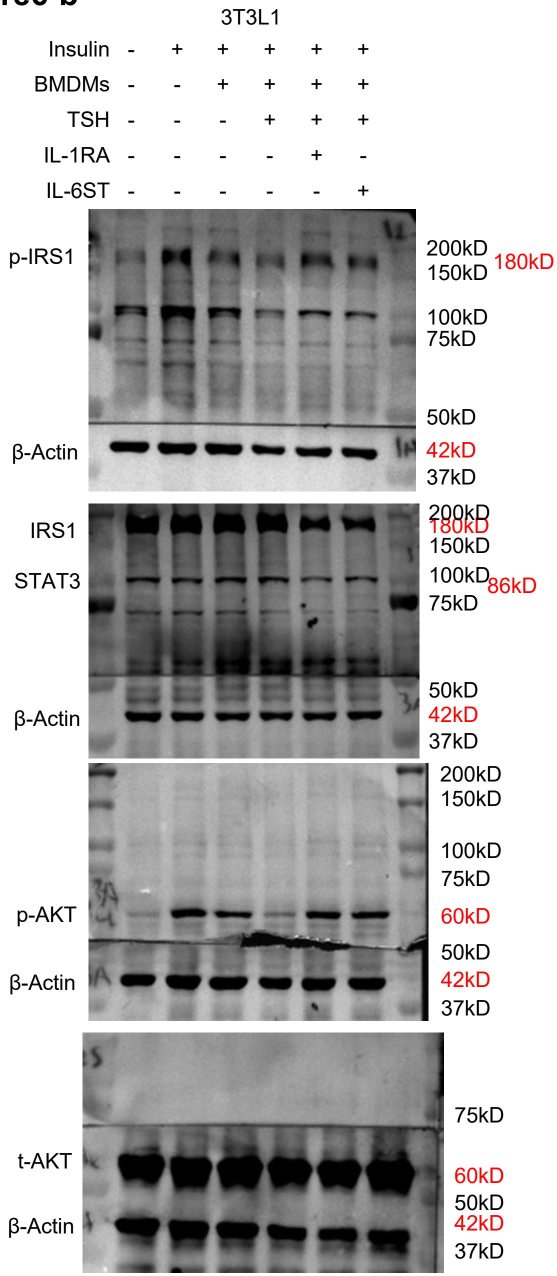

Figure6-b

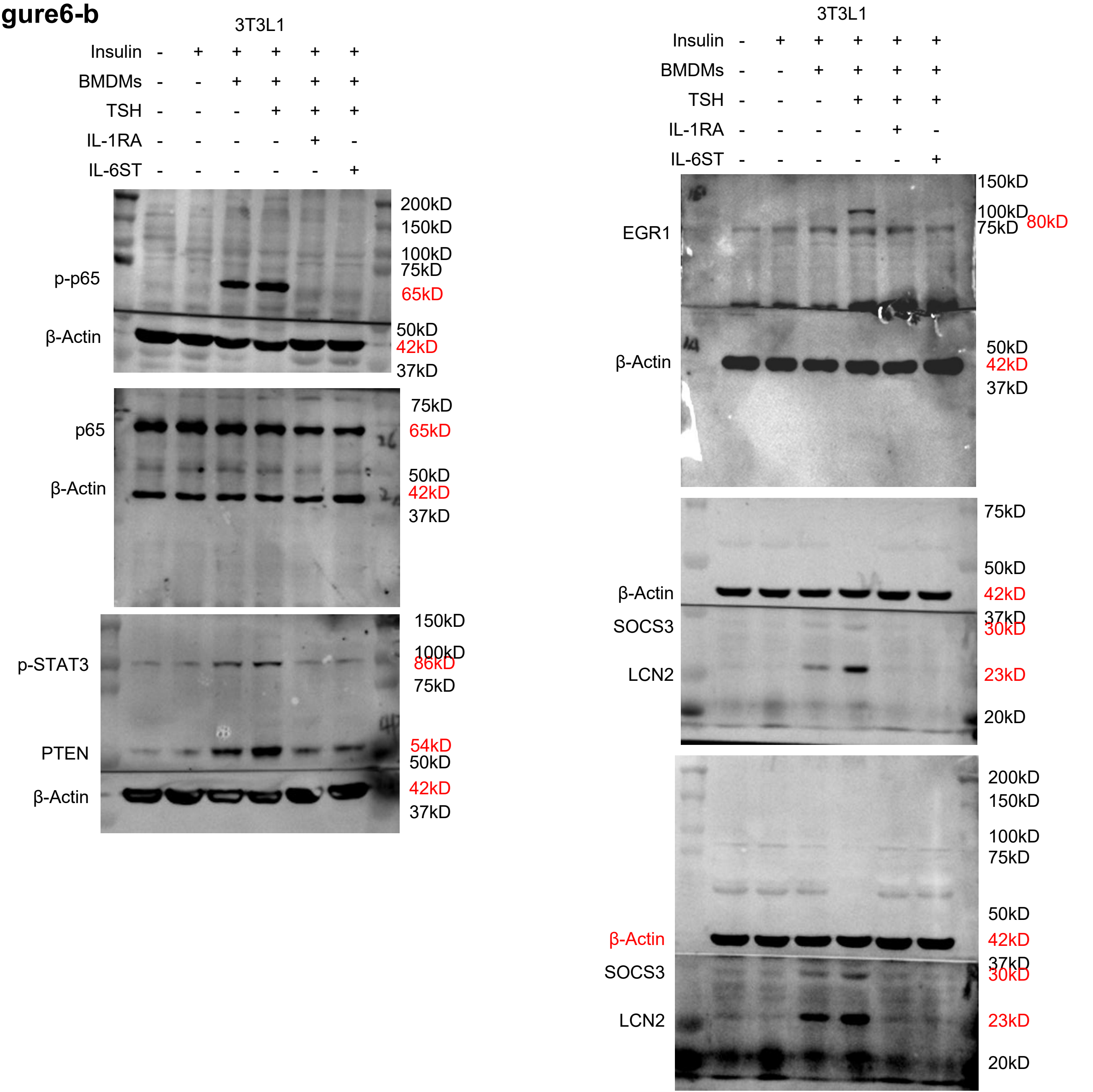

3T3L1
Insulin - + + + + +
BMDMs - - + + + +
TSH - - - + + +
IL-1RA - - - - + -
IL-6ST - - - - - +
p-p65
β-Actin
200kD
150kD
100kD
75kD
65kD
50kD
42kD
37kD
p65
β-Actin
75kD
65kD
50kD
42kD
37kD
p-STAT3
PTEN
β-Actin
150kD
100kD
86kD
75kD
54kD
50kD
42kD
37kD
3T3L1
Insulin - + + + + +
BMDMs - - + + + +
TSH - - - + + +
IL-1RA - - - - + -
IL-6ST - - - - - +
EGR1
β-Actin
150kD
100kD
80kD
75kD
50kD
42kD
37kD
β-Actin
SOCS3
LCN2
75kD
50kD
42kD
37kD
30kD
23kD
20kD
β-Actin
SOCS3
LCN2
200kD
150kD
100kD
75kD
50kD
42kD
37kD
30kD
23kD
20kD

Figure6-c

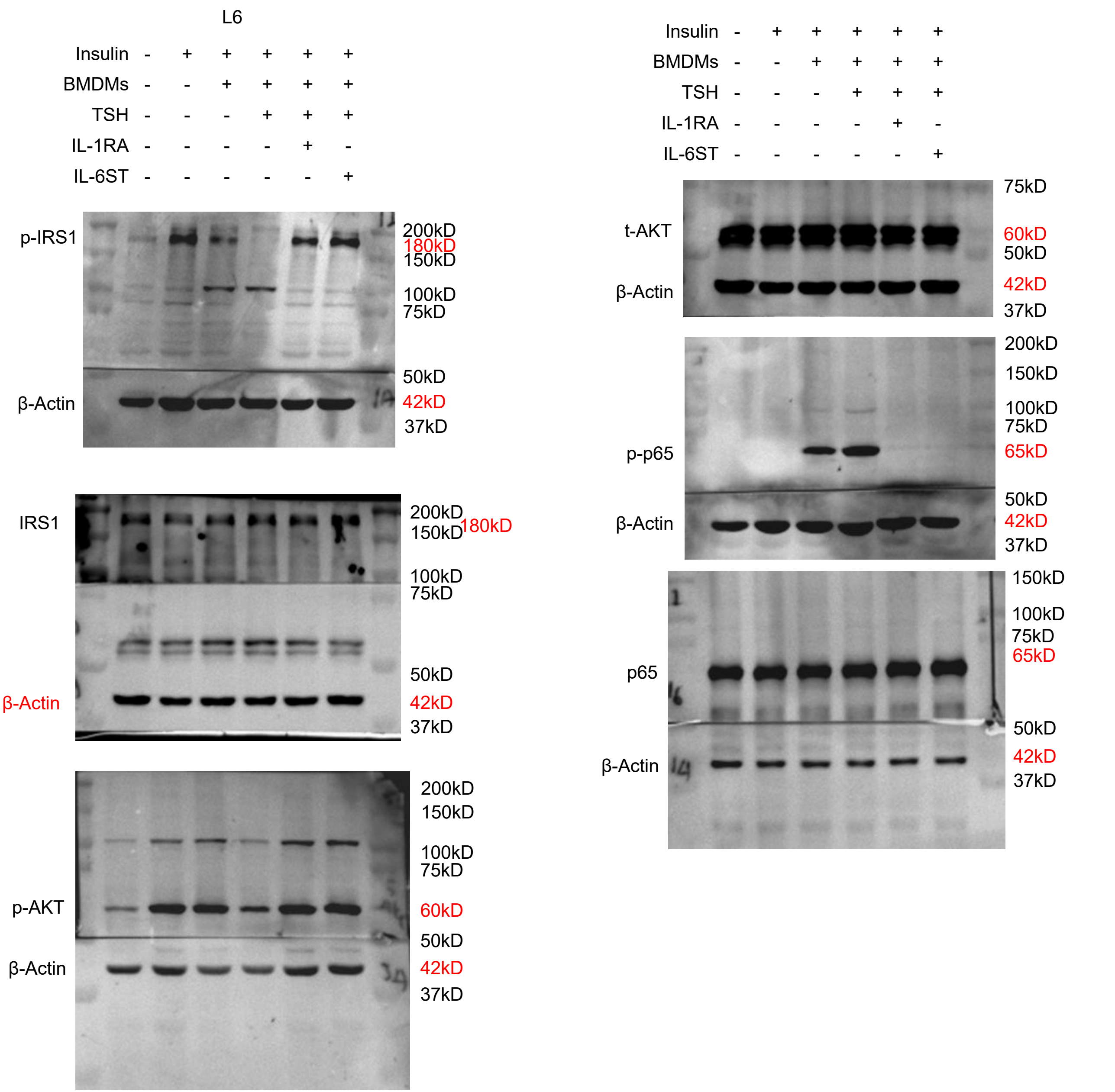

L6
Insulin - + + + + +
BMDMs - - + + + +
TSH - - - + + +
IL-1RA - - - - + -
IL-6ST - - - - - +
p-IRS1
β-Actin
200kD
180kD
150kD
100kD
75kD
50kD
42kD
37kD
IRS1
β-Actin
200kD
150kD
180kD
100kD
75kD
50kD
42kD
37kD
p-AKT
β-Actin
200kD
150kD
100kD
75kD
60kD
50kD
42kD
37kD
L6
Insulin - + + + + +
BMDMs - - + + + +
TSH - - - + + +
IL-1RA - - - - + -
IL-6ST - - - - - +
t-AKT
β-Actin
75kD
60kD
50kD
42kD
37kD
p-p65
β-Actin
200kD
150kD
100kD
75kD
65kD
50kD
42kD
37kD
p65
β-Actin
150kD
100kD
75kD
65kD
50kD
42kD
37kD

Figure6-c

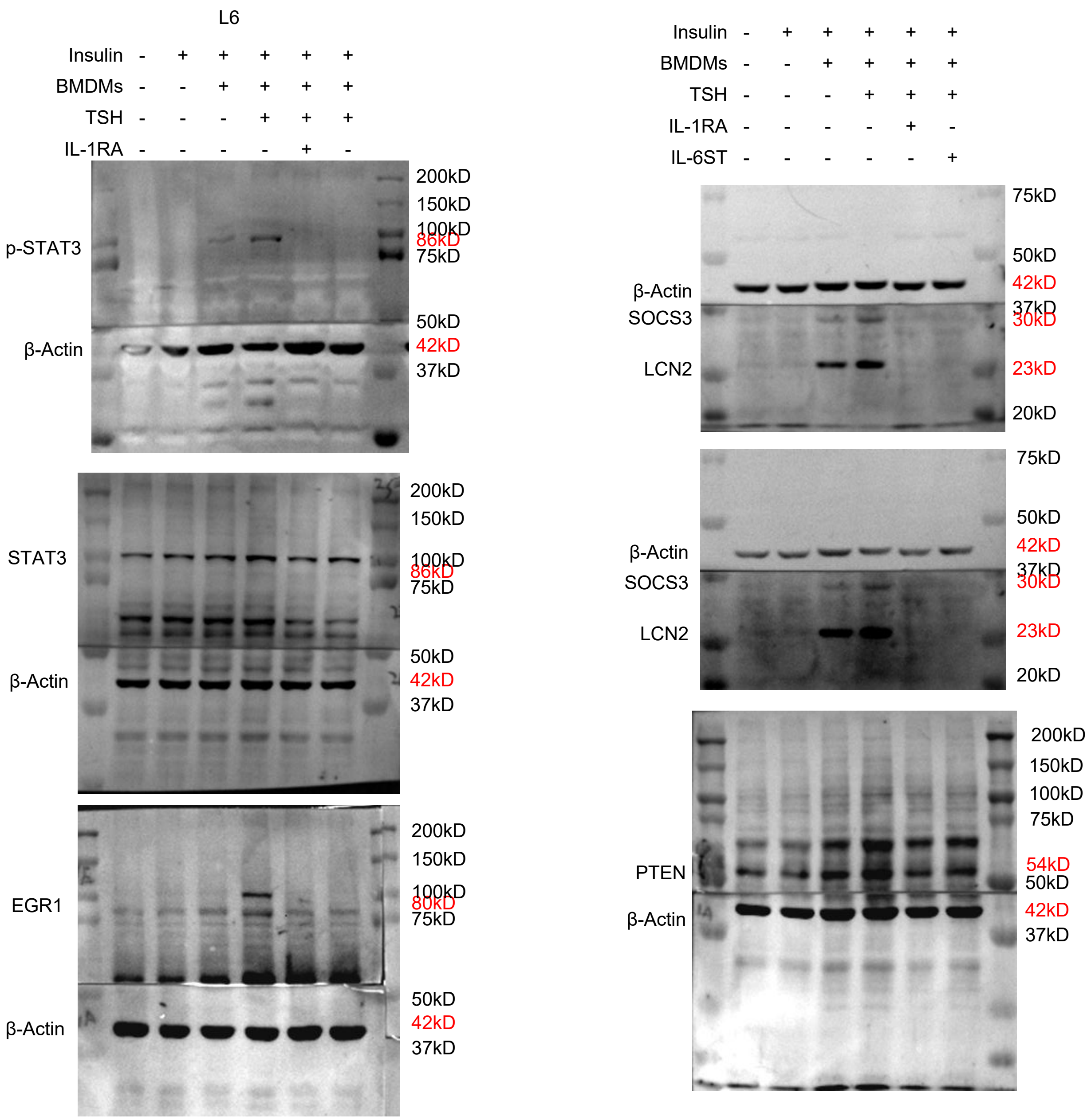
L6
Insulin - + + + + +
BMDMs - - + + + +
TSH - - - + + +
IL-1RA - - - - + -
p-STAT3
β-Actin
200kD
150kD
100kD
86kD
75kD
50kD
42kD
37kD
STAT3
β-Actin
200kD
150kD
100kD
86kD
75kD
50kD
42kD
37kD
EGR1
β-Actin
200kD
150kD
100kD
80kD
75kD
50kD
42kD
37kD
L6
Insulin - + + + + +
BMDMs - - + + + +
TSH - - - + + +
IL-1RA - - - - + -
IL-6ST - - - - - +
β-Actin
SOCS3
LCN2
75kD
50kD
42kD
37kD
30kD
23kD
20kD
β-Actin
SOCS3
LCN2
75kD
50kD
42kD
37kD
30kD
23kD
20kD
PTEN
β-Actin
200kD
150kD
100kD
75kD
54kD
50kD
42kD
37kD

Figure7-a

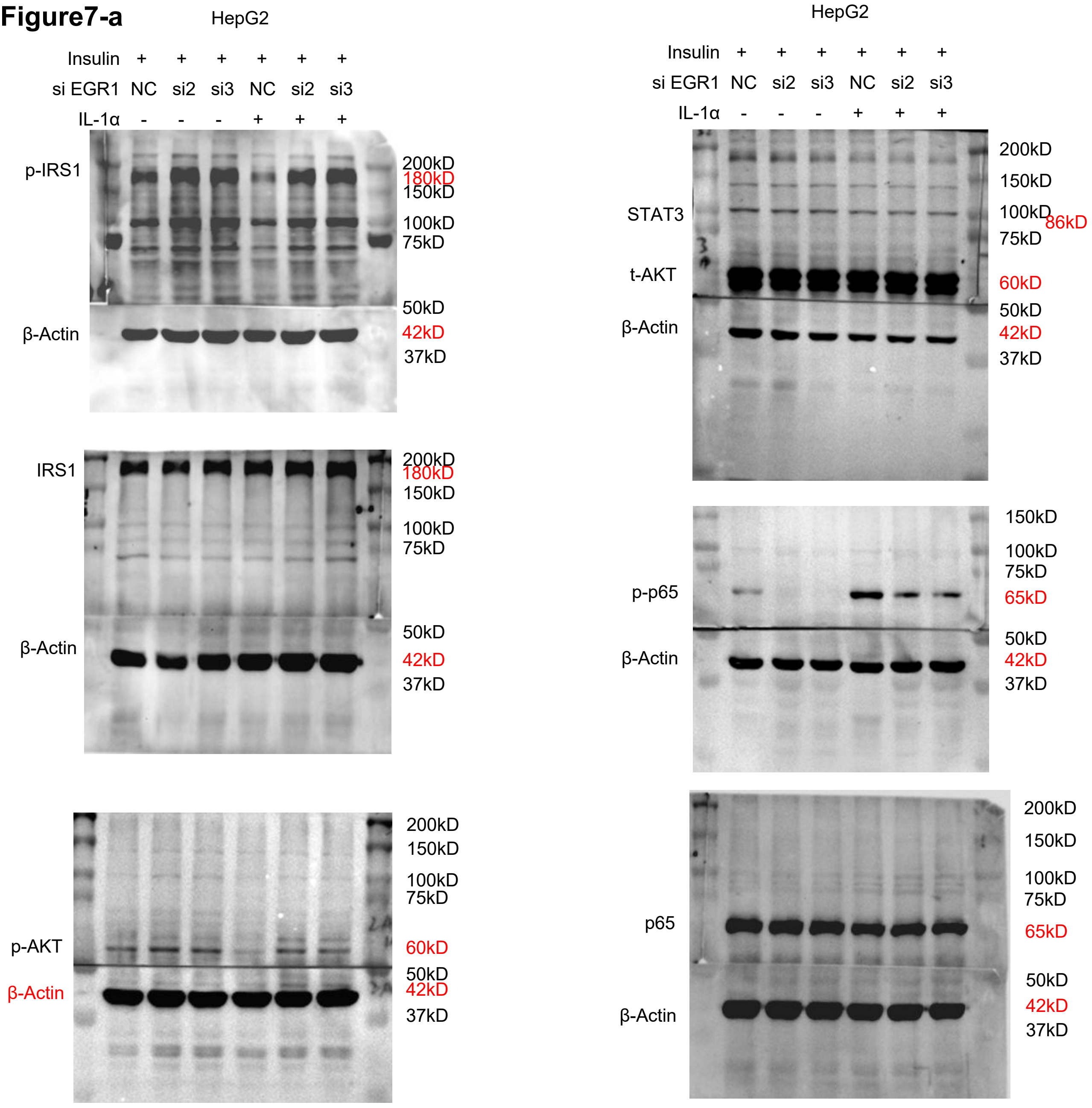
HepG2
Insulin + + + + + +
si EGR1 NC si2 si3 NC si2 si3
IL-1α - - - + + +
p-IRS1
β-Actin
200kD
180kD
150kD
100kD
75kD
50kD
42kD
37kD
IRS1
β-Actin
200kD
180kD
150kD
100kD
75kD
50kD
42kD
37kD
p-AKT
β-Actin
200kD
150kD
100kD
75kD
60kD
50kD
42kD
37kD
HepG2
Insulin + + + + + +
si EGR1 NC si2 si3 NC si2 si3
IL-1α - - - + + +
STAT3
t-AKT
β-Actin
200kD
150kD
100kD
86kD
75kD
60kD
50kD
42kD
37kD
p-p65
β-Actin
150kD
100kD
75kD
65kD
50kD
42kD
37kD
p65
β-Actin
200kD
150kD
100kD
75kD
65kD
50kD
42kD
37kD

Figure7-a

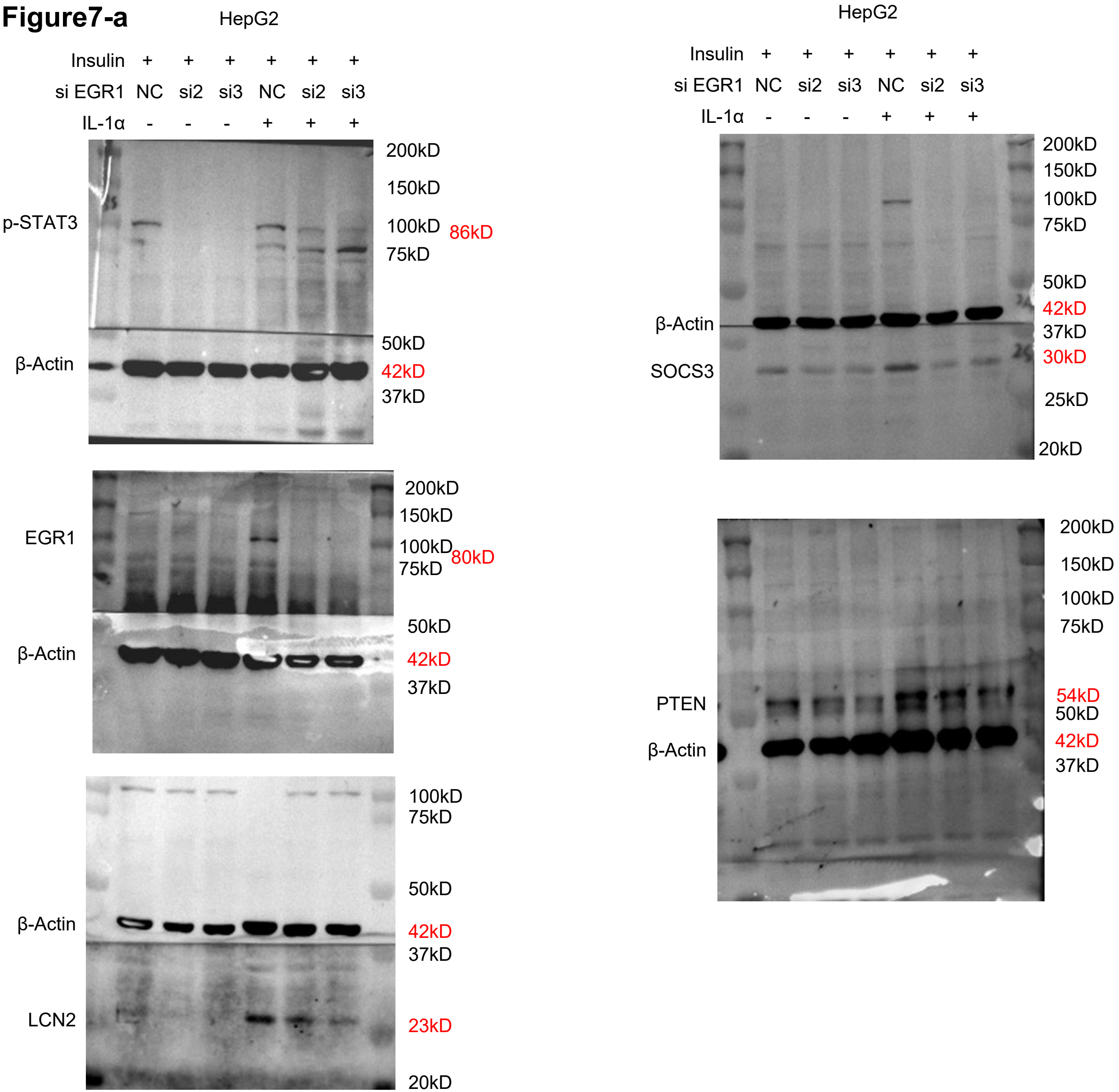
HepG2
Insulin + + + + + +
si EGR1 NC si2 si3 NC si2 si3
IL-1α - - - + + +
p-STAT3
β-Actin
200kD
150kD
100kD
86kD
75kD
50kD
42kD
37kD
EGR1
β-Actin
200kD
150kD
100kD
80kD
75kD
50kD
42kD
37kD
β-Actin
LCN2
100kD
75kD
50kD
42kD
37kD
23kD
20kD
HepG2
Insulin + + + + + +
si EGR1 NC si2 si3 NC si2 si3
IL-1α - - - + + +
β-Actin
SOCS3
200kD
150kD
100kD
75kD
50kD
42kD
37kD
30kD
25kD
20kD
PTEN
β-Actin
200kD
150kD
100kD
75kD
54kD
50kD
42kD
37kD

Figure7-d

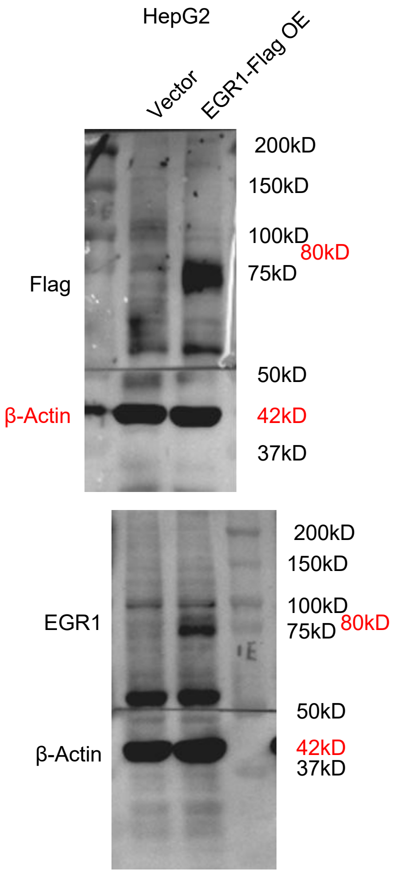
HepG2
Vector
EGR1-Flag OE
Flag
β-Actin
200kD
150kD
100kD
80kD
75kD
50kD
42kD
37kD
EGR1
β-Actin
200kD
150kD
100kD
80kD
75kD
50kD
42kD
37kD

Supplemental Figure1-c

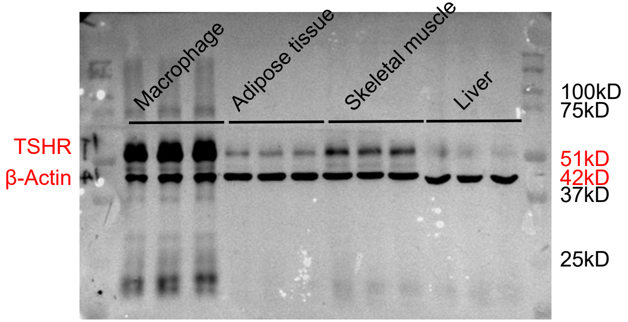

Supplemental Figure2-a

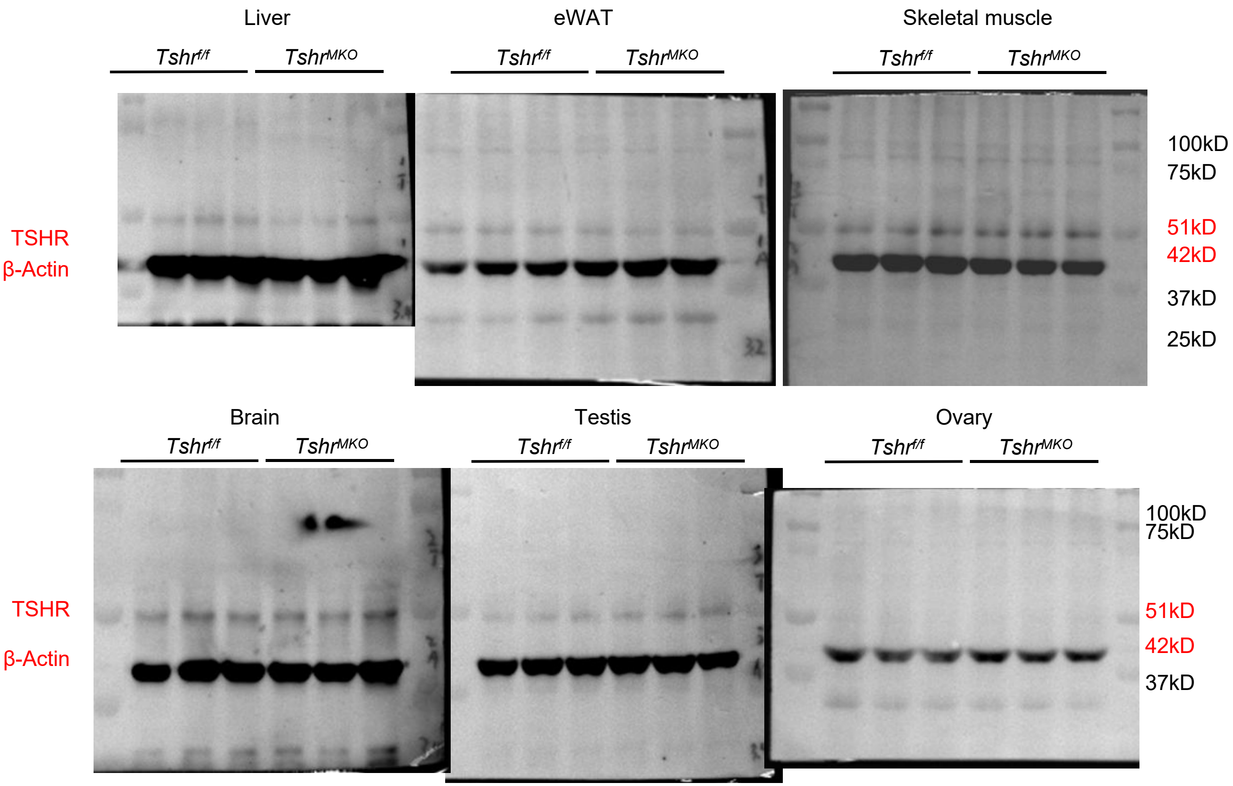

Supplemental Figure2-e

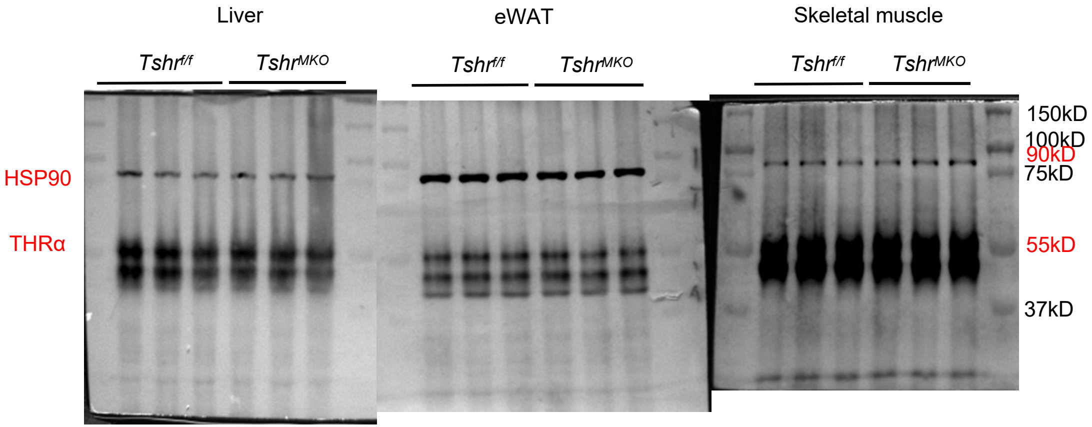

Supplemental Figure 13

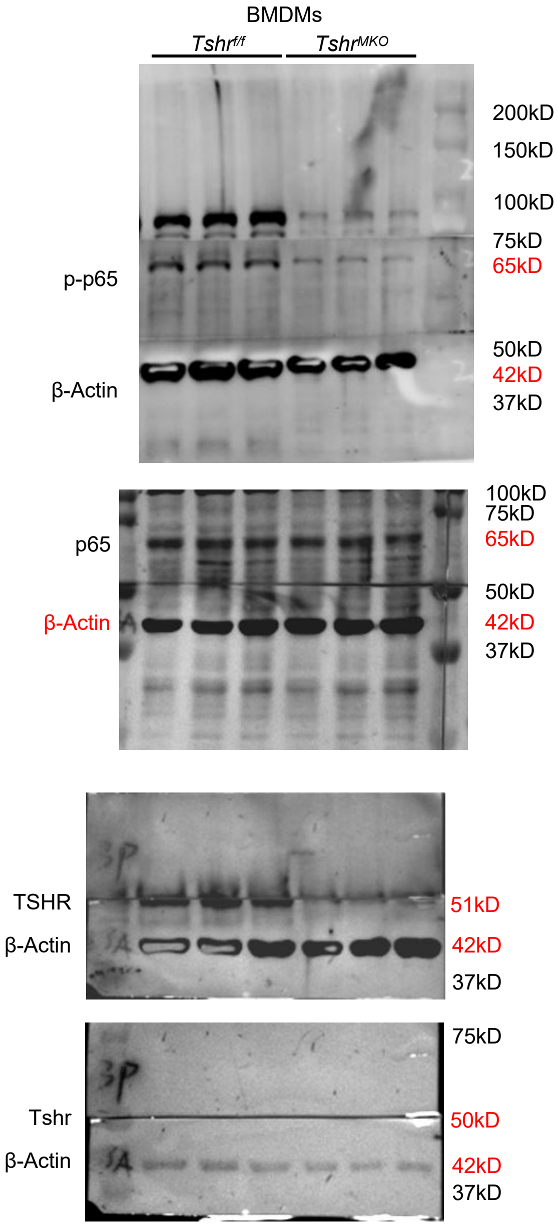

Fig 3 a and b

Hepatic infiltrating macrophage

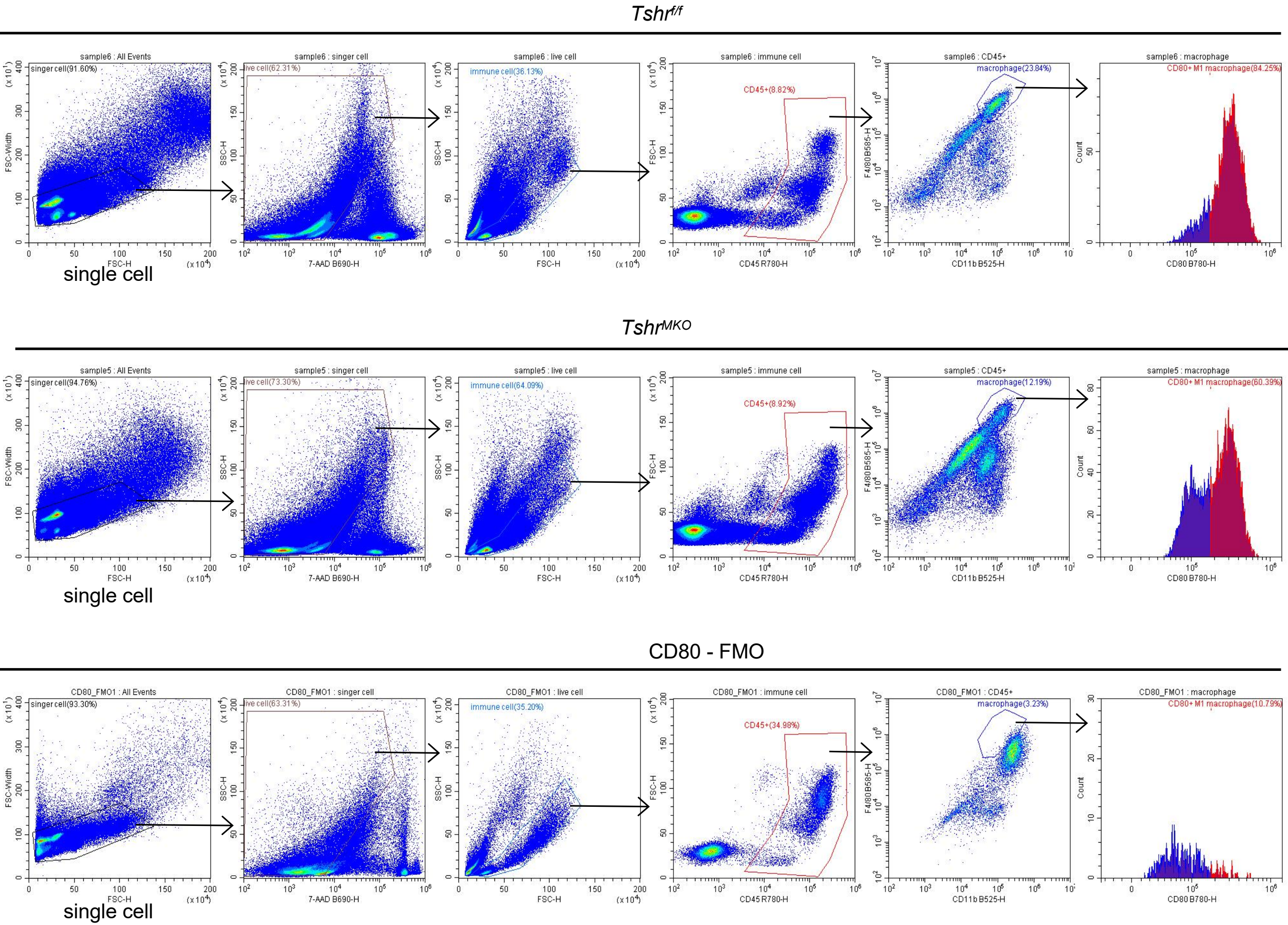

Fig 3 a and b

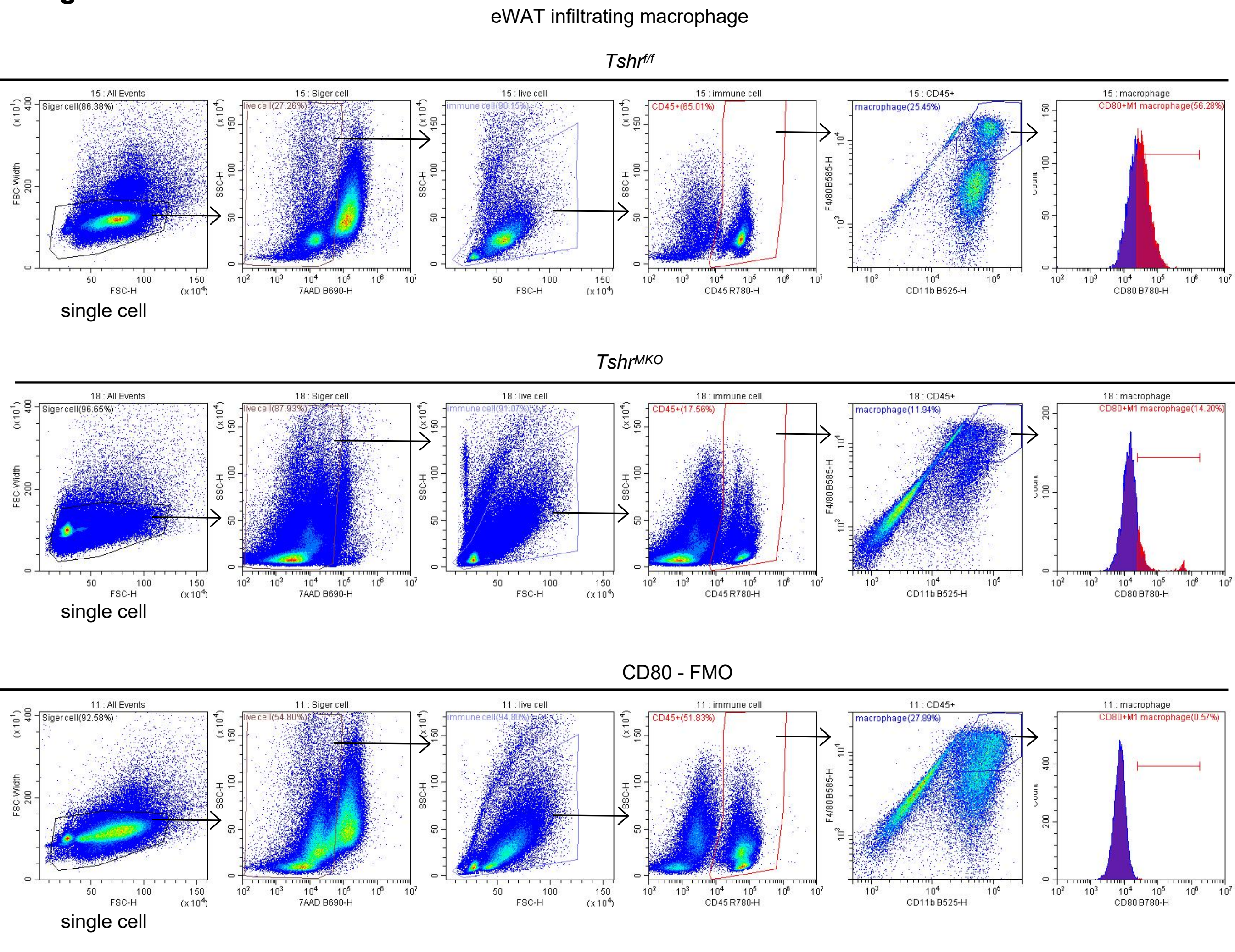
eWAT infiltrating macrophage
Tshr f/f
15 : All Events
Siger cell(86.38%)
15 : Siger cell
live cell(27.26%)
15 : live cell
immune cell(90.15%)
15 : immune cell
CD45+(65.01%)
15 : CD45+
macrophage(25.45%)
15 : macrophage
CD80+M1 macrophage(56.28%)
single cell
Tshr MKO
18 : All Events
Siger cell(96.65%)
18 : Siger cell
live cell(87.93%)
18 : live cell
immune cell(91.07%)
18 : immune cell
CD45+(17.56%)
18 : CD45+
macrophage(11.94%)
18 : macrophage
CD80+M1 macrophage(14.20%)
single cell
CD80 - FMO
11 : All Events
Siger cell(92.58%)
11 : Siger cell
live cell(54.80%)
11 : live cell
immune cell(94.80%)
11 : immune cell
CD45+(51.83%)
11 : CD45+
macrophage(27.89%)
11 : macrophage
CD80+M1 macrophage(0.57%)
single cell
FSC-Width
FSC-H
SSC-H
7AAD B690-H
CD45 R780-H
F4/80 B585-H
CD11b B525-H
CD80 B780-H

Fig 3 a and b

Skeletal muscle infiltrating macrophage

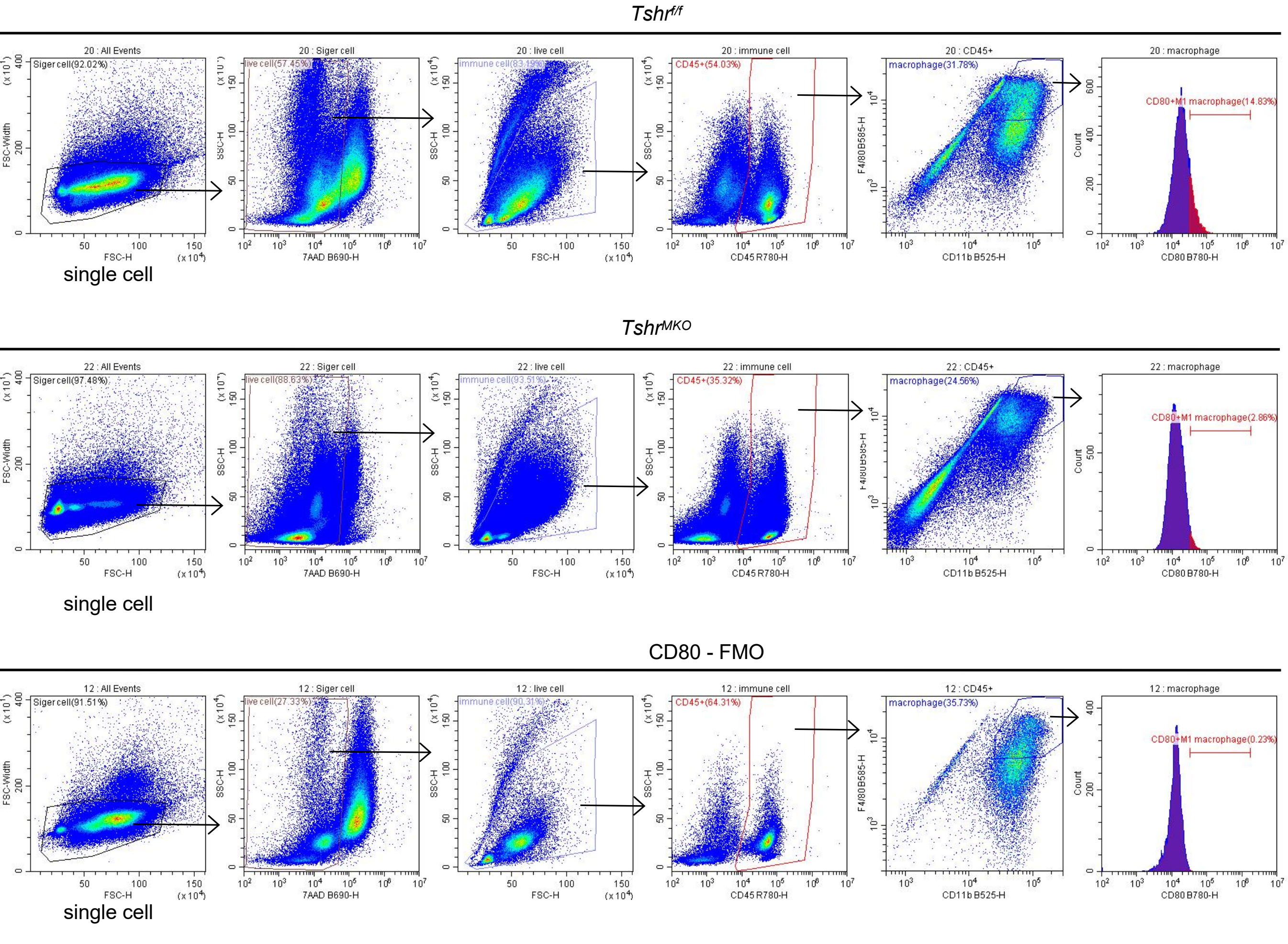

Fig 4 a

BMDMs M1 polarization

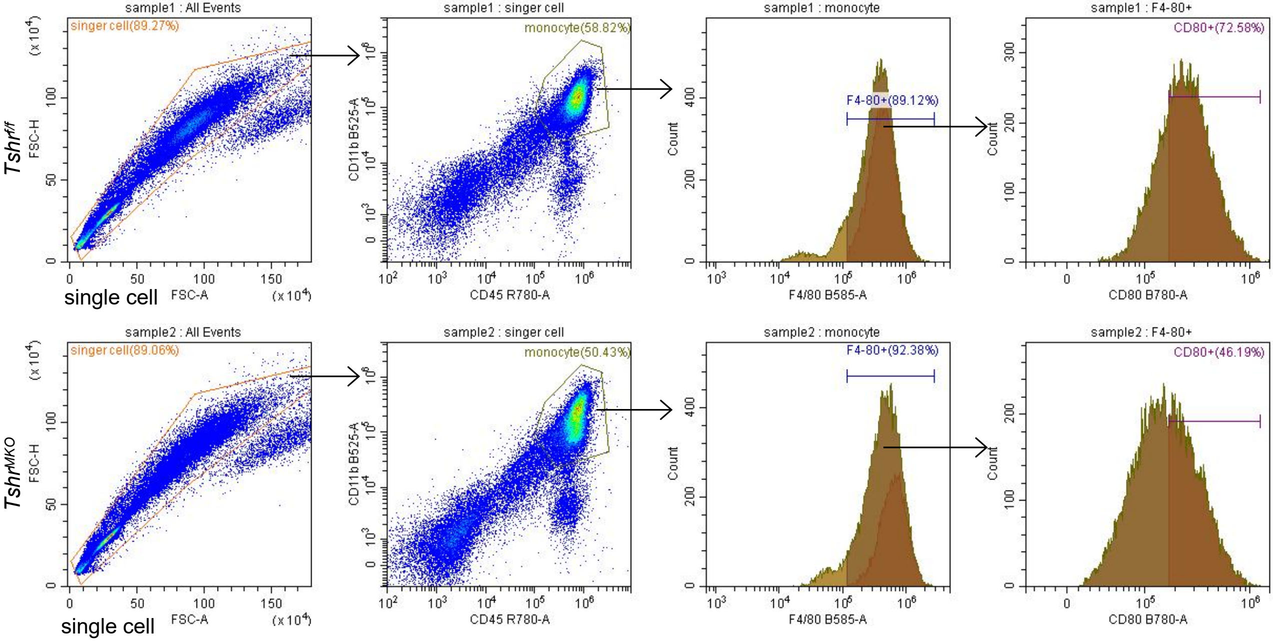

Fig 4 b

BMDMs Intracellular ROS levels

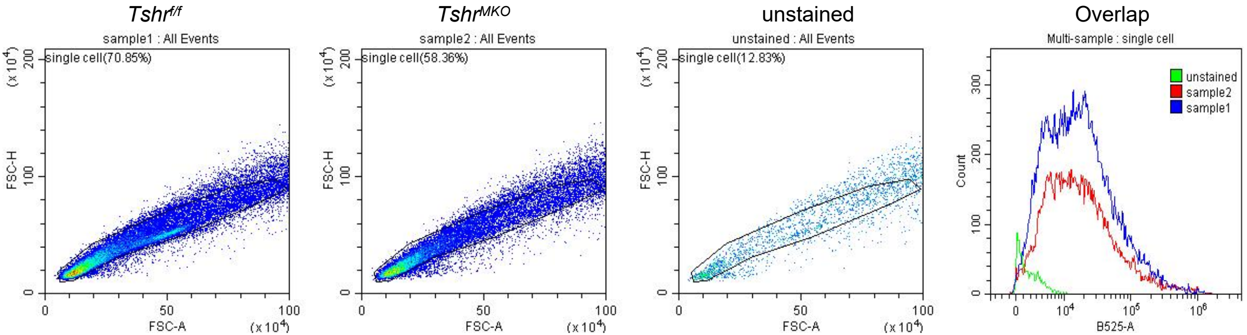

Fig 8 k

PBMC derived macrophages   Control

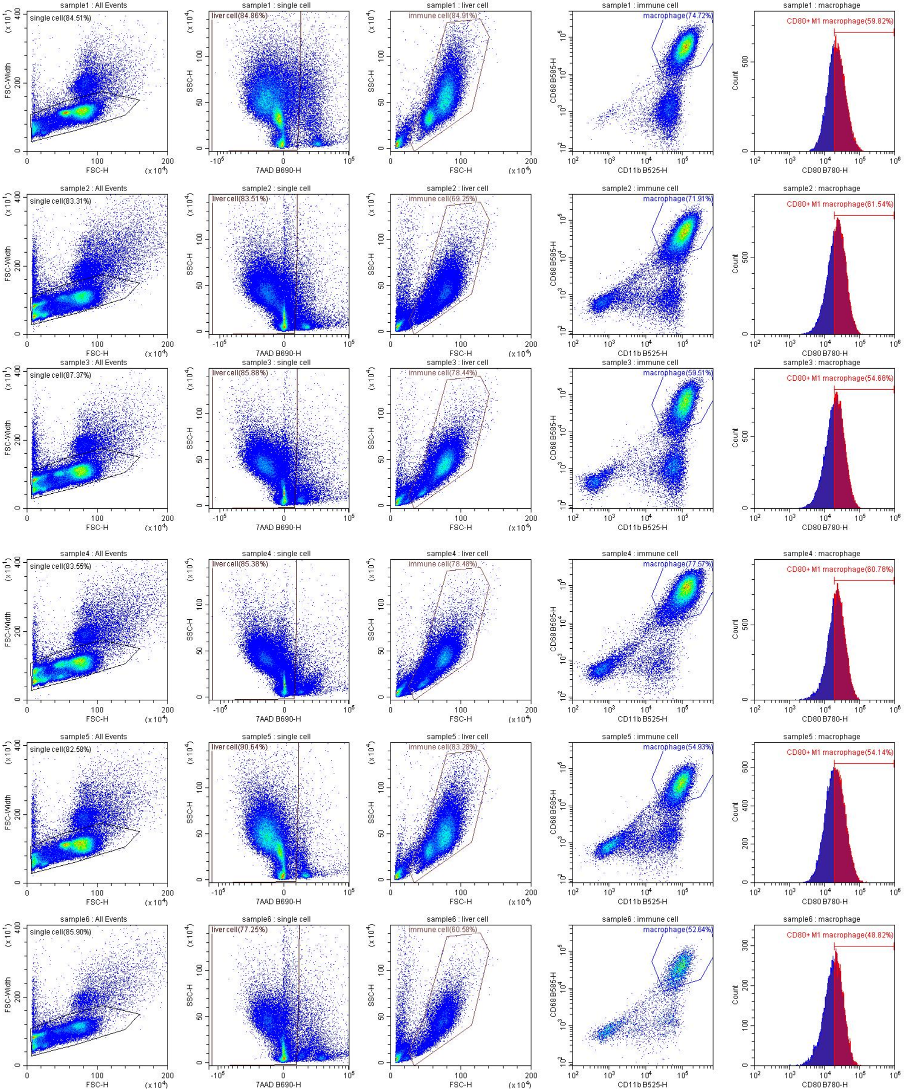

Fig 8 k

PBMC derived macrophages SH

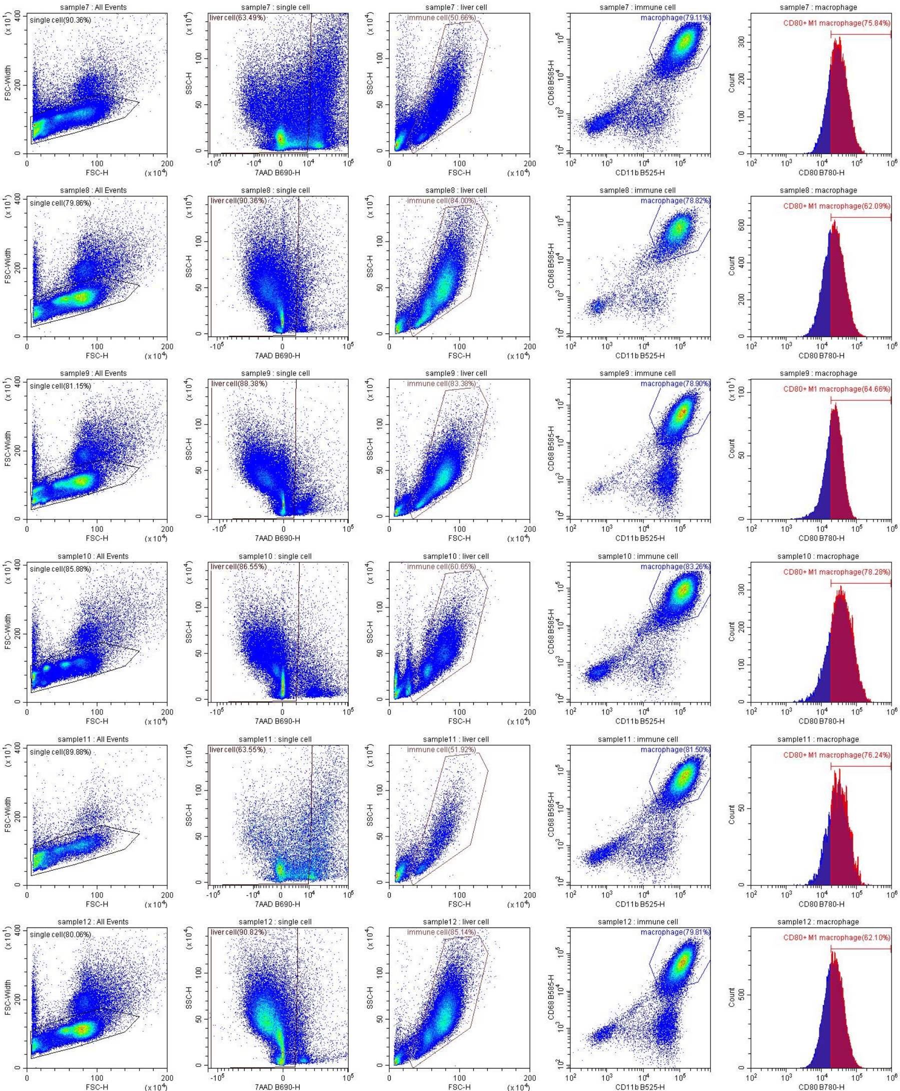

Supplement: Supplementary file 2 — Raw Imaging [file 12276_2025_1478_MOESM2_ESM.pdf]
